# Supplementary material for: Neoadjuvant and adjuvant pembrolizumab in advanced high-grade serous carcinoma: the randomized phase II NeoPembrOV clinical trial
Source: Nat Commun. 2024 Jul 16;15:5931. doi: 10.1038/s41467-024-46999-x (PMC11252284; doi:10.1038/s41467-024-46999-x)
Supplement: Supplementary file 1 — Supplementary Information [file 41467_2024_46999_MOESM1_ESM.pdf]

Neoadjuvant and adjuvant pembrolizumab in advanced high-grade serous carcinoma: the randomized phase II NeoPembrOV clinical trial

Isabelle L. Ray-Coquard, et al.

Supplementary materials

**Supplementary Table 1. Baseline characteristics**		
**Characteristic**	**NACT alone (*n* = 30)**	**NACT + pembrolizumab (*n* = 61)**
**Median age, years (range)**	61.5 (40–79)	63 (42–76)
**ECOG PS**		
0	14 (47)	29 (48)
1	15 (50)	32 (52)
2	1 (3)	0
**Histologic type**		
High-grade serous	28 (93)	60 (98)
Other	2 (7)[a]	1 (2)[b]
**Primary site**		
Ovary	30 (100)	54 (89)
Peritoneum	0	7 (11)
**FIGO staging**		
IIIC	25 (83)	50 (82)
IV	5 (17)	11 (18)
**Mean CA-125, U/mL (SD)**	880.4 (936.4)	1385.1 (1935.1)
**Initial diagnostic laparoscopy**	29 (97)	53 (87)
BRCA mutant[c]		
Yes	4 (13)	15 (25)
No	25 (83)	43 (70)
Unknown	1 (3)	3 (5)
**HRD status[d]**		
HRD	8 (27)	19 (31)
Non-HRD	15 (50)	29 (48)
Unknown	7 (23)	13 (21)
**PD-L1 status**		
CPS ≥10	11 (37)	19 (31)
CPS ≥1	18 (60)	39 (64)
Unknown	0	6 (10)
**Bevacizumab planned**	29 (97)	59 (97)

Data are no. (%) unless otherwise noted. [a]Low-grade serous (*n* = 1), clear cell (*n* = 1). [b]Undifferentiated. [c]Germline and/or somatic. In the NACT-alone arm, 2 patients had a somatic *BRCA1* deleterious mutation and 1 had a germline *BRCA2* deleterious mutation. In the pembrolizumab + NACT arm, 3 patients had a germline *BRCA1* mutation, 4 had a somatic *BRCA1* mutation, 2 had both germline and somatic *BRCA1* mutations, 1 had a germline *BRCA2* mutation, and 4 had a somatic *BRCA2* mutation. [d]HRD status determined using the shallowHRDv2 assay. CA-125, cancer antigen-125; CPS, combined positive score; ECOG PS, Eastern Cooperative Oncology Group performance status; FIGO, International Federation of Gynecology and Obstetrics; HRD, homologous recombination deficiency; NACT, neoadjuvant chemotherapy; PD-L1, programmed death ligand-1; SD, standard deviation.

Supplementary Table 2. Postoperative complications[a]		
**Postoperative complication**	**CP alone (n = 30)**	**CP + pembrolizumab (n = 61)**
Any postoperative complication	4 (13)	13 (21)
Gastrointestinal fistula	1 (3) [grade II]	1 (2) [grade II]
Hemorrhage	1 (3) [grade II]	0
Infection/post-surgical fever	0	4 (7) [two grade II, two grade IIIb]
Phlebitis	0	1 (2) [grade II]
Stoma complication	0	1 (2) [grade V]
Ascites	0	1 (2)
Chyloperitoneum	0	1 (2)
Lymphocele	0	2 (3) [grade IIIb, grade III]
Occlusive syndrome	0	1 (2) [grade II]
Paresthesia and left leg hypoesthesia	1 (3)	0
Pleural effusion	1 (3) [grade IIIb]	1 (2) [grade II]
Psoas sideration	0	1 (2) [grade I]
Rectovaginal fistula	0	1 (2) [grade I]
Right shutter neuralgia	1 (3) [grade I]	0
Subphrenic abscess	0	1 (2) [grade II]
Vomiting	1 (3) [grade II]	0
Difficult transit recovery	1 (3) [grade II]	0
Gastroparesis	0	1 (2)
Weight loss	1 (3) [grade I]	0
Diaphragm pain	0	1 (2) [grade I]

Data are no. (%). [a]Patient could have more than 1 complication. CP, carboplatin + paclitaxel.

Supplementary Table 3. Adverse events across the entire treatment period (any grade in ≥15%, grade ≥3 in ≥5%)

	**CP alone (n = 30)**		**CP + pembrolizumab (n = 61)**	
**Adverse event**	**Any grade**	**Grade ≥3**	**Any grade**	**Grade ≥3**
**Blood and lymphatic system disorders**				
Anemia	17 (57)	5 (17)	33 (54)	7 (11)
Neutropenia	12 (40)	7 (23)	24 (39)	14 (23)
Thrombocytopenia	8 (27)	2 (7)	18 (30)	4 (7)
Leukopenia	6 (20)	1 (3)	6 (10)	2 (3)
**Endocrine disorders**				
Hypothyroidism	2 (7)	0	17 (28)	1 (2)
Hyperthyroidism	0	0	9 (15)	0
**Gastrointestinal disorders**				
Nausea	12 (40)	0	33 (54)	0
Abdominal pain	12 (40)	2 (7)	25 (41)	1 (2)
Constipation	11 (37)	0	25 (41)	0
Diarrhea	11 (37)	0	23 (38)	2 (3)
Vomiting	6 (20)	1 (3)	12 (20)	1 (2)
Abdominal pain upper	6 (20)	1 (3)	10 (16)	0
Intestinal obstruction	1 (3)	1 (3)	4 (7)	4 (7)
**General disorders and administration-site reactions**				
Asthenia	19 (63)	0	42 (69)	3 (5)
Mucosal inflammation	1 (3)	0	9 (15)	0
Fatigue	5 (17)	0	4 (7)	0
**Infections and infestations**				
Urinary tract infection	4 (13)	0	13 (21)	1 (2)
**Investigations**				
Weight decreased	5 (17)	0	10 (16)	0
**Metabolism and nutrition disorders**				
Decreased appetite	4 (13)	0	9 (15)	2 (3)
**Musculoskeletal and connective tissue disorders**				
Arthralgia	14 (47)	0	31 (51)	1 (2)
Myalgia	2 (7)	0	11 (18)	0
Pain in extremity	5 (17)	0	8 (13)	1 (2)
**Nervous system disorders**				
Peripheral neuropathy	12 (40)	4 (13)	27 (44)	3 (5)
Headache	7 (23)	0	13 (21)	1 (2)
Paresthesia	3 (10)	0	11 (18)	0
**Respiratory, thoracic, and mediastinal disorders**				
Epistaxis	6 (20)	0	15 (25)	0
**Skin and subcutaneous tissue disorders**				
Alopecia	10 (33)	1 (3)	18 (30)	1 (2)
Rash	3 (10)	0	13 (21)	0
Pruritus	3 (10)	1 (3)	9 (15)	1 (2)

**Vascular disorders**				
Hypertension	6 (20)	0	16 (26)	4 (7)

Data are no. (%). CP, carboplatin + paclitaxel.

Supplementary Figure 1. CONSORT flow diagram. HIPEC, hyperthermic intraperitoneal chemotherapy; IDS, interval debulking surgery; NACT, neoadjuvant chemotherapy.

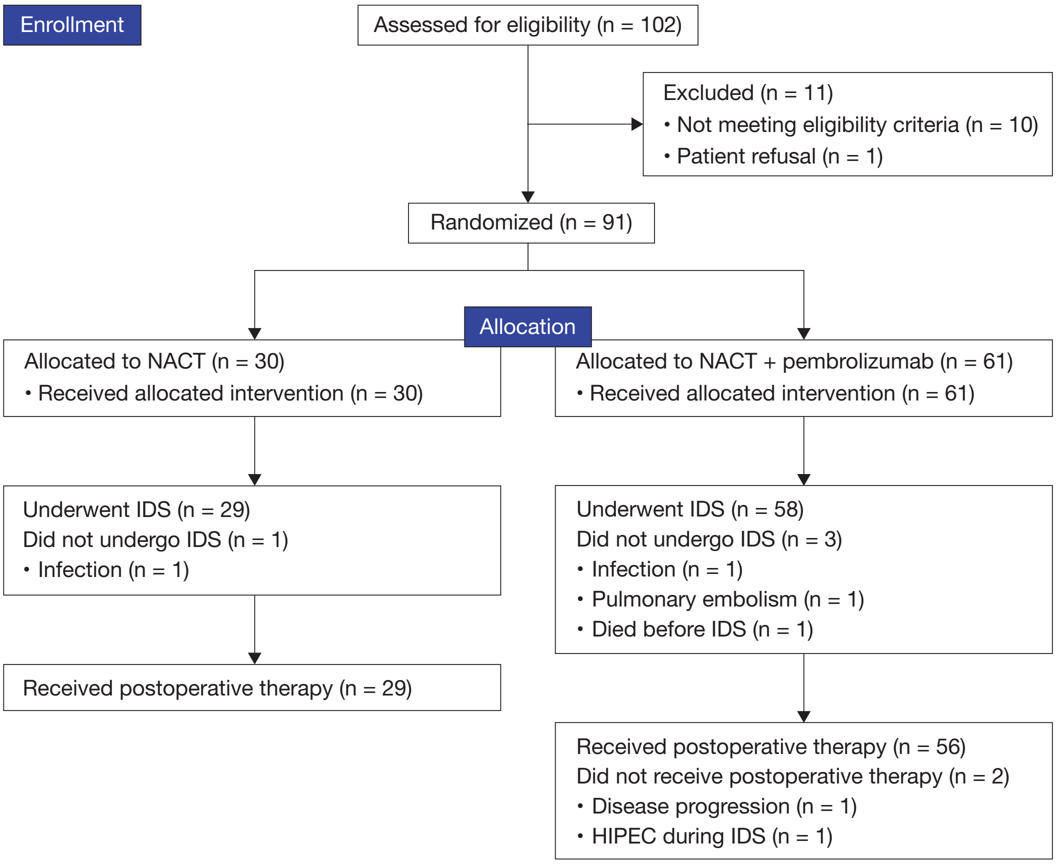

Supplementary Note 1.

A randomized, open-label, multicentric phase II trial of PEMBROLIZUMAB (Keytruda®) with chemotherapy versus chemotherapy alone (standard of care) as neo adjuvant treatment of ovarian cancer not amenable to front line debulking surgery.

NeoPembrOV PROTOCOL

NEO adjuvant **Pembro**lizumab in **OVar**ian carcinoma

GINECO-OV126b

N° EudraCT: 2016-004163-39

Version 3.0. dated on 03/06/2020

Coordinating Investigator	Pr Isabelle RAY-COQUARD<br>[redacted]
Study Biostatistician	Sylvie CHABAUD<br>[redacted]
Project Manager	[redacted]
Referent surgeon	[redacted]
Sponsor	ARCAGY-GINECO<br>(Association de Recherche sur les Cancers dont gynécologiques)<br>8 rue Lamennais<br>75008 Paris – FRANCE

SPONSOR SIGNATURE PAGE

Study title	A randomized, open-label, multicentric phase II trial of PEMBROLIZUMAB (MK-3475) with chemotherapy versus chemotherapy alone (standard of care) as neo adjuvant treatment of ovarian cancer not amenable to front line debulking surgery.

Version	**Version 3.0 dated on 03/06/2020**
Coordinating Investigator	[redacted]
Study Biostatistician	[redacted]
Sponsor	[redacted]

DOCUMENT HISTORY

Document-version	Version Date	Summary of changes
Original protocol - Version 1.0	04- 04- 2017	NA
Amendment 1- Version 2.0	21-01-2019	Clarification of inclusion criteria, amended management of pembrolizumab adverse events, edit
Amendment 2 – Version 3.0	03-06-2020	Modification of the primary endpoint, addition of two secondary endpoints and one exploratory criterion

TABLE OF CONTENTS

SPONSOR SIGNATURE PAGE........ 2
TABLE OF CONTENTS........ 4
LIST OF ABREVIATIONS........ 7
CLINICAL TRIAL SUMMARY........ 8
GENERAL STUDY FLOW-CHART........ 18
1. INTRODUCTION AND RATIONALE........ 19
1.1 Study disease........ 19
1.1.1. Epidemiology of ovarian cancer........ 19
1.1.2. Standard first line treatment........ 19
B.4.3. Summary of the 1st line management for advanced disease........ 21
1.2 Background for Immunotherapy in ovarian carcinoma........ 21
1.2.1. OC is an immunogenic tumor: evidence........ 21
1.2.2.The PD-L1/PD1 immunosuppressive pathway in Ovarian Cancer........ 22
1.2.3 Rationale for combining pembrolizumab and standard chemotherapy........ 22
1.2.4 Rationale for combining pembrolizumab and bevacizumab........ 22
B.4. Informations on Pembrolizumab........ 23
1.3.1. Pharmaceutical and Therapeutic Background........ 23
1.3.2. Preclinical and Clinical Trial Data........ 24
1.3.4. Nonclinical Pharmacology........ 24
1.3.5. Nonclinical Pharmacokinetics........ 25
1.3.6 Safety Pharmacology/Toxicology........ 25
1.3.7 Clinical Development........ 26
1.4 Rationale........ 27
1.4.1 Rationale for the Trial and Selected Subject Population........ 27
1.4.2 Rationale for Dose Selection/Regimen/Modification for Pembrolizumab........ 27
1.4.3 Rationale for combination........ 28
2. INVESTIGATIONAL PLAN........ 29
2.1. Study Design........ 29
2.2. Study objectives and associated endpoints........ 30
2.3. Study Duration........ 31
3. STUDY POPULATION........ 31
3.1. Inclusion criteria........ 31
3.2. Exclusion criteria........ 32
4. STUDY TREATMENTS........ 33
4.1. Description........ 33
4.2. Treatment administration........ 34
4.3. Dose adaptation guidelines........ 36
4.3.1. Carboplatin/Paclitaxel........ 36
4.3.2. Bevacizumab........ 37
4.3.3. Pembrolizumab........ 38
4.4. Study treatments compliance and accountability........ 44
4.5. Concomitant medications and treatments........ 44
4.6. Treatment discontinuation........ 48

5. PATIENT ENROLMENT 49
6. ON-NEO-ADJUVANT-TREATMENT PERIOD ASSESSMENTS 50
7. END OF NEO-ADJUVANT-TREATMENT ASSESSMENTS 51
8. INTERVAL DEBLUKING SURGERY ASSESSMENTS 51
9. POST OPERATIVE ASSESSMENTS 51
10. ON-ADJUVANT-TREATMENT PERIOD ASSESSMENTS 52
11. END OF ADJUVANT TREATMENT ASSESSMENTS 52
12. POST CHEMOTHERAPY assessments 53
12.1. Patient without treatment maintenance (ARM A without bevacizumab) 53
12.2. Patient with treatment maintenance (ARM A with bevacizumab and ARM B) 53
13. EFFICACY EVALUATION 55
14. SAFETY EVALUATION / ADVERSE EVENTS 55
14.1. Definitions 55
14.2. Assessment of intensity 59
14.3. Reporting of Serious Adverse Event 59
14.4. Pregnancy notification 61
14.5. Overdose/misuse notification 61
14.6. Death notification 62
15. STATISTICAL CONSIDERATIONS 62
15.1. Determination of sample size 62
15.2. General considerations 62
15.3. Stopping rules 63
15.4. Statistical analysis 63
15.5. Interim analysis 63
15.6. Endpoint analysis 64
16. TRANSLATIONAL RESEARCH PROGRAM 68
17. STUDY COMMITTEES 70
18. DATA QUALITY CONTROL 71
19. MONITORING OF RESEARCH 71
20. AUDITS AND INSPECTIONS 72
21. ETHICAL, LEGISLATIVE AND REGULATORY CONSIDERATIONS 72
22. PROCESSING OF DATA AND STUDY DOCUMENTS ARCHIVING 74
23. CONIDENTIALITY OF TRIAL DOCUMENTS AND PATIENT RECORDS 75
24. OWNERSHIP OF DATA 75
25. PUBLICATIONS 75
26. REFERENCES 77
27. APPENDIX 80

Appendix 1 - ECOG performance Status *81*
Appendix 2 – FIGO staging 2014 .... *82*
Appendix 3 – Peritoneal Cancer Index (PCI) of residual tumor *83*
AND completeness of Cytoreduction Index (CCI) *83*
Appendix 4 – CLAVIEN-DINDO CLASSIFICATION *84*
Appendix 5 – Evaluation of disease upon RECIST 1.1 .... *85*
Appendix 6 – COMMON TERMINOLOGY CRITERIA FOR ADVERSE EVENTS V 4.03 (CTCAE) *89*

LIST OF ABREVIATIONS

**AE**	Adverse Event
**AESI**	Adverse Events of Special Interest
**aPPT**	activated Partial Thromboplastin Time
**AOC**	Advanced Ovarian carcinoma
**AUC**	Area Under the Curve
**BP**	Blood Pressure
**CCi**	Comprehensive Complication Index
**CCI**	Completeness of Cytoreduction Index
**CI**	Confidence interval
**CLB**	Centre Léon Bérard
**CR**	Complete response
**CNS**	central nervous system
**CT**	Chemotherapy
**CT-Scan**	Computed Tomography
**D**	Day
**DCR**	Disease Control Rate
**DLT**	Dose Limiting Toxicity
**DNA**	Deoxyribonucleic acid
**DSMB**	Data Safety Monitoring Board
**ECG**	Electrocardiogram
**ECI**	Event of Clinical Interest
**ECOG**	Eastern Cooperative Oncology Group
**eCRF**	Electronic Case Report Form
**EOC**	Epithelial Ovarian Cancer
**EOT**	End of Treatment
**FFPE**	Formalin-Fixed Paraffin-Embedded
**IDS**	Interval Debluking Surgery
**INR**	International Normalized Ratio
**IP**	Investigational Product
**ITT**	Intention To Treat
**IV**	Intravenous
**INN**	International Non-proprietary Name
**LLT**	Low Level Term
**MRI**	Magnetic Resonance Imaging
**ORR**	Overall response rate
**OS**	Overall survival
**PBMC**	Peripheral Blood Mononuclear Cell
**pCR**	Pathologic Complete Response
**PD**	Progressive disease
**PFS**	Progression free survival
**PK**	Pharmacokinetic
**PNN**	Poly Nuclear neutrophil
**PR**	Partial response
**PNN**	Poly Nuclear neutrophil
**PS**	Performance status
**PT**	Prothrombin Time
**SD**	Stable disease
**SmPC**	Summary of Product characteristics
**VEGF**	Vascular endothelial growth factor

**Study title**	A randomized, open-label, multicentric phase II trial of PEMBROLIZUMAB (Keytruda®) with chemotherapy versus chemotherapy alone (standard of care) as neo adjuvant treatment of ovarian cancer not amenable to front line debulking surgery		
**Study code**	NeoPembrOV: **NEO** adjuvant **Pembro**lizumab in **OVa**rian carcinoma		
**Sponsor**	ARCAGY-GINECO		
**EudraCT Number**	2016-004-163-39	**Sponsor ID**	GINECO-OV126b
**Coordinating Investigators**	Pr Isabelle RAY-COQUARD<br>Centre Léon Bérard, Lyon - FRANCE		
**Number of patients**	90	**Number of sites**	22
**Indication**	Advanced Ovarian carcinoma first line treatment		
**Study design**	Non-Comparative, open-label, randomized phase II trial.		
**Study rationale**	The standard procedure for initial diagnosis recommends the realization of laparoscopy first for all suspicious advanced ovarian carcinoma. This procedure should able to confirm histological diagnosis and to describe the all abdominal extension of the disease.<br>For advanced stages, complete primary cyto-reductive surgery followed by 6 cycles of chemotherapy based remains the standard of care as first treatment in ovarian cancer. It is part of a large surgery including total hysterectomy, bilateral salpingo-oophorectomy, omentectomy, appendectomy, lymphadenectomy and removal of all peritoneal carcinomatosis.<br>More recently, complete resection of all macroscopic disease at primary debulking surgery has been shown to be the single most important independent prognostic factor in advanced ovarian carcinoma [Du Bois Cancer 2009], and this was confirmed for interval debulking surgery (IDS) after neo adjuvant chemotherapy in the EORTC-GCG study (Vergote et al., 2010). These results suggest that neo-adjuvant chemotherapy followed by surgical cytoreduction is an acceptable management strategy for patients with advanced ovarian cancer and is more and more frequently used in Europe in advanced ovarian cancer patients with high burden of tumor (2012 French national guidelines (Saint Paul de Vence) & ESMO guidelines). Due to these confirmed results, the rate of patients receiving neo adjuvant chemotherapy increased over time compared to up front surgery (E Stoeckle et al 2014 and Luykx EJSO 2012)<br>Hence, we hypothesize that improving the response rate to neo adjuvant chemotherapy would improve the optimal debulking rate at IDS and ultimately the survival. This change of medical practices over time opened the possibility to explore new agent in combination with chemotherapy. For patients whose extent of disease is not amenable to complete or optimal upfront debulking surgery, neo adjuvant treatment with carboplatin plus paclitaxel should be considered, followed by an interval debulking surgery. A minimum of 3 cycles of carboplatin/paclitaxel must be administered before to propose interval surgery. After interval surgery, completion of the chemotherapy with 3 or 4 more chemotherapy regimen is proposed.<br>For patients with macroscopic residual disease or when disease remains unresectable, combination with bevacizumab to adjuvant chemotherapy then a maintenance phase of bevacizumab alone can be proposed as a standard of care (national guidelines Saint Paul de Vence www.arcagy.com).<br>Given that facts, there is a strong rationale to introduce additional neo adjuvant therapies that would strengthen the tumor shrinkage and improve the resectability rate.<br>Furthermore, immune surveillance plays an important role in tumor outcome of ovarian cancer patients. (Zhang L, et al. 2003). Indeed, clinical data in ovarian cancer patients have demonstrated that an antitumor immune response and immune evasion mechanisms are correlated, respectively, with a better and lower survival). Thus, immunotherapies are emerging as potential strategies to enhance classical EOC treatments (Lavoué, J transpl Med 2013).		

More recently, they also have demonstrated significant efficacy in aggressive cancers of other histology such as metastatic Lung Cancer (Brahmer et al., 2010), metastatic Renal Cell Cancer or metastatic Bladder Cancer (APOLO and Al, ASCO 2014).

Approximately half of OC patients display a spontaneous antitumor immune response by antibodies (Stone B et al 2003, Reuschenbach M, et al 2009) and oligoclonal T-cells (Schlienger et al , 2003) which recognize autologous tumor-associated antigens (TAAs). OC exhibits an extreme degree of heterogeneity of TAAs with an average of 60 private nonsynonymous mutations per tumor which are rarely shared among different tumors.

Though data remains scarce, high IHC PD-L1 expression (score 2 & 3) has been detected in 68% of ovarian cancer patients (n=70) and that expression of PD-L1 had a strong prognostic value (Hamanashi J, 2007). The authors found also that the density of intraepithelial CD8+ T cells was inversely correlated to expression of PD-L1 by tumors, suggesting that the expression of PD-L1 on tumor cells may inhibit invasion of tumor epithelium by CD8+ T cells.

In addition, PD-1 expression at the surface of intra-tumoral CD4+ FOXP3+ Tregs was found to show the highest levels in ovarian cancer (around 20% of the cells) compared to other tumor types, including melanoma, renal cell cancer or hepatoma (Kryczek et al., 2009). Thus targeting PD-1/PD-L1 pathway may inhibit Treg expression, one of the major component of ovarian cancer immunosuppression. Also Curiel et al showed that myeloid dendritic cells (MDCs) from ovarian cancer express PD-1 and that blockade of PD-1 enhanced MDC-mediated T-cell activation, including upregulation of IL-2 and interferon-gamma, and down regulation of IL-10, which resulted in enhanced T-cell immunity against autologous ovarian human tumors into NOD-SCID mice. (Curiel et al, 2003).

Together with the aforementioned data on immune infiltration, these data provide the rationale for a therapeutic PD-1/PD-L1 pathway blockade in ovarian cancer.

In ovarian carcinoma patients, the anti-PD1 compound nivolumab has been reported to achieve 3 objectives responses out of 13 (23%) heavily pre-treated patients (Hamanishi J, ASCO 2014). Response was prolonged over 1 year in 2 out of the 3 responders (Hamanishi J, ASCO 2015). Similarly, the anti-PD1 pembrozilumab achieved 3 confirmed responses (11.5% [(95% CI, 2.4-30.2]) in 26 patients treated in a phase IB study and 3 additional patients had a tumor reduction of at least 30%. Most common AEs were fatigue (42.3%), anemia (30.8%), and decreased appetite (30.8%). Drug-related AEs occurred in 69.2% of pts (grade ≥ 3, 1/26 pts) (Varga A et al, 2015).

The anti-PD-L1 avelumab has reported a 10.7% objective response and a 44% stabilization rate in 75 patients with ovarian cancer in relapse (Disis M et al, 2015). In this study, confirmed or unconfirmed responses (n=11) tend to be more frequently observed in patients with low burden of tumor, limited number of prior lines of chemotherapy and in the setting of platinum-sensitivity. Toxicity was minimal. Considering all grades, fatigue was observed in 16% of the patients, chills in 12%, nausea in 10.7%, diarrhea in 10.7%, rash in 8% and hypothyroidism in 5.3%.

Rationale for combining pembrolizumab and standard chemotherapy

Kryczek et al compared the PD-1 expression level at the surface of intra-tumoral CD4+ FOXP3+ Tregs among many cancer types. Interestingly, the higher level of PD-1 expression (around 20%) was found on Tregs of ovarian cancers whereas it was much lower (<10%) in other cancer types (Colon cancer, Hepatic cancer, Melanoma, Pancreatic carcinoma, Renal cell carcinoma) (58). PD-L1 expression has also been detected in ovarian cancer tissue analysis by Immunohistochemistry staining and its level of expression has been correlated to a bad outcome of patients (59). Together with the aforementioned data on immune infiltration, these results provide rationale for a therapeutic PD-1/PD-L1 pathway blockade in ovarian cancer. In the published trials on such compounds, addition of pembrolizumab to chemotherapy or using alone has been shown to improve the response rates with a median time to response at 8 weeks (60,61).

Rationale for combining pembrolizumab and bevacizumab

There are several data suggesting that pembrolizumab and bevacizumab may be synergistic. Enhanced tumor angiogenesis is commonly associated with absence of tumor-infiltrating T

	cells in patients (Bouma-ter Steege JC et al, 2004). There is evidence in OC that tumor expression of VEGF is negatively correlated to the density of CD3+TILs (Zhang L et al 2003) and this phenotype is associated with early recurrence, consistent with prior studies showing a correlation of VEGF to early recurrence and short survival. Furthermore, in ascites, high levels of VEGF correlate to low numbers of NK T-like CD3+CD56+ cells (Bamias et al, 2008).<br><br>This randomized phase II study aims to evaluate the efficacy of pembrolizumab in combination with the standard neo adjuvant chemotherapy followed by IDS and the safety of this strategy in patients with advanced ovarian cancer. We assume that its administration in the neo adjuvant setting combination with standard of care (4 cycles of standard chemotherapy) would improve the response rate and consequently will help to achieve optimal debulking rate at IDS.<br><br>After surgery, patients will continue to be treated with standard of care (chemotherapy for 2 to 5 cycles plus or less bevacizumab) or the same combination plus pembrolizumab (keytruda).
**Study objectives and associated end-points**	**PRIMARY OBJECTIVE**<br>• The primary objective is **to evaluate the efficacy** of neoadjuvant pembrolizumab and chemotherapy or chemotherapy alone measured by the complete resection rate (CC0) after interval debulking surgery evaluated by a central medical review. Complete resection will be defined as the removal of all macroscopic residual tumor (Complete Cytoreduction score = 0).<br>**SECONDARY OBJECTIVES**<br>• **To evaluate the efficacy** of the addition of pembrolizumab to chemotherapy assessed by:<br>o CCI score evaluated by centers and by central review<br>o The PCI score evaluated centers and by central review<br>o The Objective Response Rate at the time of interval debulking surgery (after 4 neo adjuvant cycles) using RECIST 1.1 criteria<br>o The Best Overall Response to the global strategy of interval debulking surgery + chemotherapy +/- Pembrolizumab assessed by CT-Scan (using RECIST 1.1) at the end of treatment visit & before interval surgery.<br>o The rate of pCR evaluated by central review (pathologist)<br>o The Progression-Free Survival (PFS) using RECIST 1.1 criteria<br>o Biological Progression-Free Interval (PFIBIO), by serum Ca125 measured according to the GCIG criteriaThe overall survival<br>• **To assess the safety** profile of neo adjuvant and adjuvant pembrolizumab when combined with standard of care, according to NCI CTC-AE v4.03.<br>• **To assess the** Post-operative mortality<br>• **To assess the** Post-operative morbidity according to modified Clavien Dindo scoring.<br>**EXPLORATORY OBJECTIVES**<br>• To study the difference between CCI and PCI score evaluated by centers and CCI and PCI score evaluated by the central medical review.<br><br>**TRANSLATIONNAL RESEARCH OBJECTIVES**<br>o To improve the understanding of mechanism of action of pembrolizumab and resistance mechanisms to immune therapy<br>o To identify biomarkers predicting efficacy (CC0, PFS, RR, pCR)
	**INCLUSION CRITERIA**<br>1. Be willing and able to provide written informed consent for the trial.<br>2. Woman ≥ 18 and ≤ 75 years old on day of signing informed consent<br>3. Histologically confirmed diagnosis of epithelial ovarian carcinoma or fallopian tube carcinoma or primary peritoneal carcinoma with the exception of clear cell, mucinous histology. Histology should be obtained by laparoscopy (or by laparotomy).<br>4. High grade serous or endometrioid (see appendix 1 bis)

Study population	

Study population

5. Advanced FIGO stage IIIC to IV patient not able to receive primary debulking surgery for which neo adjuvant chemotherapy with carboplatin and paclitaxel is recommended (primary debulking surgery has been denied after an evaluation through laparoscopy or laparotomy). Patients with extra abdominal metastasis (FIGO 2014 Stage IV) can be included in case of completely resecable metastasis.
6. Primary debulking surgery denied and maximum surgical effort of cytoreduction with the goal of no residual disease planned at interval debulking surgery. Sugarbaker index before inclusion must be less than 30
7. Eligible for carboplatin and paclitaxel chemotherapy in accordance with local standards of care following cytoreductive surgery.
8. Debulking surgery anticipated in a center with excellence.
9. ECOG performance status (PS) ≤ 2.
10. Life expectancy of at least 6 months,
11. Interval between diagnosis and enrolment (informed consent) ≤ 8 weeks,
12. Be willing to provide blood, and tissue from a newly obtained core or excisional biopsy of a tumor lesion. *Newly-obtained is defined as a specimen obtained up to 8 weeks (56 days) prior to initiation of treatment on Day 1.*
13. Demonstrate adequate organ function as defined, all screening labs should be performed within 7 days before randomization.
14. Adequate hematological laboratory value:

- Absolute neutrophil count (ANC) : ≥1,500/mm^3
- Platelets : ≥100,000/mm^3

15. Hemoglobin : ≥9 g/ Adequate renal laboratory value:

    Serum creatinine **AND** Measured or calculated creatinine clearance [a] (GFR can also be used in place of creatinine or CrCl) ≤1.5 X upper limit of normal (ULN) **OR** ≥60 mL/min for subject with creatinine levels > 1.5 X institutional ULN
    (Creatinine clearance is calculated according to Cockcroft formula or to MDRD formula for patients older than 65 years-old. Glomerular filtration rate or creatinine clearance according to MDRD formula is: GFR = 186 x (creatinine (µmol/l) x 0,0113)-1,154 x age-0,203 x 0.742.)

16. Adequate hepatic laboratory value:
    - Serum total bilirubin: ≤ 1.5 X ULN **OR**
    - Direct bilirubin: ≤ ULN for subjects with total bilirubin levels > 1.5 ULN
    - LDH, CRP
    - AST (SGOT) and ALT (SGPT): ≤ 2.5 X ULN OR ≤ 5 X ULN for subjects with liver metastases
17. Adequate coagulation laboratory value:
    - International Normalized Ratio (INR) or Prothrombin Time (PT) : ≤1.5 X ULN unless subject is receiving anticoagulant therapy as long as PT or PTT is within therapeutic range of intended use of anticoagulants
    - Activated Partial Thromboplastin Time (aPTT): ≤1.5 X ULN unless subject is receiving anticoagulant therapy as long as PT or PTT is within therapeutic range of intended use of anticoagulants
18. Female subject of childbearing potential should have a negative urine or serum pregnancy within 72 hours prior randomization. If the urine test is positive or cannot be confirmed as negative, a serum pregnancy test will be required.
19. Female subjects of childbearing potential should be willing to use 2 methods of birth control or be surgically sterile, or abstain from heterosexual activity for the course of the study through 4 months after the last dose of study medication and six months after the last dose of bevacizumab, or paclitaxel, or carboplatin. Subjects of childbearing potential are those who have not been surgically sterilized or have not been free from menses for > 1 year. (see the contraception requirement)

20. Patient should be beneficiary of healthcare coverage under the social security system.

EXCLUSION CRITERIA

1. Histological diagnosis of malignant tumor of non-epithelial origin (e.g. germ cell tumor, sex cord-stromal tumor) of the ovary, the fallopian tube or peritoneum or borderline tumor of the ovary (tumor of low malignant potential).
2. Patients with extra abdominal metastasis (FIGO 2014 Stage IV) not completely resectable, as e.g. multiple parenchymal lung metastases (preferably histologically proven), non resectable lymph node metastases, brain metastases.
3. Prior systemic therapy for ovarian cancer (e.g. chemotherapy, monoclonal antibody therapy, oral targeted therapy, hormonal therapy),
4. Prior radiotherapy to the abdomen or prior radiotherapy to an extra-abdominal target volume that would bear the risk of increased toxicity of chemotherapy,
5. Serious illness or concomitant non-oncological disease such as neurologic, cardiologic (ie. Congestive heart failure > NYHA II), psychiatric or infectious disease, active ulcers (gastrointestinal tract, skin) or a laboratory abnormality that may Increase the risk associated with study participation or study drug administration and in the judgment of the investigator would make the patient inappropriate for entry into the study,
6. Any contraindications for therapy with paclitaxel or carboplatin, e.g. a history of severe hypersensitivity reactions to paclitaxel or platinum-containing compounds and their excipients, or other drugs formulated with Polyoxyl 35Is currently participating and receiving study therapy or has participated in a study of an investigational agent and received study therapy or used an investigational device within 4 weeks of the first dose of treatment.
7. Diagnosis of immunodeficiency or receiving prolonged period of systemic steroid therapy or any other form of immunosuppressive therapy within 7 days prior to the first dose of trial treatment.
8. Known history of active Bacillus Tuberculosis (TB)
9. Hypersensitivity to pembrolizumab or any of its excipients.
10. Prior anti-cancer monoclonal antibody (mAb) within 4 weeks prior to study Day 1 or not recovery (i.e., ≤ Grade 1 or at baseline) from adverse events due to agents administered more than 4 weeks earlier.
11. Known additional malignancy that is progressing or requires active treatment. Exceptions include basal cell carcinoma of the skin or squamous cell carcinoma of the skin that has undergone potentially curative therapy or in situ cervical cancer.
12. Known active central nervous system (CNS) metastases and/or carcinomatous meningitis. Subjects with previously treated brain metastases may participate provided they are stable (without evidence of progression by imaging for at least four weeks prior to the first dose of trial treatment and any neurologic symptoms have returned to baseline), have no evidence of new or enlarging brain metastases, and are not using steroids for at least 7 days prior to trial treatment. This exception does not include carcinomatous meningitis which is excluded regardless of clinical stability.
13. Active autoimmune disease that has required systemic treatment in the past 2 years (i.e. with use of disease modifying agents, corticosteroids or immunosuppressive drugs). Replacement therapy (e.g., thyroxine, insulin, or physiologic corticosteroid replacement therapy for adrenal or pituitary insufficiency, etc.) is not considered a form of systemic treatment.
14. Prior therapy with an anti-PD-1, anti-PD-L1, or anti-PD-L2 agent.

15. History of Human Immunodeficiency Virus (HIV) (HIV 1/2 antibodies).
16. Active Hepatitis B (e.g., HbsAg reactive) or Hepatitis C (e.g., HCV RNA [qualitative] is detected).
17. Vaccination with a live vaccine within 30 days of planned start of study therapy.

    Note: Seasonal influenza vaccines for injection are generally inactivated flu vaccines and are allowed; however intranasal influenza vaccines (e.g., Flu-Mist®) are live attenuated vaccines, and are not allowed.
18. History of (non-infectious) pneumonitis that required steroids or current pneumonitis.
19. Active infection requiring systemic therapy.
20. History or current evidence of any condition, therapy, or laboratory abnormality that might confound the results of the trial, interfere with the subject's participation for the full duration of the trial, or is not in the best interest of the subject to participate, in the opinion of the treating investigator.
21. Psychological, familial, sociological or geographical factors potentially hampering compliance with the study protocol and follow-up schedule,
22. Psychiatric or substance abuse disorders that would interfere with cooperation with the requirements of the trial.
23. Active alcohol or drug abuse,
24. Pregnant or breastfeeding, or expecting to conceive or father children within the projected duration of the trial, starting with the pre-screening or screening visit through 4 months after the last dose of trial treatment, and six months after the last dose of bevacizumab, or paclitaxel, or carboplatin
25. Patient unable to give their consent by their own (guardianship and curatorship)

Experimental plan

Following inclusion, patients will be randomized to one of the 2 study arms (ratio 2:1):

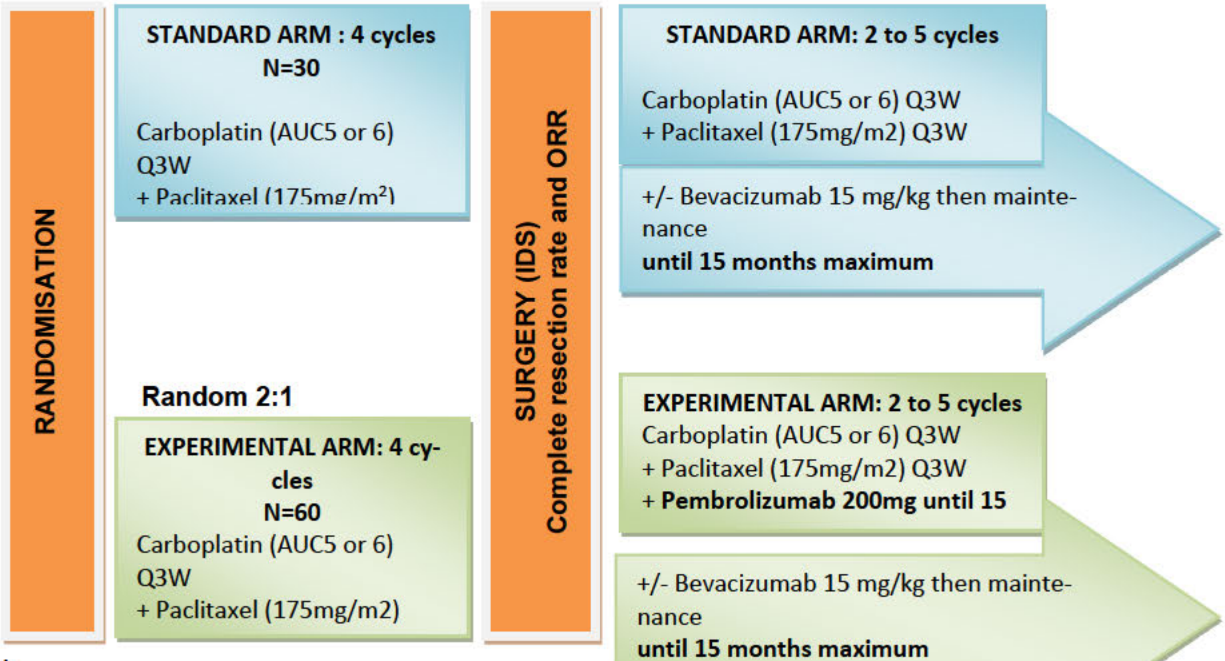

[A] STRATIFICATION FACTOR:

- Centers
- FIGO stage (IIIC versus IV)
- Metastasis volume (< 5cm versus ≥ 5 cm
- Planned used of Bevacizumab after IDS (either arm A or B) (yes or no)

*BEVACIZUMAB MAY BE INTRODUCED IN BOTH ARMS AFTER INTERVAL SURGERY AS STANDARD OF CARE

Neo-Adjuvant therapy

- Arm A (n=30): 4 neo-adjuvant cycles of standard 3 weekly Carboplatin/Paclitaxel
- Arm B (n=60): 4 neo-adjuvant cycles of standard 3 weekly Carboplatin/Paclitaxel combined with pembrolizumab

Arm A: **carboplatin (AUC5 or 6) and paclitaxel (175mg/m²)**, q3 weeks.
Arm B: **Pembrolizumab 200 mg then carboplatin (AUC 5 or 6) and paclitaxel (175mg/m²)**, q3 weeks.

Interval debulking surgery (IDS)

A mandatory laparoscopy with resectability evaluation should be performed 4 weeks +/- 2 week after the last (4th cycle) chemotherapy +/- experimental drug administration with the aim to perform IDS. Sugarbaker Index will be assessed during laparoscopy.
If IDS is postponed (unresectable disease), multiple biopsies through laparoscopy will be required.
If laparoscopy could not be performed after 4 cycles, due to other reason than progression, a contact with the Trial Manager will be required.

Adjuvant therapy

Adjuvant therapy will start after surgical wound healing and under no circumstances the treatment will start before 4 weeks after surgery. It optimally should start not beyond 6 weeks after surgery.
The administration of Bevacizumab is at the investigator's discretion.

- Arm A: 2 cycles of **Carboplatin (AUC 5 or 6) and paclitaxel (175mg/m²)**, q3 weeks +/- **bevacizumab** (15 mg/kg Q3W) until 15 months in total
- Arm B: **Pembrolizumab 200 mg** plus 2 cycles of **carboplatin (AUC 5 or 6) and paclitaxel (175mg/m²)**, +/- **bevacizumab** (15 mg/kg Q3W) – on Day 1 of each 3 weeks cycle until 15 months in total.

The total number of cycles of chemotherapy will be 6 cycles from the start of the neo-adjuvant chemotherapy. Three adjuvant additional cycles are allowed if required (maximum 9 cycles).

Maintenance therapy

After chemotherapy:

- In the Arm A & B, only for patients receiving bevacizumab (as standard therapy) (15 mg/kg Q3W), this will be continue until 15 months in total.
- In the arm B, patient will receive Pembrolizumab 200 mg until 15 months in total plus for patients receiving bevacizumab (as standard therapy) (15 mg/kg Q3W), also until 15 months in total.
  The maximum duration of the study will be 24 months treatment (from the randomization to the last dose of experimental product).

Pembrolizumab

Pembrolizumab is a humanized antibody. It targets the programmed cell death 1 (PD-1) receptor

Pembrolizumab 50mg is a lyophilized Powder for Injection

Pembrolizumab 100mg is a liquid solution for Injection

Both dosage forms are stored under refrigerated conditions (2° to 8°C).

**Study treatment**	**Supportive Care and general prophylaxis**<br>Patients should receive full supportive care, including transfusions of blood and blood products, antibiotics, anti-emetics, etc., when appropriate.<br><br>Systemic corticosteroids may attenuate potential beneficial immunologic effects of treatment with pembrolizumab but may be administered at the discretion of the treating physician. If feasible, alternatives to corticosteroids should be considered.<br><br>Initiation of granulocyte colony-stimulating factors to prevent neutropenia is prohibited at Cycle 1. Administration at Cycle ≥ 2 is possible at the discretion of the treating physician. If feasible, alternatives should be considered (decrease of chemotherapy dose)<br><br>**Prohibited concomitant medications**<br>Subjects are prohibited from receiving the following therapies during the screening and treatment phase (including retreatment for post-complete response relapse) of this trial:<br>• Immunotherapy not specified in this protocol<br>• Chemotherapy not specified in this protocol<br>• Investigational agents other than pembrolizumab<br>• Radiation therapy<br>• Live vaccines within 30 days prior to the first dose of trial treatment and while participating in the trial.<br><br>Subjects who, in the assessment by the investigator, require the use of any of the aforementioned treatments for clinical management should be removed from the trial.
**Translational research**	The objective is to improve the understanding of mechanism of action of pembrolizumab and resistance mechanisms Tumor Tissue Collection and Correlative Studies Blood Sampling will be done:<br>• Mandatory biological specimens: Formalin-Fixed Paraffin-Embedded (FFPE) tumor biopsies at inclusion and surgical tumor specimen following neo-adjuvant treatment<br>• Mandatory plasma/serum and whole blood before and on treatment<br>• Mandatory frozen Peripheral Blood Mononuclear Cell (PBMC) before and on treatment<br>• Optional biological specimens: frozen tumor biopsies at inclusion and surgical tumor specimen following neo-adjuvant treatment.<br>• Optional biological specimens (blood and tumor) at progression<br>• Optional ascitis collection when available
	**Sample size determination**<br>The sample size calculation in the experimental arm is based on the Fleming's A'Hern single-stage design. In our study, 54 evaluable patients are required in the Pembrolizumab plus chemotherapy group. More patients will be included in the experimental arm justifying the choice of a 1: 2 randomization rate. The control group will include 27 patients.<br>Assuming that 10% of patients will be lost to follow-up or non-evaluable for the primary criteria, a total of 90 patients will be included and randomized (60 in the experimental arm versus 30 in the control arm).Thirty-three successes (CC0) over the 54 evaluable patients in the experimental arm are expected to declare the experimental treatment as promising. No formal comparison between the experimental arm and the control arm will be performed.<br>**Stratification**<br>• Centre / The FIGO-stage: IIIC versus IV / Metastases volume (< 5cm; ≥ 5cm)<br>• Planned used of Bevacizumab after IDS (either arm A or B) (yes or no)

<table>
<tr>
<td>Statistical considerations</td>
<td>
The main analysis of efficacy criteria will be done on the ITT population.
Safety analysis
Safety analyses are planned :

After the randomization of 10 patients treated with cycles with the combination of bevacizumab plus pembrolizumab (after 4 cycles)
After the randomization of 20 patients in the experimental arm once these patients have received 4 neo adjuvant treatment cycles.

- Sequentially for the subgroup of patients having completed 2 consecutive treatment cycles with the combination of bevacizumab plus pembrolizumab.
Safety analysis data will be assessed by the DSMB.
Primary endpoint analysis
The number of complete debulking score CC0 (success), evaluated by a central medical review, will be presented in both arms. Primary endpoint will be analyzed on the ITT population. If surgery could not be performed due to progression disease before the end of the neo adjuvant period, or due to insufficient efficacy of the neo adjuvant therapy, patient will be considered as failure. If surgery could not be performed due to other reason, patients will be considered as non-evaluable.
A sensitivity analysis will be performed to measure the complete resection rate using the CCI score evaluated by centers.
Secondary endpoint analysis
1/ CCI score evaluated by centers and by central review:
2/ PCI score evaluated by centers and by central review:
3/ Overall Response rate (OR):
4/ Best response
5/ Pathological complete response (pCR) evaluated by central review (pathologist)
6/ / Progression Free Survival (PFS):
7/Biologic Progression Free Interval (PFIbio):
8/ Overall survival
9/ Safety
10/ Post-operative mortality
11/ Post-operative morbidity
Exploratory analysis
Concordance between CCI and PCI score evaluated by the center and by the central medical review.
Translational research using blood and tumor samples to identify responders and resistant patients to IO's (independent specific SAP)
</td>
</tr>
<tr>
<td>Study Committees</td>
<td>
Steering Committee
A steering committee will be regularly informed of the accrual rate of inclusion and of any emergent problems and will review the activity and safety data at the end of the study.
The <u>Independent Data safety monitoring board</u> (iDSMB) will be composed of statisticians and two medical experts in the field of ovarian cancer.
This committee will have to:
- Meet after 10 patients treated with cycles with the combination of bevacizumab plus pembrolizumab (after 4 cycles
</td>
</tr>
</table>

	- Meet after the 20th included patient in the experimental arm once these patients have received 4 neo adjuvant treatment cycles to evaluate the safety.<br>- Sequentially for the subgroup of patients having completed 2 consecutive treatment cycles with the combination of bevacizumab plus pembrolizumab to evaluate the safety.<br>- Review safety Data and make an assessment of the safety profile (the benefit risk ratio)<br>- Review each event that could modify the benefit risk ratio of the study (i.e. scientifics, safety, ethics events).<br>Additional meetings may be called at any time if an event occurs.<br><br>The Centralized review Committee will be composed of three experts/investigators involved in the study: the coordinating investigator and two surgeons.<br>The members will meet in person or via teleconference to review the anonymized operative and pathological reports at screening, at the Interval Debulking Surgery and Other Debulking Surgery of all patients. The aim of this review is to reach a consensus on the data to extract from reports.
**Study calendar**	• First patient in : Q3 2017<br>• Last patient randomized: Q1 2019<br>• End of treatment: Q1 2021<br>• End of follow up period: Q1 2025

GENERAL STUDY FLOW-CHART

Trial period	Screening	R A N D O M I Z A T I O N	Neo-adjuvant<br>Started within 14 days after randomization					S U R G E R Y[8]	Post-op visit<br>*(4 weeks ± 15 days after surgery)*	Adjuvant						Maintenance (if any)				Post ttt observation<br>*(Q3m the first year and q6m during 3 years)*
**Treatment cycle/Title**	**Inclusion**		**C1**	**C2**	**C3**	**C4**	**End of neoadjuvant Visit[d]**			**C5[10]**	**C6**	**C7**	**C8**	**C9**	**End of adjuvant visit**<br>*(3 to 4 weeks after last chemo adm.)*	**Until15 months in total or until progression or toxicity**			**End of Ttt visit**<br>*(4 weeks after last ttt adm.)*	
**Timelines**			D-3 to D1	D-3 to D1	D-3 to D1	D-3 to D1				D-3 to D1	D-3 to D1	D-3 to D1	D-3 to D1	D-3 to D1		**q3w**	**q3m**	**q6m**		
Informed consent	X[b]																			
Informed consent biologic study	X[b]																			
Inclusion / Non-inclusion criteria	X[b]																			
Demographic/Medical history	X[b]																			
Physical examination (Height, weight, ECOG (PS), blood pressure)	X[b]		X	X	X	X	X		X	X	X	X	X	X	X	X	X		X	X
Prior/ change in concomitant medication review	X[b]		X	X	X	X	X			X	X	X	X	X	X	X			X	
Hematology[1]	X[a]		X	X	X	X	X			X	X	X	X	X	X	X[11]	X		X	
Chemistry[2]	X[a]		X	X	X	X	X			X	X	X	X	X	X	X	X		X	
Hemostasis (INR and aPTT)	X[a]		X	X	X	X	X			X	X	X	X	X	X	X[11]	X		X	
CA-125	X[a]		X	X	X	X	X			X	X	X	X	X	X		X		X	X
Pregnancy test[3]	X[a]		X	X	X	X	X			X	X	X	X	X						
ECG[4]	X[b]																			
CT scan[5] (Thoracic-Abdominal-Pelvic) or MRI	X[b]				X		X								X		X[12]	X	X	X[13]
Trial treatment administration			X	X	X	X				X	X	X	X	X		X	X	X		
Adverse events/complications treatment			X	X	X	X	X			X	X	X	X	X	X	X	X		X	X
Sugarbaker index + targets	X[b]							X[9]												
Complications/AE of special interest									X											
Blood sample	X			X[6]		X[6]														X[6]
Tumor block (FFPE)	X							X[7]												X[7]
Tumor tissue (frozen)								X												
Ascitis (when available)	X					X		X												
Patient status																				X

a: within 7 days prior to randomization, **b:** within 28 days prior to first treatment administration, **c:** Optional; **d:** Visite 3 weeks +/-3 days after last treatment administration

1: hematology : Hemoglogin, Platelet Count, WBC (total and differential), Absolut Neutrophils Count, platelets

2: Chemistry: Albumin and LDH (only at screening), creatinin, creatinin clearance ALT, AST, PAL, Total bilirubin, urine analyse for bevacizumab patients, and Hormonology for pembrolizumab patients: (TSH, T3 and T4)

3: If applicable, perform on women of childbearing potential only. If urine pregnancy results cannot be confirmed as negative, a serum pregnancy test will be required.

4: ECG at screening only. To be repeated if clinically indicated

5: Thoracic-abdominal-pelvic CT-scan is recommended. However, if it is not feasible, MRI of the abdomen and pelvis and a chest X-ray are acceptable, but subsequently the same imaging technique should be employed for routine imaging assessments. As isolated pulmonary metastases are rare in ovarian cancer, chest imaging will only be repeated as clinically indicated.

6: D1C2 and end of C4 administration; at progression

7: Collection of tumor samples from ovary metastasis, at baseline, IDS and relapse (optional)

8: Interval surgery debulking 3 to 4 weeks after the last chemotherapy administration

9: PCI before and after debulking surgery. CCI after surgery

10: Adjuvant therapy will start after complete surgical wound healing. Under no circumstances, the treatment will start before 4 weeks after surgery and optimally should not be beyond 6 weeks after surgery.

11: Test to be repeated if clinically indicated

12: To be performed if required

13: Every 6 months during the first year, then, at the time of suspected progression, when required according to investigator

1. INTRODUCTION AND RATIONALE

1.1 Study disease

1.1.1. Epidemiology of ovarian cancer

Ovarian cancer (OC) is the fifth most common cause of death from cancer in women (1). In the European Community, approximately 28,000 new cases of ovarian cancer and approximately 17,000 deaths are reported annually, ranking ovarian cancer as the leading cause of death from gynecological cancer. The incidence of OC increases with age and is most prevalent in the eighth decade of life. More than 70% of the patients are diagnosed with advanced disease and less than 40% of women with OC are cured (2). Currently, the five-year survival is close to 80-90% for stage I of 50-60% for stage II, 30% for stage III and 10% for stage IV. The 5-years survival, all stages combined, is approximately 45%. Ovarian cancer is often asymptomatic in its early stages and the majority of epithelial ovarian cancers remain clinically undetected until patients have developed late stage disease (3). As the majority (75%) of ovarian cancer patients is diagnosed with advanced disease and despite high response rates with surgery followed by chemotherapy, 75% of women ultimately die from disease progression. Once stage III and IV ovarian cancer is diagnosed, the five-year survival decreases from to approximately 20-25%.

1.1.2. Standard first line treatment

1.1.2.1 Surgery and place of interval debulking

The standard procedure for initial diagnosis established in the French national guidelines (Saint Paul de Vence) recommend the realization, after CT scan, of laparoscopy first for all suspicious advanced ovarian carcinoma. This is able to confirm histological diagnosis and to describe the all abdominal extension of the disease.
For advanced stages, complete primary cyto-reductive surgery followed by 6 cycles of chemotherapy based remains the standard of care as first treatment in ovarian cancer (4).
It is part of a large surgery including total hysterectomy, bilateral salpingo-oophorectomy, omentectomy, appendectomy and lymphadenectomy and removal of all peritoneal carcinomatosis.

More recently, complete resection of all macroscopic disease at primary debulking surgery has been shown to be the single most important independent prognostic factor in advanced ovarian carcinoma (5) and this was confirmed for interval debulking surgery after neo adjuvant chemotherapy in the EORTC-GCG study (6). Based on these findings there was an agreement on the GCIG consensus conference that “optimal” cytoreduction should no longer be defined as residual tumor < 1 or < 0.5 cm, but as a resection without macroscopic residual tumor (7).

Vergote et al. reported the results of a randomized trial conducted by the EORTC-GCG in 670 patients with advanced ovarian carcinoma (6). The authors compared primary debulking surgery followed by six courses of platinum-based chemotherapy (n=336) versus three or 4 courses of platinum-based neo adjuvant chemotherapy followed by IDS and an additional three courses of platinum-based chemotherapy (n=334). No residual disease is reported for 20% of patients after primary debulking and for 51% of patients after IDS. The median overall survival was not significantly different between groups (29 months with primary debulking surgery vs. 30 months with neo adjuvant chemotherapy). However, postoperative rates of adverse effects and mortality tended to be higher after primary debulking surgery than after IDS. These results are consistent with the conclusions of a recent meta-analysis of 21 non-randomized trials (8). Therefore, neo adjuvant chemotherapy is consider as not inferior to primary debulking surgery for patients with stage IIIC or IV ovarian cancer. Together all these studies show that only patients with proven stage IIIC or IV and non-optimally resectable should be considered for neo adjuvant chemotherapy.
Neo adjuvant chemotherapy represents an alternative strategy for patients with stage IIIC or IV who are felt to be optimally (no macroscopic residual tumor) unresectable with upfront surgery (9, 10, 11). Chemotherapy followed by interval debulking result in fewer and simpler operations and lesser morbidity for the patients involving less inconvenience and toxicity for the patient with equivalent survival outcomes (12). Vergot et al. data suggest that neo-adjuvant chemotherapy followed by surgical cytoreduction is an acceptable management strategy for patients with advanced ovarian cancer (13) and is more and more frequently used in Europe in advanced ovarian cancer patients with high burden of tumor (Saint Paul de Vence guidelines; www.arcagy.org).

Due to these results, confirmed by other clinical trial (CHORUS trial), since 5 years, the rate of patients receiving neo adjuvant chemotherapy increased over time compared to up front surgery (14). We recently reported more

than 65 to 70% of patients with advanced ovarian cancer received neo adjuvant chemotherapy prior to interval surgery in 2013 (15, 16).

One major point, from the recent EORTC-GGC trial, are the strongest independent predictors of prolonged survival included (i) the absence of residual tumor after surgery and (ii) the small tumor size (< 5cm vs > 5 cm) before randomization (6). Hence, we hypothesize that improving the response rate to neo adjuvant chemotherapy would improve the optimal debulking rate at IDS and ultimately the survival.

This change of medical practices over time opened the possibility to explore new agent in combination with chemotherapy where tumor samples will be available before any treatment and at the time of the interval surgery. More specifically, all agents able to improve response rate to chemotherapy and rate of complete resection of the disease with the interval surgery could be interesting to test in such setting. Given that facts, there is a strong rationale to introduce additional neo adjuvant therapies that would strengthen the tumor shrinkage and improve the resectability rate.

1.1.2.2. Chemotherapy

Since 1996, platinum and paclitaxel combination therapy has become the standard-of-care first-line chemotherapy regimen (17). Worldwide, the use of carboplatin has replaced that of cisplatin because of carboplatin's superior tolerability profile together with equal effectiveness. However, the success of this approach is limited and approximately 70% of patients fail to achieve complete responses, or eventually relapse. Therefore, despite all the new treatment options, only a minority of women with Advanced Ovarian Carcinoma (AOC) can be cured.

1.1.1.1.1 Paclitaxel (non-investigational drug)

Paclitaxel is an anti-microtubule agent that is approved for the treatment of various solid tumors, including first and second-line treatment of ovarian cancer. Paclitaxel interferes with the normal dynamic reorganization of the microtubule network that is essential for vital interphase and mitotic cellular functions. Treatment with paclitaxel may be associated with myelosuppression, hypersensitivity reactions, myalgia and arthralgia, peripheral motor neuropathy and neurosensory symptoms (such as paresthesia, dysesthesia), asthenia, gastrointestinal adverse events (such as nausea, vomiting, diarrhea), cardiovascular side effects and infusion site reactions. In this trial, Paclitaxel shall be administered at a dose of 175 mg/m2 over 3 hours as recommended by the manufacturer in patients with ovarian cancer in combination with carboplatin. Dosing is repeated every 21 days.

1.1.1.1.2 Carboplatin (non-investigational drug)

Carboplatin is approved for the treatment of advanced epithelial ovarian cancer. It binds to DesoxyriboNucleotid Acid (DNA) to produce interstrand DNA crosslinks which modify structure and inhibit DNA synthesis. Treatment with carboplatin may be associated with myelosuppression, gastrointestinal adverse events (such as nausea, vomiting, constipation or diarrhea), transient elevation of liver function tests, nephrotoxicity, electrolyte disturbances, ototoxicity, neurotoxicity and allergic reactions. Based on the Calvert formula, a target AUC of 4-7.5 mg/mL min provides an appropriate dose range for combination therapy. In this trial, the recommended carboplatin dose is based on a target Area Under the Curve (AUC) of 5 g/mL·min.

1.1.1.1.3 The use of bevacizumab in ovarian cancer 1st line

Bevacizumab is a monoclonal antibody specifically designed to bind and inhibit circulating VEGF. The assessment of safety and efficacy of bevacizumab added to standard chemotherapy in first-line treatment following optimal surgical debulking was derived from the 2 phase III trials, GOG0218 (18) and ICON7 (19). These trials were conducted in women with newly diagnosed OC after up front surgery and demonstrated that front-line bevacizumab in combination with standard chemotherapy (carboplatin and paclitaxel), followed by the continued use of bevacizumab alone, significantly increased progression free survival (PFS) compared to chemotherapy alone.
Since 2011, bevacizumab has been approved in Europe in combination with carboplatin and paclitaxel and indicated for the front-line treatment of adult patients with advanced (International Federation of Gynecology and Obstetrics

[FIGO 1988] stages III B, III C and IV) epithelial ovarian, fallopian tube, or primary peritoneal cancer. However, despite the progress brought by bevacizumab, the prognostic of patients with AOC remains poor with a median PFS of 14-19 months and a median Overall Survival (OS) at 5 years of 30-35%.
In subgroup analysis of the ICON7 trial, the use of bevacizumab seems not to be able to improve OS for patients with complete surgery and no macroscopic residual disease. So, a part of these patients today did not receive bevacizumab after surgery (initial of interval surgery) if there is no residual disease (ESMO guidelines). In other hand, subset analysis of ICON7 and GOG 218 has suggested that patients with FIGO stage III and residual disease after initial surgery or FIGO stage IV are those who might most benefit from the addition of bevacizumab to first-line chemotherapy both in term of PFS and OS (20). These patients with extensive and bulky disease are often those whose initial surgery is delayed after 3 or 4 cycles of neo-adjuvant chemotherapy.
There is, indeed, some concern to administer bevacizumab during the chemotherapy surrounding the interval debulking surgery due to the long half-life (14- 21 days) of this monoclonal antibody and the interference of anti angiogenic agents with wound healing.
Finally, for patients where neo adjuvant chemotherapy is decided, as recommended by ESMO and national guidelines (Saint Paul de Vence), bevacizumab could only be introduced after interval surgery and not in combination with initial neo adjuvant chemotherapy. In such situation (neo adjuvant chemotherapy then interval surgery), the introduction of bevacizumab after interval debulking remains an option integrating benefit (residual disease or not after debulking surgery) and anticipated tolerability (digestive resection, systemic tolerance, no contraindication as instable arterial hypertension, major digestive resection at the time of the surgery, pulmonary embolism, etc. …).

B.4.3. Summary of the 1st line management for advanced disease

For patients whose extent of disease is not amenable to complete an optimal upfront debulking surgery, neo adjuvant treatment with carboplatin plus paclitaxel should be considered, followed by an interval debulking surgery. A minimum of 3 cycles of carboplatin/paclitaxel must be administered before to propose interval surgery. After interval surgery, completion of the chemotherapy with 3 or 4 more chemotherapy regimen is proposed.
For patients with macroscopic disease or when disease remains unresectable, combination with bevacizumab to adjuvant chemotherapy then a maintenance phase of bevacizumab alone can be proposed as a standard of care-French national guidelines Saint Paul de Vence (www.arcagy.org).
In France, it is estimated in 2015, 60% of advanced stage IIIC-IV ovarian carcinoma received bevacizumab in combination with standard chemotherapy after interval surgery (not published).

1.2 Background for Immunotherapy in ovarian carcinoma

1.2.1. OC is an immunogenic tumor: evidence

Immune surveillance plays an important role in tumor outcome of ovarian cancer patients (21). The earliest evidence on the role of immunotherapy in EOC came from observations that the presence of infiltrating T cells (called “tumor-infiltrating lymphocytes,” or TILs) is positively and strongly associated with improved survival (22). Indeed, clinical data in ovarian cancer patients have demonstrated that an antitumor immune response and immune evasion mechanisms are correlated, respectively, with a better and lower survival. Especially, tumor-associated macrophages (TAMs) that suppress anti-tumor immune responses have been reported to be associated with poor clinical outcome in ovarian cancer patients (23). This has been recently confirmed in a meta-analysis of 55 studies (n = 8,692 patients) having shown negative effects of TAM on OS in ovarian cancer patients [RR = 2.55 (95%CI, 1.60-4.06)] (24). Report from our research team has demonstrated that both local and systemic dysfunction of plasmacytoid Dendritic Cells (pDC) play a critical role in the progression of ovarian cancer via induction of immune tolerance (25). Thus, immunotherapies are emerging as potential strategies to enhance classical EOC treatments (22). Approximately half of OC patients display a spontaneous antitumor immune response by antibodies (Stone B et al 2003, Reuschenbach M, et al 2009) and oligoclonal T-cells (Schlienger et al , 2003) which recognize autologous tumor-associated antigens (TAAs). OC exhibits an extreme degree of heterogeneity of TAAs with an average of 60 private nonsynonymous mutations per tumor which are rarely shared among different tumors.
Though data remains scarce, high IHC PD-L1 expression (score 2 & 3) has been detected in 68% of ovarian cancer patients (n=70) and that expression of PD-L1 had a strong prognostic value (Hamanashi J, 2007). The authors found also that the density of intraepithelial CD8+ T cells was inversely correlated to expression of PD-L1 by tumors, suggesting that the expression of PD-L1 on tumor cells may inhibit invasion of tumor epithelium by CD8+ T cells.

In addition, PD-1 expression at the surface of intra-tumoral CD4+ FOXP3+ Tregs was found to show the highest levels in ovarian cancer (around 20% of the cells) compared to other tumor types, including melanoma, renal cell cancer or hepatoma (Kryczek et al., 2009). Thus targeting PD-1/PD-L1 pathway may inhibit Treg expression, one of the major component of ovarian cancer immunosuppression. Also Curiel et al showed that myeloid dendritic cells (MDCs) from ovarian cancer express PD-1 and that blockade of PD-1 enhanced MDC-mediated T-cell activation, including upregulation of IL-2 and interferon-gamma, and down regulation of IL-10, which resulted in enhanced T-cell immunity against autologous ovarian human tumors into NOD-SCID mice. (Curiel et al, 2003).

1.2.2. The PD-L1/PD1 immunosuppressive pathway in Ovarian Cancer

Immunotherapy strategies in EOC were initiated by using monoclonal Ab (mAb), vaccinations or adoptive T-cell transfers (22). The firsts in class are monoclonal antibodies (mAbs) targeting molecules expressed at the surface of immune cells called CTLA4, PD-1 and its ligand PD-L1. In particular the immune-checkpoint receptor, programed cell death 1 (PD1) and its ligands PDL1 and PLD2, are emerging as promising targets. These immunostimulatory mAbs have well demonstrated their monotherapy efficacy in patients bearing Metastatic Melanoma (26, 27, 28, 29) and their synergistic activity when used in combination (30). More recently, they also have demonstrated significant efficacy in aggressive cancers of other histology such as metastatic Lung Cancer (31), metastatic Renal Cell Cancer or metastatic Bladder Cancer (ASCO 2014). Together with the aforementioned data on immune infiltration, these data provide the rationale for a therapeutic PD-1/PD-L1 pathway blockade in ovarian cancer.
Interestingly, in the BMS anti-PD-L1 phase I trial, early signs of activity have been seen in patients with ovarian carcinoma: 3 of 18 (17%) patients with ovarian carcinoma had an objective response and 2 had a CR. The interaction of PD-1 with PD-L1 highly suppresses anti-tumor cytotoxic T-cells. Monoclonal antibodies against PD-1 and PD-L1 have demonstrated encouraging signs of antitumor activity for patients with pretreated advanced ovarian cancer, according to findings from two studies presented at the 2015 ASCO Annual Meeting. In ovarian carcinoma patients, the anti-PD1 compound nivolumab has been reported to achieve 3 objectives responses out of 13 (23%) heavily pre-treated patients (Hamanishi J, ASCO 2014). Response was prolonged over 1 year in 2 out of the 3 responders (Hamanishi J, ASCO 2015). In the first phase IB trial, the PD-L1 inhibitor avelumab demonstrated clinical activity and a low toxicity profile in treatment-refractory unselected patients with ovarian cancer. The anti-PD-L1 avelumab has reported a 10.7% objective response and a 44% stabilization rate in 75 patients with ovarian cancer in relapse (Disis M et al, 2015). In this study, confirmed or unconfirmed responses (n=11) tend to be more frequently observed in patients with low burden of tumor, limited number of prior lines of chemotherapy and in the setting of platinum-sensitivity. Toxicity was minimal. Considering all grades, fatigue was observed in 16% of the patients, chills in 12%, nausea in 10.7%, diarrhea in 10.7%, rash in 8% and hypothyroidism in 5.3%.
Additionally, in a separate phase IB study, the PD-1 inhibitor pembrolizumab demonstrated activity in heavily pre-treated PD-1-positive patients. The anti-PD1 pembrozilumab achieved 3 confirmed responses (11.5% [(95% CI, 2.4-30.2]) in 26 patients treated in a phase IB study and 3 additional patients had a tumor reduction of at least 30%. Most common Aes were fatigue (42.3%), anemia (30.8%), and decreased appetite (30.8%). Drug-related Aes occurred in 69.2% of pts (grade ≥ 3, 1/26 pts) (Varga A et al, 2015).Side effects in both studies were mild and generally manageable. – See more at: http://www.onclive.com/conference-coverage/asco-2015/PD-1PD-L1-Inhibitors-Safe-Effective-in-Advanced-Ovarian-Cancer#sthash.MS8wP3Vh.dpuf.

1.2.3 Rationale for combining pembrolizumab and standard chemotherapy

Kryczek et al compared the PD-1 expression level at the surface of intra-tumoral CD4+ FOXP3+ Tregs among many cancer types. Interestingly, the higher level of PD-1 expression (around 20%) was found on Tregs of ovarian cancers whereas it was much lower (<10%) in other cancer types (Colon cancer, Hepatic cancer, Melanoma, Pancreatic carcinoma, Renal cell carcinoma) (58). PD-L1 expression has also been detected in ovarian cancer tissue analysis by Immunohistochemistry staining and its level of expression has been correlated to a bad outcome of patients (59). Together with the aforementioned data on immune infiltration, these results provide rationale for a therapeutic PD-1/PD-L1 pathway blockade in ovarian cancer. In the published trials on such compounds, addition of pembrolizumab to chemotherapy or using alone has been shown to improve the response rates with a median time to response at 8 weeks (60,61).

1.2.4 Rationale for combining pembrolizumab and bevacizumab

Given that both VEGF and PD-L1 appear important in OC pathogenesis, this study is designed to test the hypothesis that inhibition of VEGF signaling will enhance the efficacy of immunotherapy in the treatment of patients with late OC relapse. There are several data suggesting that pembrolizumab and bevacizumab may be synergistic.
Enhanced tumor angiogenesis is commonly associated with absence of tumor-infiltrating T cells in patients (Bouma-ter Steege JC et al, 2004). There is evidence in OC that tumor expression of VEGF is negatively correlated to the density of CD3+TILs (Zhang L et al 2003) and this phenotype is associated with early recurrence, consistent with prior studies showing a correlation of VEGF to early recurrence and short survival. Furthermore, in ascites, high levels of VEGF correlate to low numbers of NK T-like CD3+CD56+ cells (Bamias et al, 2008).

In addition to promoting tumor angiogenesis, there is increasing evidence that VEGF plays a role in cancer immune evasion through several different mechanisms (Ohm et al 2001). For example, experiments with activated endothelial cells suggest that in the tumor microenvironment, VEGF may reduce lymphocyte adhesion to vessel walls, thus contributing to decreased immune-cell recruitment to the tumor site (Bouzin Cet al. 2007, Griffoen AW et al, 1996). Indeed, emerging evidence suggests that the endothelium acts as a selective barrier, allowing certain T cell subsets, notably T regulatory (Treg) cells, to traffic more effectively contributing to tumor immune tolerance (Motz GT et al, 2014). In addition, some experiments have shown that tumour hypoxia promotes the recruitment of regulatory T (T reg)) cells through induction of expression of the chemokine CC-chemokine ligand 28 (CCL28), which, in turn, promotes tumor tolerance and angiogenesis. Conversely, angiogenesis blockade requires CD8+T cell (Manning EA et al, 2007, Motz GT et al, 2014) supporting the notion that VEGF-A do not simply promote tumor growth through angiogenesis. Thus, peripheral immune tolerance and angiogenesis programs seems closely connected and to cooperate to sustain tumour growth (Facciabene A et al, 2011; Kandalaft LE, 2011).
In addition, there is evidence that anti-VEGF therapy and immunotherapy acts synergistically. Motz et al have suggested that the combination of anti-VEGF-A antibody and immunotherapy with adoptive T cell transfer led to a superior infiltration of tumor-reactive T cells than single approach (Motz GT et al, 2014). Indeed, in a murine melanoma model, VEGF blockade synergized with adoptive immunotherapy, as evidenced by improved anti-tumor activity, prolonged survival, and increased trafficking of T cells into tumors (Shrimali et al. 2010). More evidence has come from a clinical study of subjects with melanoma combining a checkpoint inhibitor (anti�CTLA-4; ipilimumab) and bevacizumab (Hodi FS et al, 2014; Garber K, 2014). In 46 patients, the combined therapy yielded a 19.6% objective response rate, stable disease in 13%. All responses were durable > 6 months and median survival was 25.1 months, much prolonged compared to ipilimumab expectation in metastatic melanoma. Thus, an emerging paradigm supported by the previous data is that angiogenesis and immune suppression are two facets of a linked biological program. Tumors seem to co-opt these existing mechanisms that are normally required to limit excessive inflammation and promote tissue recovery during infection or wound healing. The execution of this program sustains tumor growth and promotes immunologic tolerance.

B.4. Informations on Pembrolizumab

Refer to the Investigator's Brochure (IB)/approved labeling for detailed background information on MK-3475.

1.3.1. Pharmaceutical and Therapeutic Background

The importance of intact immune surveillance in controlling outgrowth of neoplastic transformation has been known for decades (32). Accumulating evidence shows a correlation between tumor-infiltrating lymphocytes (TILs) in cancer tissue and favorable prognosis in various malignancies. In particular, the presence of CD8+ T-cells and the ratio of CD8+ effector T-cells / FoxP3+ regulatory T-cells seems to correlate with improved prognosis and long-term survival in many solid tumors (33, 34).
The PD-1 receptor-ligand interaction is a major pathway hijacked by tumors to suppress immune control. The normal function of PD-1, expressed on the cell surface of activated T-cells under healthy conditions, is to down-modulate unwanted or excessive immune responses, including autoimmune reactions. PD-1 (encoded by the gene Pdcd1) is an Ig superfamily member related to CD28 and CTLA-4 which has been shown to negatively regulate antigen receptor signaling upon engagement of its ligands (PD-L1 and/or PD-L2) (3, 36). The structure of murine PD-1 has been resolved (37). PD-1 and family members are type I transmembrane glycoproteins containing an Ig Variable-type (V-type) domain responsible for ligand binding and a cytoplasmic tail which is responsible for the binding of signaling molecules. The cytoplasmic tail of PD-1 contains 2 tyrosine-based signaling motifs, an immunoreceptor

tyrosine-based inhibition motif (ITIM) and an immunoreceptor tyrosine-based switch motif (ITSM). Following T-cell stimulation, PD-1 recruits the tyrosine phosphatases SHP-1 and SHP-2 to the ITSM motif within its cytoplasmic tail, leading to the dephosphorylation of effector molecules such as CD3ζ, PKCθ and ZAP70 which are involved in the CD3 T-cell signaling cascade (36, 38, 39, 40). The mechanism by which PD-1 down modulates T-cell responses is similar to, but distinct from that of CTLA-4 as both molecules regulate an overlapping set of signaling proteins (41, 42). PD-1 was shown to be expressed on activated lymphocytes including peripheral CD4+ and CD8+ T-cells, B-cells, T regs and Natural Killer cells (43, 44). Expression has also been shown during thymic development on CD4-CD8-(double negative) T-cells as well as subsets of macrophages and dendritic cells (45). The ligands for PD-1 (PD-L1 and PD-L2) are constitutively expressed or can be induced in a variety of cell types, including non-hematopoietic tissues as well as in various tumors (32, 42, 46, 47, 48). Both ligands are type I transmembrane receptors containing both IgV- and IgC-like domains in the extracellular region and contain short cytoplasmic regions with no known signaling motifs. Binding of either PD-1 ligand to PD-1 inhibits T-cell activation triggered through the T-cell receptor. PD-L1 is expressed at low levels on various non-hematopoietic tissues, most notably on vascular endothelium, whereas PD-L2 protein is only detectably expressed on antigen-presenting cells found in lymphoid tissue or chronic inflammatory environments. PD-L2 is thought to control immune T-cell activation in lymphoid organs, whereas PD-L1 serves to dampen unwarranted T-cell function in peripheral tissues (42). Although healthy organs express little (if any) PD-L1, a variety of cancers were demonstrated to express abundant levels of this T-cell inhibitor. PD-1 has been suggested to regulate tumor-specific T-cell expansion in subjects with melanoma (MEL) (49). This suggests that the PD-1/PD-L1 pathway plays a critical role in tumor immune evasion and should be considered as an attractive target for therapeutic intervention.

1.3.2. Preclinical and Clinical Trial Data

Pembrolizumab is a potent and highly selective humanized monoclonal antibody (mAb) of the IgG4/kappa isotype designed to directly block the interaction between PD-1 and its ligands, PD-L1 and PD-L2. Keytruda™ (pembrolizumab) has recently been approved in the United Stated for the treatment of patients with unresectable or metastatic melanoma and disease progression following ipilumumab and, if BRAF V600 mutation positive, a BRAF inhibitor.

The programmed cell death 1 (PD-1) pathway represents a major immune control switch, which may be engaged by tumor cells to overcome active T-cell immune surveillance. This blockade enhances functional activity of the target lymphocytes to facilitate tumor regression and ultimately immune rejection. Merck, Sharp & Dohme Corporation, a subsidiary of Merck & Co., Inc. (herein referred to as Merck), is studying pembrolizumab for various oncology indications. As of the data cutoff dates for this Investigational Brochure (IB) (18-Apr-2014 for P001 melanoma subjects, 29-Aug-2014 for P001 non-small cell lung cancer (NSCLC) subjects, 12-May-2014 for P002, and 30-Nov-2014 for all other protocols), pembrolizumab monotherapy and combination therapy have been administered to 6294 subjects with hematologic malignancies and solid tumors, in a total of 18 ongoing, Phase I, II, and III clinical trials sponsored by Merck (Table 19, BI).

As of 30-Nov-2014, pembrolizumab has also been administered to the following:

• Eighty-nine subjects enrolled in 1 of 3 clinical studies that are part of Merck's Oncology Collaborative Studies Program, which supports investigator-initiated and –led studies and provides investigational/marketed Merck drugs for such studies.

• Forty-two subjects enrolled in 5 clinical studies with a sponsor other than Merck.

On 04-Sep-2014, the United States (U.S.) Food and Drug Administration (FDA) granted accelerated approval to KEYTRUDA® for treatment of subjects with unresectable or metastatic melanoma and disease progression following treatment with ipilimumab (IPI) and, if BRAF V600 mutation positive, a BRAF inhibitor. The recommended dose of KEYTRUDA is 2 mg/kg administered as an intravenous (IV) infusion over 30 minutes every 3 weeks (Q3W) until disease progression or unacceptable toxicity. This indication was approved based on tumor response rate and durability of response (data cutoff date 18-Oct-2013) observed in a total of 411 IPI-refractory plus IPI-naïve melanoma subjects enrolled in P001 and treated with 1 of 3 dose regimens of pembrolizumab (2 mg/kg Q3W, 10 mg/kg Q3W, or 10 mg/kg every 2 weeks [Q2W]). An improvement in survival or disease-related symptoms had not yet been established at the time of the FDA filing. A cutoff date of 31-Dec-2014 was used for post marketing information presented in this IB.

1.3.4. Nonclinical Pharmacology

Pembrolizumab binds to human and Cynomolgus monkey PD-1 with comparable affinity and blocks the binding of human and Cynomolgus monkey PD-1 to PD-L1 and PD-L2 with comparable potency. Pembrolizumab does not cross-react with dog, rat, or mouse PD-1. Pembrolizumab does not bind immunoglobulin superfamily members cluster of differentiation 28 (CD28), cytotoxic T-lymphocyte-associated protein 4 (CTLA-4), or inducible T-cell costimulator (ICOS). Pembrolizumab strongly enhances T-lymphocyte immune responses in cultured blood cells from healthy human donors, cancer subjects, and nonhuman primates. In T-cell activation assays using human donor blood cells, the half-maximal effective concentration (EC50) has been approximately 0.1 to 0.3 nM. In addition to interleukin-2 (IL-2), tumor necrosis factor alpha (TNFα), interferon gamma (IFN[illegible]), and levels of other cytokines were found to be modulated by pembrolizumab. The antibody potentiates existing immune responses only in the presence of antigen and does not nonspecifically activate T-cells. In the in vitro peripheral blood mononuclear cell (PBMC) and whole blood cytokine release assays, the cytokine levels induced by pembrolizumab were low and comparable to those induced by trastuzumab. Pembrolizumab does not induce antibody-dependent cell-mediated cytotoxicity (ADCC) or complement-dependent cytotoxicity (CDC). Using anti–murine PD-1 surrogate antibodies, PD-1 blockade has been shown to significantly inhibit tumor growth in a variety of syngeneic murine tumor models. In these experiments in mice, anti–PD-1 therapy is synergistic with chemotherapeutic agents such as gemcitabine and 5-fluorouracil (5-FU), and combination therapy results in increased complete tumor regression rates in vivo. Studies also revealed that immunosuppressive doses of dexamethasone included in combination with agents used in standard-of-care treatment for NSCLC do not reduce the anti-tumor efficacy of an anti–murine PD-1 surrogate antibody.

1.3.5. Nonclinical Pharmacokinetics

The pharmacokinetics (PK) of pembrolizumab were evaluated in a non–Good Laboratory Practices (GLP) single dose PK study and two GLP repeat-dose toxicokinetic (TK) studies (1 month and 6 month) in Cynomolgus monkeys. Pembrolizumab stability as a modified IgG4 molecule was evaluated in vivo in mice. After single-dose IV administration at 0.3, 3, or 30 mg/kg in Cynomolgus monkeys, decline of serum concentration followed multiphasic kinetics. Anti–drug antibodies (ADAs) were detected in most of the treated animals. Clearance (CL) and terminal half-life (t1/2) appeared to be dose-dependent in the dose range tested with t1/2 varying from 4 to 10 days. In the 1-month repeat-dose (once weekly) GLP toxicity study at 6, 40, or 200 mg/kg in Cynomolgus monkeys, ADAs were detected in most of the low-dose (6 mg/kg) treated animals. The systemic exposure over the 7-day dosing interval (AUC0-7 days) was sex-independent and increased with increasing dose. The mean t1/2 values in individual ADA-negative animals ranged from 15.7 to 22.3 days across doses. In the 6-month repeat-dose (every other week) GLP toxicity study at 6, 40, or 200 mg/kg in Cynomolgus monkeys, ADAs were detected in most of the low-dose (6 mg/kg) treated animals. The systemic exposure to pembrolizumab was independent of sex and was approximately dose-proportional across all doses. The mean t1/2 values in individual ADA-negative animals ranged from 21 to 22 days across doses. IgG4 wild type molecule can undergo in vivo molecular rearrangement called Fab-arm (or half molecule) exchange by swapping their half molecule with other IgG4 half molecules, thereby generating bispecific or hybrid antibodies. Pembrolizumab is a hinge mutated IgG4 (S228P), which prevents in vivo half-molecule swap (formation of hybrid). An in vivo mice experiment has demonstrated that pembrolizumab did not form hybrid antibody with another wild type IgG4 molecule.

1.3.6 Safety Pharmacology/Toxicology

The potential for systemic toxicity of pembrolizumab was assessed in a 1-month repeat-dose toxicity study with a 4-month recovery in Cynomolgus monkeys and in a 6-month repeat-dose toxicity study with a 4-month recovery period in Cynomolgus monkeys. In the 1-month toxicity study, Cynomolgus monkeys were administered an IV dose of 6, 40, or 200 mg/kg once weekly for a total of 5 doses. Four monkeys/sex/group were euthanized during Week 5. The remaining 2 monkeys/sex/group were euthanized during Week 23, after a 4-month post dose period. In this study, pembrolizumab was well-tolerated in monkeys with the systemic exposure (AUC) up to approximately 170,000 µg·day/mL over the course of the study. There was no test article–related mortality, and test article–related changes were limited to an increased incidence of inguinal swelling, and increased splenic weights in males receiving 200 mg/kg. Both of these findings were not considered adverse and there was no histopathologic correlation. Splenic weights were normal at the post dose necropsy. Anti–pembrolizumab antibodies were detected in 7 out of 8 animals in the 6 mg/kg dose group and 1 animal out of 8 in the 40 mg/kg dose group and were associated with an apparent increase in clearance of pembrolizumab. The presence of ADA in monkeys in the low-dose group and in 1 monkey in the mid-dose group did not impact the pharmacodynamic response, because sufficient target

engagement was demonstrated for the duration of the study (with the exception of 1 low-dose monkey). Additionally, anti–pembrolizumab antibodies were not detected in any monkeys in the high-dose group, suggesting that potential toxicity has been evaluated at the highest exposure levels in the study. Based on the lack of adverse test article–related findings in this study, the no observed adverse effect level (NOAEL) was ≥200 mg/kg.
In the 6-month toxicity study, the potential for systemic toxicity was assessed in Cynomolgus monkeys administered an IV dose of 6, 40, or 200 mg/kg once every other week for approximately 6 months (a total of 12 doses) followed by a 4-month treatment-free period. Three animals/sex/group were designated for interim necropsy at the end of the 6-month animals. The systemic exposure over the 7-day dosing interval (AUC0-7 days) was sex-independent and increased with increasing dose. The mean t1/2 values in individual ADA-negative animals ranged from 15.7 to 22.3 days across doses. In the 6-month repeat-dose (every other week) GLP toxicity study at 6, 40, or 200 mg/kg in Cynomolgus monkeys, ADAs were detected in most of the low-dose (6 mg/kg) treated animals. The systemic exposure to pembrolizumab was independent of sex and was approximately dose-proportional across all doses. The mean t1/2 values in individual ADA-negative animals ranged from 21 to 22 days across doses. IgG4 wild type molecule can undergo in vivo molecular rearrangement called Fab-arm (or half molecule) exchange by swapping their half molecule with other IgG4 half molecules, thereby generating bispecific or hybrid antibodies. Pembrolizumab is a hinge mutated IgG4 (S228P), which prevents in vivo half-molecule swap (formation of hybrid). An in vivo mice experiment has demonstrated that pembrolizumab did not form hybrid antibody with another wild type IgG4 molecule.

1.3.7 Clinical Development

Safety data are presented in this IB for a total of 2461 subjects with melanoma or NSCLC in 8 ongoing, Merck-sponsored clinical trials: P001, P002, P011, P012, P013, P021, P023, and P028. Subjects received 1 of 6 different pembrolizumab dose regimens, either as monotherapy (P001, P002, P011, P012, P013, P028) or as combination therapy (P021, P023). Note that P011 is a combination therapy trial in which pembrolizumab monotherapy was compared with pembrolizumab in combination with cisplatin/pemetrexed and carboplatin/paclitaxel. However, only pembrolizumab monotherapy data are presented in this IB. Adverse events were generally manageable and infrequently required discontinuation of pembrolizumab treatment. Efficacy data are presented in this IB for 655 melanoma subjects treated with pembrolizumab in P001 and 540 melanoma subjects treated with either pembrolizumab or chemotherapy in P002. The overall response rates for pembrolizumab treatment in P001 and P002 compared favorably to historical response rates for available treatments for melanoma, particularly in subjects who have progressed after multiple prior therapies. PK data are presented in this IB from a total of 1818 PK-evaluable subjects with advanced melanoma (P001 [Parts B and D], P002), NSCLC (P001 Parts C and F), advanced solid tumors (including triple negative breast cancer [TNBC], head and neck cancer, urothelial tract cancer, gastric cancer [P012]), and hematological cancers (P013 and P023). The observed PK profile of pembrolizumab was typical when compared with other immunoglobulin G (IgG) mAbs with a half-life (t1/2) of approximately 3 weeks.

Pembrolizumab & ovarian cancer

In a phase IB study reported at the ASCO 2015 meeting for patients with ovarian carcinoma, membranous PD-L1-positivity (≥1% expression) was assessed using the 22C3 antibody clone. Of the 96 patients screened, 49 (51%) were deemed PD-L1-positive. These patients went on to receive pembrolizumab at 10 mg/kg every 2 weeks, with 26 evaluable for response. The median age of patients enrolled was 57.5 years, the majority of patients were white (61.5%), the primary histology was adenocarcinoma (46.2%), and 34.6% of patients had high-grade serous carcinoma. Adjuvant or neo adjuvant therapy was administered to 53.8% of patients, and 80.8% of patients had received ≥4 prior therapies. The 11.5% ORR was comprised of 1 complete response (3.8%) and 2 partial responses (7.7%). Tumor shrinkage of less than 30% was seen in an additional 11.5% of patients. Six patients (23.1%) had stable disease, for a DCR of 34.6%. The median time to response was 8 weeks and the median response duration was not yet reached. All-grade adverse events were apparent in 69.2% of patients, including arthralgia (23.1%), diarrhea (11.5%), nausea (11.5%), hypothyroidism (11.5%), and fatigue (7.7%). Hyperthyroidism, pruritus, rash, and thrombocytopenia were seen in 7.7% of patients each. The only high-grade side effect was transaminases increase (3.8%). Pembrolizumab has a manageable safety and toxicity profile," reported the authors. There was not mortality related to treatment and there were no discontinuations due to treatment-related adverse events. Pembrolizumab demonstrated antitumor activity in heavily pretreated metastatic ovarian cancer patients. Analyses of the relationship between response and PD-L1 expression, as well as other potential predictive biomarkers are still ongoing.

Merck-sponsored clinical trials: P001, P002, P011, P012, P013, P021, P023, and P028. Subjects received 1 of 6 different pembrolizumab dose regimens, either as monotherapy (P001, P002, P011, P012, P013, P028) or as combination therapy (P021, P023). Note that P011 is a combination therapy trial in which pembrolizumab monotherapy was compared with pembrolizumab in combination with cisplatin/pemetrexed and carboplatin/paclitaxel.

COMBINATION OF BEVACIZUMAB AND PEMBROLIZUMAB (MK-3475)

Concerning rational for combining bevacicumab and pembrolizumab, there is more and more data that the vascular endothelial growth factor (VEGF) plays a crucial role also as chemoattractant for inflammatory cells, including macrophages, neutrophils, dendritic cells (DCs), myeloid-derived suppressor cells (MDSCs) and T-cells (50-53). Therefore, blocking VEGF with Bevacizumab translates into immunomodulatory effects (increase of T cells trafficking into tumors and reduction of suppressive cytokines and infiltrating Tregs and MDSCs) being synergic with other immunotherapies. These data represent the background for association of chemotherapy and immunotherapy with Bevacizumab. Accordingly, a study demonstrating the safety and efficacy of MPDL3280A in combination with Bevacizumab and/or chemotherapy in patients with locally advanced or metastatic solid tumors has been presented at the ESMO 2014 meeting (54-56), and another study demonstrating the safety and efficacy of MPDL3280A in combination with Bevacizumab in Metastatic Renal Cell Carcinoma has been presented at the ASCO GU 2016 meeting (57).

1.4 Rationale

1.4.1 RATIONALE FOR THE TRIAL AND SELECTED SUBJECT POPULATION

Subset analysis of ICON7 and GOG 218 has suggested that patients with FIGO stage III and residual disease after initial surgery or FIGO stage IV are those who might most benefit from the addition of new agents to first-line chemotherapy both in term of PFS and OS. These patients with extensive and bulky disease are often those whose initial surgery is delayed (4) after 3 or 4 cycles of neo-adjuvant chemotherapy. There is, indeed, some concern to administer bevacizumab during the chemotherapy surrounding the interval debulking surgery due to the long half-life (14- 21 days) of this monoclonal antibody and the interference of anti angiogenic agents with wound healing.

Kryczek et al compared the PD-1 expression level at the surface of intra-tumoral CD4+ FOXP3+ Tregs among many cancer types. Interestingly, the higher level of PD-1 expression (around 20%) was found on Tregs of ovarian cancers whereas it was much lower (<10%) in other cancer types (Colon cancer, Hepatic cancer, Melanoma, Pancreatic carcinoma, Renal cell carcinoma) (58). PD-L1 expression has also been detected in ovarian cancer tissue analysis by Immunohistochemistry staining and its level of expression has been correlated to a bad outcome of patients (59). Together with the aforementioned data on immune infiltration, these results provide rationale for a therapeutic PD-1/PD-L1 pathway blockade in ovarian cancer. In the published trials on such compounds, addition of pembrolizumab to chemotherapy or using alone has been shown to improve the response rates with a median time to response at 8 weeks (60,61). We assume that its administration in the neo adjuvant setting combination with standard of care (4 cycles of standard chemotherapy) would improve the response rate and consequently will help to achieve optimal debulking rate at IDS.Pembrolizumab (MK-3475) might offer a better alternative to bevacizumab in the neo-adjuvant setting. Preliminary experience in cancer and mecanism of action did not show a trend for increased incidence of surgical toxicity. For more details please refer to the investigator drug brochure for Pembrolizumab (MK-3475).

1.4.2 RATIONALE FOR DOSE SELECTION/REGIMEN/MODIFICATION FOR PEMBROLIZUMAB

An open-label Phase I trial (Protocol 001) is being conducted to evaluate the safety and clinical activity of single agent MK-3475. The dose escalation portion of this trial evaluated three dose levels, 1 mg/kg, 3 mg/kg, and 10 mg/kg, administered every 2 weeks (Q2W) in subjects with advanced solid tumors. All three dose levels were well tolerated and no dose-limiting toxicities were observed. This first in human study of MK-3475 showed evidence of target engagement and objective evidence of tumor size reduction at all dose levels (1 mg/kg, 3 mg/kg and 10

mg/kg Q2W). No MTD has been identified to date. 10.0 mg/kg Q2W, the highest dose tested in PN001, will be the dose and schedule utilized in Cohorts A, B, C and D of this protocol to test for initial tumor activity. Recent data from other clinical studies within the MK-3475 program has shown that a lower dose of MK-3475 and a less frequent schedule may be sufficient for target engagement and clinical activity.

Pharmacokinetic (PK) data analysis of MK-3475 administered Q2W and Q3W showed slow systemic clearance, limited volume of distribution, and a long half-life (refer to IB). Pharmacodynamic data (IL-2 release assay) suggested that peripheral target engagement is durable (>21 days). This early PK and pharmacodynamic data provides scientific rationale for testing a Q2W and Q3W dosing schedule.
A population for pharmacokinetic analysis has been performed using serum concentration time data from 476 patients. Within the resulting population PK model, clearance and volume parameters of MK-3475 were found to be dependent on body weight. The relationship between clearance and body weight, with an allometric exponent of 0.59, is within the range observed for other antibodies and would support both body weight normalized dosing or a fixed dose across all body weights. MK-3475 has been found to have a wide therapeutic range based on the melanoma indication. The differences in exposure for a 200 mg fixed dose regimen relative to a 2 mg/kg Q3W body weight based regimen are anticipated to remain well within the established exposure margins of 0.5 – 5.0 for MK-3475 in the melanoma indication. The exposure margins are based on the notion of similar efficacy and safety in melanoma at 10 mg/kg Q3W vs. the proposed dose regimen of 2 mg/kg Q3W (i.e. 5-fold higher dose and exposure). The population PK evaluation revealed that there was no significant impact of tumor burden on exposure. In addition, exposure was similar between the NSCLC and melanoma indications. Therefore, there are no anticipated changes in exposure between different indication settings.

The rationale for further exploration of 2 mg/kg and comparable doses of pembrolizumab in solid tumors is based on: 1) similar efficacy and safety of pembrolizumab when dosed at either 2 mg/kg or 10 mg/kg Q3W in melanoma patients, 2) the flat exposure-response relationships of pembrolizumab for both efficacy and safety in the dose ranges of 2 mg/kg Q3W to 10 mg/kg Q3W, 3) the lack of effect of tumor burden or indication on distribution behavior of pembrolizumab (as assessed by the population PK model) and 4) the assumption that the dynamics of pembrolizumab target engagement will not vary meaningfully with tumor type.
The choice of the 200 mg Q3W as an appropriate dose for the switch to fixed dosing is based on simulations performed using the population PK model of pembrolizumab showing that the fixed dose of 200 mg every 3 weeks will provide exposures that 1) are optimally consistent with those obtained with the 2 mg/kg dose every 3 weeks, 2) will maintain individual patient exposures in the exposure range established in melanoma as associated with maximal efficacy response and 3) will maintain individual patients exposure in the exposure range established in melanoma that are well tolerated and safe.
A fixed dose regimen will simplify the dosing regimen to be more convenient for physicians and to reduce potential for dosing errors. A fixed dosing scheme will also reduce complexity in the logistical chain at treatment facilities and reduce wastage.

1.4.3 Rationale for combination

Pembrolizumab in this current study is expected to have a positive benefit-risk profile for the treatment of patients with late relapse of OC.
Platinum-containing therapy is considered the treatment of choice for patients with platinum-sensitive relapse. However the duration of response and the prolongation of the progression free interval are usually brief and these chemotherapy regimens cannot be continued until progression as they are associated with neurological, renal and hematological toxicity and cannot generally be tolerated for more than about 6 to 9 cycles.
Recently, the European Medicines Agency (EMA) approved bevacizumab, in combination with carboplatin and gemcitabine, for the first recurrence of platinum sensitive epithelial ovarian, fallopian tube, or primary peritoneal cancer; the approval was based on improved PFS in trials of bevacizumab in combination with chemotherapy followed by bevacizumab maintenance monotherapy. Despite the progress brought by bevacizumab, median PFS of late relapsing OC patients does not exceed 13.8 months. There is thus a need for a more effective treatment to extend the progression free interval in this patient population. Immune checkpoint inhibitors such as anti-PD1 or anti-PD-L1 have demonstrated to yield prolonged disease control in heavily pre-treated OC patients.
The use of pembrolizumab concurrent to their platinum-containing plus bevacizumab regimen and as a maintenance therapy after completion of chemotherapy as per normal clinical practice may provide further benefit for

patients in terms of prolongation of the progression free interval, increasing the interval between lines of chemotherapy, delaying further hospitalization and the cumulative toxicities associated with chemotherapy. Additionally, pembrolizumab preliminary studies suggest an acceptable tolerability profile for long term clinical use in a clearly identifiable patient population most likely to derive benefit.

2. INVESTIGATIONAL PLAN

2.1. Study Design

The proposed trial is a non-comparative, open label, randomized, multicentric, phase 2 study.

Randomization will be performed by minimization on a 1:2 ratio.

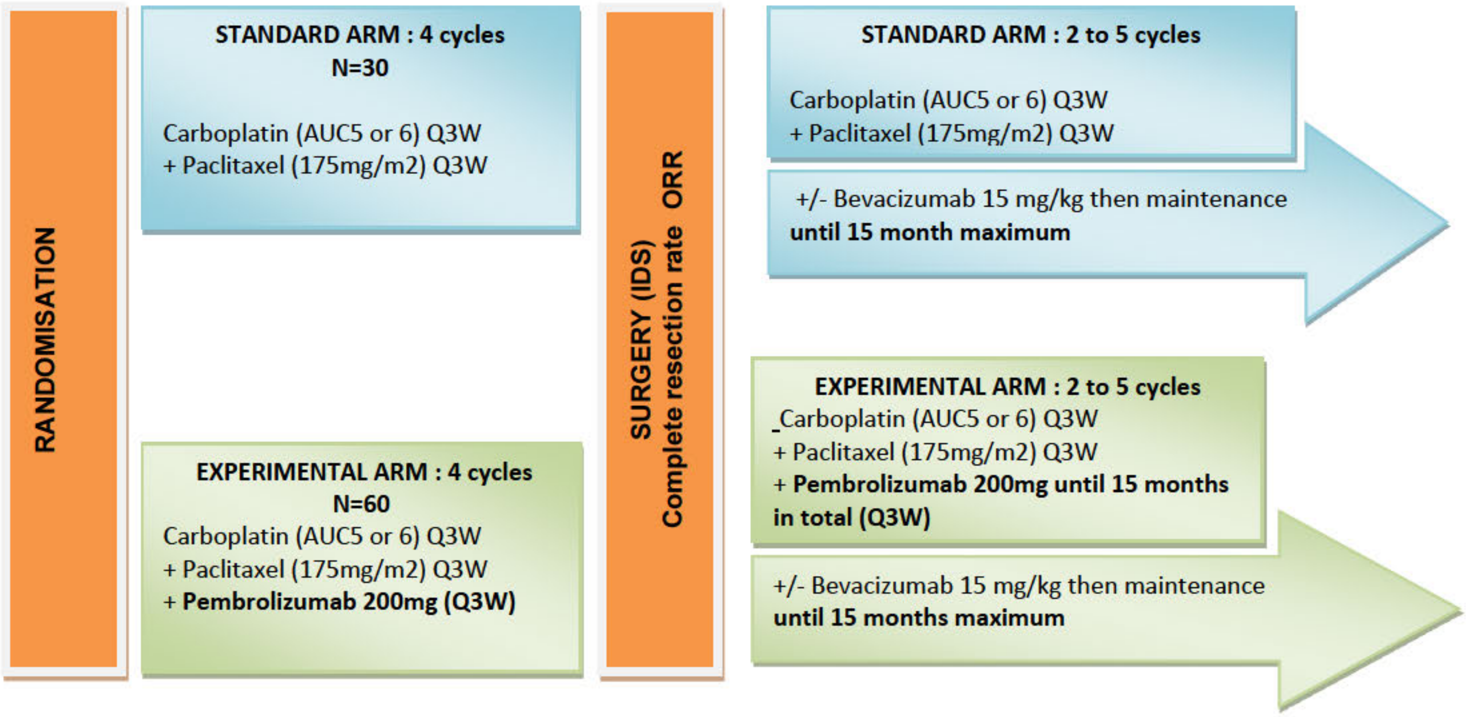

STRATIFICATION FACTORS

- Centers
- FIGO stage (IIIC versus IV)
- Matastasis volume (< 5cm versus ≥ 5 cm)
- Planned used of Bevacizumab after IDS (either arm A or B) (yes or no)

*BEVACIZUMAB MAY BE INTRODUCED IN BOTH ARMS AFTER INTERVAL SURGERY AS STANDARD OF CARE

Neo-Adjuvant therapy

- Arm A (n=30): 4 neo-adjuvant cycles of standard 3 weekly **carboplatin (AUC5 or 6) and paclitaxel (175mg/m²)**
- Arm B (n=60): 4 neo-adjuvant cycles of standard 3 weekly **Pembrolizumab 200 mg then carboplatin (AUC5 or 6) and paclitaxel (175mg/m²)**

Interval debulking surgery (IDS)

A mandatory laparoscopy with resectability evaluation should be performed 4 weeks +/- 2 week after the last (4th cycle) chemotherapy +/- experimental drug administration with the aim to perform IDS. Sugarbaker Index will be assessed during laparoscopy.
If IDS is postponed (unresectable disease), multiple biopsies through laparoscopy will be required.
If laparoscopy could not be performed after 4 cycles, due to other reason than progression, a contact with the Trial Manager will be required.

Adjuvant therapy

Adjuvant therapy will start after complete surgical wound healing and under no circumstances the treatment will start 4 weeks after surgery (+/- 2 weeks). It optimally should not start beyond 6 weeks after surgery.

- Arm A: **carboplatin (AUC5 or 6) and paclitaxel (175mg/m²)**, q3 weeks +/- **bevacizumab** (15 mg/kg Q3W)
- Arm B: **Pembrolizumab 200 mg** then **carboplatin (AUC5 or 6) and paclitaxel (175mg/m²)**, +/- **bevacizumab** (15 mg/kg Q3W)

The total number of cycles of chemotherapy will be 6 cycles from the start of the neo-adjuvant chemotherapy. Three additional cycles are allowed if required (maximum 9 cycles in total).

The planning of administration or not of bevacizumab will be defined before the randomization and could not be modified (stratification factor).

Maintenance therapy for Arm A & B

After chemotherapy:

- In the Arm A & B, for patients receiving bevacizumab (as standard therapy) (15 mg/kg Q3W), this will be continue until a maximum of 15 months in total from the beginning of the adjuvant therapy.
- In the arm B, patient will receive Pembrolizumab 200 mg until a maximum of 15 months in total from the beginning of the adjuvant therapy.

2.2. Study objectives and associated endpoints

2.2.1. Primary objective

The primary objective is **to evaluate the efficacy** of neo adjuvant pembrolizumab and chemotherapy or chemo-therapy alone measured by the **complete resection rate** (CCO) after interval debulking surgery and evaluated by a central medical review. Complete resection will be defined as the removal of all macroscopic residual tumor (CC score = 0) (Appendix 3)

2.2.2. Secondary objective

To evaluate the efficacy of the addition of pembrolizumab to chemotherapy treatment assessed by :

- **CCI score** evaluated by centers and by central review
- PCI score evaluated by centers and by central review
- The Objective Response Rate assessed by CT-Scan (RECIST 1.1) at the time of interval debulking surgery (after 4 neo adjuvant cycles of treatment)
- The Best Overall Response to the global strategy of interval debulking surgery + chemotherapy +/- Pembrolizumab assessed by CT-Scan (using RECIST 1.1) at the end of treatment visit & before interval surgery.
- The rate of Pathologic Complete Response (pCR) after surgery
- The Progression-Free Survival (PFS)
- Biological Progression-Free Interval (PFIBIO), measured according to the GCIG criteria.
- Overall survival (OS)

To assess the safety profile of neo adjuvant and adjuvant pembrolizumab when combined with standard of care, according to NCI CTC-AE v4.03.

- **To assess** Post-operative mortality
  To assess Post-operative morbidity according to modified Clavien Dindo scoring Radiological responses will be assessed by the investigators, according to RECIST version 1.1.

2.2.3. Exploratory objective

To study the differences between CCI and PCI score evaluated by the center and CCI and PCI score evaluated by the central medical review.

2.2.4. Translational objective

Objective: To improve the understanding of mechanism of action of pembrolizumab and resistance mechanisms.
Hypothesis :

- Pembrolizumab has a synergistic antitumor activity with neo adjuvant and adjuvant chemotherapy plus or less bevacizumab
- Pembrolizumab as neo adjuvant and adjuvant therapy is safe when combined with standard of care in the management of Ovarian carcinoma 1st line therapy

2.3. Study Duration

The study is planned to start in Q1 2017 with respect to First Patient In (FPI) including a recruitment period of 12 months or until 90 patients are randomized in the trial.

- First patient in : Q3 2017
- Last patient randomized: Q1 2019
- End of treatment: Q1 2021
- End of follow up period: Q1 2025

3. STUDY POPULATION

3.1. Inclusion criteria

1. Be willing and able to provide written informed consent/assent for the trial.
2. Woman ≥ 18 and ≤ 75 years old on day of signing informed consent
3. Histologically confirmed diagnosis of epithelial ovarian carcinoma or fallopian tube carcinoma or primary peritoneal carcinoma with the exception of mucinous histology. Histology should be obtained by laparoscopy (or by laparotomy).
4. High grade serous or endometrioid (see appendix 1 bis)
5. Advanced FIGO stage IIIC to IV patient not able to receive primary debulking surgery for which neo adjuvant chemotherapy with carboplatin and paclitaxel is recommended (primary debulking surgery has been denied after an evaluation through laparoscopy or laparotomy). Patients with extra abdominal metastasis (FIGO 2014 Stage IV) can be included in case of completely resectable metastasis.
6. Primary debulking surgery denied and maximum surgical effort of cytoreduction with the goal of no residual disease planned at interval debulking surgery. Sugarbaker index before inclusion must be less than 30
7. Eligible for carboplatin and paclitaxel chemotherapy in accordance with local standards of care following cytoreductive surgery.
8. Interval complete surgery anticipated in a center with excellence.
9. ECOG performance status (PS) ≤ 2.
10. Life expectancy of at least 6 months,
11. Interval between diagnosis and enrolment (informed consent) ≤ 8 weeks,
12. Be willing to provide blood, and tissue from a newly obtained core or excisional biopsy of a tumor lesion. *Newly-obtained is defined as a specimen obtained up to 8 weeks (56 days) prior to initiation of treatment on Day 1.*
13. Demonstrate adequate organ function as defined in the table below, all screening labs should be performed within 7 days before randomization.
14. Adequate hematological laboratory value: Absolute neutrophil count (ANC): ≥1,500/mm^3
    - Platelets : ≥100,000/mm^3
    - Hemoglobin : ≥9 g/dL or ≥5.6 mmol/L without transfusion or EPO dependency (within 7 days of assessment)

15. Adequate renal laboratory value:
    - Serum creatinine **and** Measured or calculated creatinine clearance [a] (GFR can also be used in place of creatinine or CrCl) ≤1.5 X upper limit of normal (ULN) **OR** ≥60 mL/min for subject with creatinine levels > 1.5 X institutional ULN
    (Creatinine clearance is calculated according to Cockcroft formula or to MDRD formula for patients older than 65 years-old. Glomerular filtration rate or creatinine clearance according to MDRD formula is: GFR = 186 x (creatinine (μmol/l) x 0,0113)-1,154 x age- 0,203 x 0.742.)

16. Adequate hepatic laboratory value:
    - Serum total bilirubin: ≤ 1.5 X ULN **OR**
    - Direct bilirubin: ≤ ULN for subjects with total bilirubin levels > 1.5 ULN
    - LDH, CRP
    - AST (SGOT) and ALT (SGPT): ≤ 2.5 X ULN **OR ≤** 5 X ULN for subjects with liver metastases

17. Adequate coagulation laboratory value:
    - International Normalized Ratio (INR) or Prothrombin Time (PT) : ≤1.5 X ULN unless subject is receiving anticoagulant therapy as long as PT or PTT is within therapeutic range of intended use of anticoagulants
    - Activated Partial Thromboplastin Time (aPTT): ≤1.5 X ULN unless subject is receiving anticoagulant therapy as long as PT or PTT is within therapeutic range of intended use of anticoagulants

18. Female subject of childbearing potential should have a negative urine or serum pregnancy within 72 hours prior randomization. If the urine test is positive or cannot be confirmed as negative, a serum pregnancy test will be required.
19. Female subjects of childbearing potential should be willing to use 1 or 2 methods of birth control or be surgically sterile, or abstain from heterosexual activity for the course of the study through 4 months after the last dose of study medication and 6 months after the last dose of bevacizumab, or paclitaxel, or carboplatin. Subjects of childbearing potential are those who have not been surgically sterilized or have not been free from menses for > 1 year. (see below NB: contraception requirement)
20. Patient should be beneficiary of healthcare coverage under the social security system.

3.2. Exclusion criteria

1. Histological diagnosis of malignant tumor of non-epithelial origin (e.g. germ cell tumor, sex cord-stromal tumor) of the ovary, the fallopian tube or peritoneum or borderline tumor of the ovary (tumor of low malignant potential).

2. Patients with extra abdominal metastasis (FIGO 2014 Stage IV) not completely resectable, as e.g. multiple parenchymal lung metastases (preferably histologically proven), non resectable lymph node metastases, brain metastases.

3. Prior systemic therapy for ovarian cancer (e.g. chemotherapy, monoclonal antibody therapy, oral targeted therapy, hormonal therapy),

4. Prior radiotherapy to the abdomen or prior radiotherapy to an extra-abdominal target volume that would bear the risk of increased toxicity of chemotherapy,

5. Serious illness or concomitant non-oncological disease such as neurologic, psychiatric or infectious disease, active ulcers (gastrointestinal tract, skin) or a laboratory abnormality that may Increase the risk associated with study participation or study drug administration and in the judgment of the investigator would make the patient inappropriate for entry into the study,

6. Any contraindications for therapy with paclitaxel or carboplatin, e.g. a history of severe hypersensitivity reactions to paclitaxel or platinum-containing compounds and their excipients, or other drugs formulated with Polyoxyl 35Is currently participating and receiving study therapy or has participated in a study of an investigational agent and received study therapy or used an investigational device within 4 weeks of the first dose of treatment.

7. Diagnosis of immunodeficiency or receiving prolonged period of systemic steroid therapy or any other form of immunosuppressive therapy within 7 days prior to the first dose of trial treatment.

8. Known history of active Bacillus Tuberculosis (TB)

9. Hypersensitivity to pembrolizumab or any of its excipients.

10. Prior anti-cancer monoclonal antibody (mAb) within 4 weeks prior to study Day 1 or no recovery (i.e., ≤ Grade 1 or at baseline) from adverse events due to agents administered more than 4 weeks earlier.

11. Known additional malignancy that is progressing or requires active treatment. Exceptions include basal cell carcinoma of the skin or squamous cell carcinoma of the skin that has undergone potentially curative therapy or in situ cervical cancer.

12. Known active central nervous system (CNS) metastases and/or carcinomatous meningitis. Subjects with previously treated brain metastases may participate provided they are stable (without evidence of progression by imaging for at least four weeks prior to the first dose of trial treatment and any neurologic symptoms have returned to baseline), have no evidence of new or enlarging brain metastases, and are not using steroids for at least 7 days prior to trial treatment. This exception does not include carcinomatous meningitis which is excluded regardless of clinical stability.

13. Active autoimmune disease that has required systemic treatment in the past 2 years (i.e. with use of disease modifying agents, corticosteroids or immunosuppressive drugs). Replacement therapy (e.g., thyroxine, insulin, or physiologic corticosteroid replacement therapy for adrenal or pituitary insufficiency, etc.) is not considered a form of systemic treatment.

14. Prior therapy with an anti-PD-1, anti-PD-L1, or anti-PD-L2 agent.

15. History of Human Immunodeficiency Virus (HIV) (HIV 1/2 antibodies).

16. Active Hepatitis B (e.g., HbsAg reactive) or Hepatitis C (e.g., HCV RNA [qualitative] is detected).

17. Vaccination with a live vaccine within 30 days of planned start of study therapy.

Note: Seasonal influenza vaccines for injection are generally inactivated flu vaccines and are allowed; however intranasal influenza vaccines (e.g., Flu-Mist®) are live attenuated vaccines, and are not allowed.

18. History of (non-infectious) pneumonitis that required steroids or current pneumonitis.

19. Active infection requiring systemic therapy.

20. History or current evidence of any condition, therapy, or laboratory abnormality that might confound the results of the trial, interfere with the subject’s participation for the full duration of the trial, or is not in the best interest of the subject to participate, in the opinion of the treating investigator.

21. Psychological, familial, sociological or geographical factors potentially hampering compliance with the study protocol and follow-up schedule,

22. Psychiatric or substance abuse disorders that would interfere with cooperation with the requirements of the trial.

23. Active alcohol or drug abuse,

24. Pregnant or breastfeeding, or expecting to conceive or father children within the projected duration of the trial, starting with the pre-screening or screening visit through 4 months after the last dose of trial treatment, and six months after the last dose of bevacizumab, or paclitaxel, or carboplatin.

25. Patient unable to give their consent by their own (guardianship and curatorship)

4. STUDY TREATMENTS

4.1. Description

Two types of treatments will be used during this clinical trial:

- Standard treatment: carboplatin and paclitaxel association (± bevacizumab after IDS)
- Experimental treatment: Pembrolizumab

Standard Treatment

Carboplatin, Paclitaxel and Bevacizumab will not be supplied by the Sponsor.
Criteria for initiation and re-treatment, interruption and recommendations for dose adaptations will be done according to the SmPC.

Experimental Treatment

Pembrolizumab will be supplied by the Sponsor.
Criteria for initiation and re-treatment, interruption and recommendations for dose adaptations will be done according to the investigator brochure and to the Event of Clinical Interest (ECI) found in the trial master file.

	**Investigational product** (provided by the sponsor)
Product Name	**Pembrolizumab**
Pharmaceutical Class	humanized monoclonal antibody of the IgG4/kappa isotype
Appearance	Lyophilised powder for injection for pembrolizumab 50mg<br>Liquid solution for injection for pembrolizumab 100 mg
Storage	In the refrigerator (2 to 8°C)
Packaging and Labeling Information	Clinical supplies will be affixed with a clinical label in accordance with regulatory requirements.
Formulation / concentration	Contains 50mg or 100mg of pembrolizumab
Dilution	Details on preparation and administration of pembrolizumab (MK-3475) are provided in the Pharmacy Manual in the pharmacy's TMF.
Dose	50 mg or 100mg
Route	Intravenous
Treatment duration	After completion of six (maximum 9) cycles of combination therapy, treatment with pembrolizumab will continue as uninterrupted monotherapy, until a maximum duration of 15 months.

4.2. Treatment administration

Trial treatment should begin on the day of randomization or as close as possible to the date on which treatment is allocated/assigned (maximum 14 days after randomization date)
Trial treatment should be administered on Day 1 of each cycle after all procedures/assessments have been completed as detailed on the Trial Flow Chart (In the clinical summary). Trial treatment may be administered up to 3 days before or after the scheduled Day 1 of each cycle due to administrative reasons
The duration of one cycle corresponds to 21 days.

<u>Carboplatin and paclitaxel</u> should be taken on day 1 of each 3 weeks cycle at the dose of AUC 5 or 6 in a 15-60 minutes intravenous infusion and 175 mg/m² in a 3 hours intravenous infusion respectively. One cycle is D1 to D21 and D1C2 = D22C1
Pre-medication should be implemented according to local practices. Premedication with corticosteroids is allowed.

Bevacizumab

The planning of the administration or not of Bevacizumab will be determined at discretion of investigator, before the randomization, and could not be modified (stratification factor).

Bevacizumab administration will start in adjuvant phase only for patients stratified to receive bevacizumab:
In arm A, bevacizumab will be administrated before chemotherapy treatment.
In arm B, pembrolizumab will be administrated first, followed by bevacizumab. Then chemotherapy treatment will be administered.

The dose of bevacizumab is 15 mg/kg administered by IV infusion every 3 weeks on Days 1 with the carboplatin-paclitaxel regimen. Bevacizumab dose will be 15mg/kg. It will be delivered every 3 weeks during the adjuvant treatment and during the maintenance. Bevacizumab will be administered for a maximum of 15 months.

The initial dose of bevacizumab will be delivered over 90 (±15) minutes. If the first infusion is tolerated without infusion-associated adverse events (fever and/or chills), the second infusion may be delivered over 60 (±10) minutes. If the 60-minutes infusion is well tolerated, all subsequent infusions may be delivered over 30 (±10) minutes.

The interval between infusions must not be $<$ 10 days.

If a patient experiences an infusion-associated adverse event, she may be premedicated for the next bevacizumab infusion; however, the infusion time may not be decreased for the subsequent infusion. If the next infusion is well tolerated with premedication, the subsequent infusion time may then be decreased by 30 minutes as long as the patient continues to be premedicated. If a patient experiences a second episode of an infusion-associated adverse event with the 60-minute infusion, all subsequent doses should be given over 90 (±15) minutes. Similarly, if a patient experiences a second episode of an infusion-associated adverse event with the 30-minute infusion, all subsequent doses should be given over 60 (±10) minutes.

Pembrolizumab:

For all randomized patients in arm B, Pembrolizumab will be administered first.
For randomized patients in Arm B without bevacizumab, Pembrolizumab 200 mg will be administered as a 30 minute IV infusion every 3 weeks. Sites should make every effort to target infusion timing to be as close to 30 minutes as possible. However, given the variability of infusion pumps from site to site, a window of -5 minutes and +10 minutes is permitted (i.e., infusion time is 30 minutes: -5 min/+10 min).
For randomized patients in Arm B with bevacizumab, pembrolizumab will be administered first, followed by bevacizumab, with a minimum of 5 minutes between dosing. The initial dose of bevacizumab will be delivered over 90 (±15) minutes. If the first infusion is tolerated without infusion-associated adverse events (fever and/or chills), the second infusion may be delivered over 60 (±10) minutes. If the 60-minutes infusion is well tolerated, all subsequent infusions may be delivered over 30 (±10) minutes. If a patient experiences an infusion-associated adverse event, he or she may be premedicated for the next bevacizumab infusion; however, the infusion time may not be decreased for the subsequent infusion. If the next infusion is well tolerated with premedication, the subsequent infusion time may then be decreased by 30 minutes as long as the patient continues to be premedicated. If a patient experiences a second episode of an infusion-associated adverse event with the 60-minute infusion, all subsequent doses should be given over 90 (±15) minutes. Similarly, if a patient experiences a second episode of an infusion-associated adverse event with the 30-minute infusion, all subsequent doses should be given over 60 (±10) minutes.

The Pharmacy Manual contains specific instructions for the preparation of the pembrolizumab infusion fluid and administration of infusion solution.

No premedication will be administered.
After completion of six (maximum 9) cycles of combination therapy, treatment with pembrolizumab will continue as uninterrupted monotherapy, until a maximum duration of 15 months.

4.3. Dose adaptation guidelines

If any weight change of more than 10% or more than 10 kg is observed, the treatment dosage should be modified accordingly.

Any toxicity observed during the study treatment phase could be managed by interruption of the dose of study treatment if deemed appropriate by the Investigator. Repeat dose interruptions are allowed as required, for a maximum of 6 weeks on each occasion. If the interruption is any longer than this, the Sponsor study team must be informed and treatment restart should be discussed between the Sponsor study team and the investigator.

Study treatment must be interrupted until the patient recovers completely or the toxicity reverts to the National Cancer Institute Common Terminology Criteria for Adverse Events (NCI-CTC AE version 4.03, see appendix 6) grade 1 or less.

Treatment must be temporarily or permanently interrupted if any NCI-CTCAE grade 3 or 4 adverse event occurs which the Investigator considers to be related to administration of study treatment.

Following dose modification of the study drug, therapeutic monitoring of concomitant medications should be performed as needed in a manner consistent with the local clinical standard of care. In general, subjects should be closely monitored for side effects of all concomitant medications regardless of the path of elimination

The discontinuation/interruption for toxicity of one drug of the combination therapy during the chemotherapy period or one drug during the maintenance period does not prevent to pursue the other(s) drug(s) according to the protocol schedule.
All dose reductions and interruptions (including any missed doses), and the reasons for the reductions/interruptions are to be recorded in the eCRF.

4.3.1. Carboplatin/Paclitaxel

The adverse reaction that could occur during Carboplatin/paclitaxel treatment and management of these toxicities are listed in the SmPCs. The dose management (interruption and/or reduction) of the chemotherapies regimens should be done accordingly to local practices.
Carboplatin may be substituted with cisplatinum or oxaliplatin in case of carboplatin hypersensitivity, paclitaxel with docetaxel in case of neurotoxicity, but no other substitution are allowed.
The discontinuation/interruption for toxicity of one or 2 drugs of the 4 drugs combination therapy during the chemotherapy period or one of the 2 drugs during the maintenance period does not prevent to pursue the other(s) drug(s) according to the protocol schedule.
All dose reductions and interruptions (including any missed doses), and the reasons for the reductions/interruptions are to be recorded in the eCRF.

As a reminder, below dose adjustments for paclitaxel and carboplatin:
Paclitaxel/Carboplatin dose levels

**Dose-level**	**0**	**1**	**2**
**Paclitaxel**	175 mg/m^2	135 mg/m^2	0

**Carboplatin ***	AUC 5	AUC 4	0
**Carboplatin ***	AUC 6	AUC 5	AUC 4

Dose= target AUC in mg/mL·min*

4.3.2. Bevacizumab

No reductions in bevacizumab dose are allowed in this study. If adverse events occur that necessitate holding bevacizumab, the dose will remain unchanged once treatment resumes.

Missed doses will not be administered subsequently.

Infusion of bevacizumab should be interrupted in patients who develop dyspnea or clinically significant hypotension. Patients who experience an NCI CTCAE Grade 3 or 4 allergic reaction/hypersensitivity, adult respiratory distress syndrome, or bronchospasm (regardless of grade) will be discontinued from bevacizumab treatment.
Bevacizumab infusion rate should be slowed to ≤50% or interrupted for patients who experience any infusion-associated symptoms not specified above. If the infusion rate is interrupted, it may be resumed at ≤50% of the rate prior to the reaction after the patient's symptoms have adequately resolved and increased in 50% increments up to the full rate if well tolerated. Infusions may be restarted at the full rate during the next cycle.
Temporary suspension of bevacizumab must occur if a patient experiences a serious adverse event or a Grade 3 or 4 non-serious adverse event assessed by the investigator as related to bevacizumab. If the event resolves to Grade ≤ 1, bevacizumab may be restarted at the same dose level. If bevacizumab is delayed because of toxicity for > 6 weeks beyond when the next dose should have been given, the patient must be permanently discontinued from bevacizumab.

In cases of toxicity, please refer to the current version of the bevacizumab Smpc for guidance describing toxicities that may conduct to either temporarily or permanently suspended such as:
- Hypertension.
- Proteinuria.
- Thrombosis/embolism.
- Hemorrhage.
- Congestive heart failure.
- Surgery.
- Wound healing complications in addition to any other serious bevacizumab related toxicity (grade 3 or 4).

Bevacizumab should be temporarily withheld in the event of:
- Grade 4 febrile neutropenia.
- Grade 4 thrombocytopenia (regardless of the relationship to treatment), since these conditions are predisposing factors for an increased bleeding tendency
- Grade 4 anemia

In addition, bevacizumab treatment should be permanently discontinued in patients experiencing any of the following events:
- Reversible Posterior Leucoencephalopathy Syndrome (RPLS).
- Grade 3/4 hemorrhagic/bleeding events.
- Grade 3/4 left ventricular dysfunction (Cardiac Heart Failure).
- Grade 4 venous thromboembolism.

- Grade 4 hypertension (hypertensive crisis).
- Grade 4 non-gastrointestinal fistula.
- Grade 4 proteinuria (nephrotic syndrome).
- Any grade of central nervous system bleeding.
- Any grade of arterial thromboembolism.
- Any grade of gastrointestinal perforation.
- Any grade of tracheo-esophageal fistula.

The discontinuation/interruption for toxicity of one or 2 drugs of the 4 drugs combination therapy during the chemotherapy period or one of the 2 drugs during the maintenance period does not prevent to pursue the other(s) drug(s) according to the protocol schedule.

All dose reductions and interruptions (including any missed doses), and the reasons for the reductions/interruptions are to be recorded in the eCRF.

4.3.3. Pembrolizumab

Adverse events (AE) (both non-serious and serious) associated with pembrolizumab exposure may represent an immunologic etiology. These adverse events may occur shortly after the first dose or several months after the last dose of treatment. Pembrolizumab must be withheld for drug-related toxicities and severe or life-threatening Aes as per Table below. See supportive care part on section 3.5.1 and ECI guidance found in the trial master file for supportive care guidelines, including use of corticosteroids.

If a patient has to interrupt intake of pembrolizumab for more than 6weeks, the decision to restart treatment with pembrolizumab needs to be discussed and agreed upon between the investigator and the sponsor.

Dose Modification Guidelines for Drug-Related Adverse Events

General instructions:

1. Corticosteroid taper should be initiated upon AE improving to Grade 1 or less and continue to taper over at least 4 weeks.
2. For situations where pembrolizumab has been withheld, pembrolizumab can be resumed after AE has been reduced to Grade 1 or 0 and corticosteroid has been tapered. Pembrolizumab should be permanently discontinued if AE does not resolve within 12 weeks of last dose or corticosteroids cannot be reduced to ≤10 mg prednisone or equivalent per day within 12 weeks.
3. For severe and life-threatening irAEs, IV corticosteroid should be initiated first followed by oral steroid. Other immunosuppressive treatment should be initiated if irAEs cannot be controlled by corticosteroids.

Immune-related Aes	Toxicity grade or conditions (CTCAEv4.0)	Action taken to pembrolizumab	irAE management with corticosteroid and/or other therapies	Monitor and follow-up
Pneumonitis	Grade 2	Withhold	• Administer corticosteroids (initial dose of 1-2 mg/kg prednisone or equivalent) followed by taper	• Monitor participants for signs and symptoms of pneumonitis<br>• Evaluate participants with suspected pneumonitis with radiographic imaging and initiate corticosteroid treatment<br>• Add prophylactic antibiotics for opportunistic infections
	Grade 3 or 4, or recurrent Grade 2	Permanently discontinue		
Diarrhea / Colitis	Grade 2 or 3	Withhold	• Administer corticosteroids (initial dose of 1-2 mg/kg prednisone or equivalent) followed by taper	• Monitor participants for signs and symptoms of enterocolitis (ie, diarrhea, abdominal pain, blood or mucus in stool with or

	Grade 4	Permanently discontinue		without fever) and of bowel perforation (ie, peritoneal signs and ileus).<br>• Participants with ≥ Grade 2 diarrhea suspecting colitis should consider GI consultation and performing endoscopy to rule out colitis.<br>• Participants with diarrhea/colitis should be advised to drink liberal quantities of clear fluids. If sufficient oral fluid intake is not feasible, fluid and electrolytes should be substituted via IV infusion.
AST / ALT elevation or Increased bilirubin	Grade 2	Withhold	• Administer corticosteroids (initial dose of 0.5- 1 mg/kg prednisone or equivalent) followed by taper	• Monitor with liver function tests (consider weekly or more frequently until liver enzyme value returned to baseline or is stable
	Grade 3 or 4	Permanently discontinue	• Administer corticosteroids (initial dose of 1-2 mg/kg prednisone or equivalent) followed by taper	
Type 1 diabetes mellitus (T1DM) or Hyperglycemia	Newly onset T1DM or Grade 3 or 4 hyperglycemia associated with evidence of β-cell failure	Withhold	• Initiate insulin replacement therapy for participants with T1DM<br>• Administer anti-hyperglycemic in participants with hyperglycemia	• Monitor participants for hyperglycemia or other signs and symptoms of diabetes.
Hypophysitis	Grade 2	Withhold	• Administer corticosteroids and initiate hormonal replacements as clinically indicated.	• Monitor for signs and symptoms of hypophysitis (including hypopituitarism and adrenal insufficiency)
	Grade 3 or 4	Withhold or permanently discontinue[1]		
Hyperthyroidism	Grade 2	Continue	• Treat with non-selective beta-blockers (eg, propranolol) or thionamides as appropriate	• Monitor for signs and symptoms of thyroid disorders.
	Grade 3 or 4	Withhold or permanently discontinue[1]		
Hypothyroidism	Grade 2-4	Continue	• Initiate thyroid replacement hormones (eg, levothyroxine or liothyroinine) per standard of care	• Monitor for signs and symptoms of thyroid disorders.
Nephritis and Renal dysfunction	Grade 2	Withhold	• Administer corticosteroids (prednisone 1-2 mg/kg or equivalent) followed by taper.<br>•	• Monitor changes of renal function
	Grade 3 or 4	Permanently discontinue		

Myocarditis	Grade 1 or 2	Withhold	• Based on severity of AE administer corticosteroids	• Ensure adequate evaluation to confirm etiology and/or exclude other causes
	Grade 3 or 4	Permanently discontinue		
All other immune-related Aes	Intolerable/ persistent Grade 2	Withhold	• Based on type and severity of AE administer corticosteroids	• Ensure adequate evaluation to confirm etiology and/or exclude other causes
	Grade 3	Withhold or discontinue based on the type of event. Events that require discontinuation include and not limited to: Gullain-Barre Syndrome, encephalitis		
	Grade 4 or recurrent Grade 3	Permanently discontinue		
1. Withhold or permanently discontinue pembrolizumab is at the discretion of the investigator or treating physician.<br>**NOTE:**<br>For participants with Grade 3 or 4 immune-related endocrinopathy where withhold of pembrolizumab is required, pembrolizumab may be resumed when AE resolves to ≤ Grade 2 and is controlled with hormonal replacement therapy or achieved metabolic control (in case of T1DM).				

<u>MANAGEMENT OF PEMBROLIZUMAB SPECIFIC ADVERSE EVENTS</u>

Toxicities associated or possibly associated with Pembrolizumab treatment should be managed according to standard medical practice. Additional tests, such as autoimmune serology or biopsies, should be used to determine a possible immunogenic etiology.

Infusion-Related (ir) Reactions

Pembrolizumab may cause severe or life threatening infusion-reactions including severe hypersensitivity or anaphylaxis. Signs and symptoms usually develop during or shortly after drug infusion and generally resolve completely within 24 hours of completion of infusion. Dose modification and toxicity management guidelines on pembrolizumab associated infusion reaction are provided in Table below

NCI CTCAE Grade	Treatment	Premedication at Subsequent Dosing
**Grade 1**<br>Mild reaction; infusion interruption not indicated; intervention not indicated	Increase monitoring of vital signs as medically indicated until the participant is deemed medically stable in the opinion of the investigator.	None
**Grade 2**<br>Requires therapy or infusion interruption but responds promptly to symptomatic treatment (e.g., antihistamines, NSAIDs, narcotics, IV fluids); prophylactic medications indicated for ≤24 hrs	**Stop Infusion.**<br>Additional appropriate medical therapy may include but is not limited to:<br>IV fluids<br>Antihistamines<br>NSAIDs<br>Acetaminophen<br>Narcotics<br>Increase monitoring of vital signs as medically indicated until the participant is deemed medically stable in the opinion of the investigator.<br>If symptoms resolve within 1 hour of stopping drug infusion, the infusion may be restarted at 50% of the original infusion rate (e.g. from 100 mL/hr to 50 mL/hr). Otherwise dosing will be held until symptoms resolve and the participant should be premedicated for the next scheduled dose.<br>**Participants who develop Grade 2 toxicity despite adequate premedication should be permanently discontinued from further study drug treatment**	Participant may be premedicated 1.5h (± 30 minutes) prior to infusion of _____ with:<br>Diphenhydramine 50 mg po (or equivalent dose of antihistamine).<br>Acetaminophen 500-1000 mg po (or equivalent dose of analgesic).
**Grades 3 or 4**<br>Grade 3:<br>Prolonged (i.e., not rapidly responsive to symptomatic medication and/or brief interruption of infusion); recurrence of symptoms following initial improvement; hospitalization indicated for other clinical sequelae (e.g., renal impairment, pulmonary infiltrates)<br>Grade 4:<br>Life-threatening; pressor or ventilatory support indicated	**Stop Infusion.**<br>Additional appropriate medical therapy may include but is not limited to:<br>Epinephrine**<br>IV fluids<br>Antihistamines<br>NSAIDs<br>Acetaminophen<br>Narcotics<br>Oxygen<br>Pressors<br>Corticosteroids<br>Increase monitoring of vital signs as medically indicated until the participant is deemed medically stable in the opinion of the investigator.<br>Hospitalization may be indicated.<br>**In cases of anaphylaxis, epinephrine should be used immediately.<br>**Participant is permanently discontinued from further study drug treatment.**	No subsequent dosing
Appropriate resuscitation equipment should be available at the bedside and a physician readily available during the period of drug administration.<br>For further information, please refer to the Common Terminology Criteria for Adverse Events v4.03 (CTCAE)		

Gastrointestinal (GI) toxicity

Autoimmune colitis has been associated with the administration of pembrolizumab. Diarrhea (defined as either first watery stool or an increase in frequency of 50% above baseline with urgency or nocturnal bowel movement or bloody stool) should be further evaluated, and infectious or alternate etiologies should be ruled out. Patients should be advised to inform the investigator if any diarrhea occurs, even if it is mild. If the event is of significant duration or magnitude or is associated with signs of systemic inflammation or acute phase reactants (e.g., increased CRP or platelet count or bandemia), it is recommended that sigmoidoscopy (or colonoscopy, if appropriate) with colonic biopsy with three to five specimens for standard paraffin block be performed. If possible, one or two biopsy specimens should be snap frozen and stored. Tests should also be performed for WBCs and for stool calprotectin.

All patients with confirmed colitis should also have an ophthalmological examination, including a slitlamp examination, to rule out uveitis.Study treatment should be interrupted for patients who develop Grade ≥ 2 colitis. Patients with colitis should also discontinue any nonsteroidal anti-inflammatory medications or any other medications known to exacerbate colitis symptoms.

Investigators should use their clinical judgment as to whether corticosteroids are necessary to treat colitis associated with pembrolizumab therapy and what dose should be used.

Treatment may be restarted following clearance of colitis confirmed by sigmoidoscopy (or colonoscopy, if appropriate) with colonic biopsy. In addition, if the patient is being managed with corticosteroids, treatment should not be restarted until the steroids have been tapered off to a prednisone dose ≤10 mg/day. Patients who resume treatment should be monitored closely for sign of renewed diarrhea.

Hepatic events

Autoimmune hepatitis has been associated with the administration of pembrolizumab. Safety in patients with ALT or AST > 2.5 x ULN or, if due to liver metastases, with ALT or AST > 5.0 x ULN has not been established.

Eligible patients must have adequate liver function, as manifested by measurements of total bilirubin and hepatic transaminase, and liver function will be monitored throughout study treatment.

Patients with known liver disease that could increase susceptibility to or exacerbate the impact of any potential hepatotoxicity of study treatment will be excluded from the study.

While on this study, patients presenting with right upper-quadrant abdominal pain and/or unexplained nausea or vomiting should have LFTs (Liver Function Tests) performed immediately and reviewed before administration of the next dose of study drug.

For patients with no hepatic metastases at baseline, if LFTs increase to either ≥ 3-fold from baseline or levels reaching NCI CTCAE Grade 2, LFTs should be monitored weekly until returning to baseline. Treating physicians should discuss with the Trial Manager prior to any additional study drug administration. Pembrolizumab treatment should not resume until LFTs have resolved to Grade ≤ 1. Neoplastic, concurrent medications, viral hepatitis, and toxic etiologies should be considered and addressed, as appropriate. Imaging of the liver, gall bladder, and biliary tree should be performed to rule out neoplastic or other causes for the increased LFTs. Anti-nuclear antibody, perinuclear anti-neutrophil cytoplasmic antibody, anti-LKM, and anti-smooth muscle antibody tests should be performed if an autoimmune etiology is considered.

For patients with no hepatic metastases at baseline who develop Grade ≥ 3 hepatotoxicity during study treatment (ALT and/or AST of > 10 x ULN or total bilirubin of > 5 x ULN), study treatment should be permanently interrupted. Consultation with a hepatologist is appropriate for a suspected liver immune-related adverse event, and a biopsy is strongly recommended prior to the initiation of steroids. As guidance, prior experience suggests that one could use high-dose IV methylprednisolone for 24�48 hours, followed by an oral steroid taper with dexamethasone in a dosage of 4 mg every 4 hours or prednisone at 40�60 mg/day tapered over not less than 30 days. Liver function blood tests should be followed every other day until they begin to drop, then weekly until results are normal or return to baseline. If LFT results do not decrease within 48 hours after initiation of systemic steroids, oral mycophenolate mofetil 500 mg every 12 hours should be considered.

Further elevations of LFT results in patients with documented liver metastasis and elevated LFT results at baseline may not require dose interruptions if there are no progressive changes in the ALT and/or AST (less than a doubling) and if there are no progressive elevations in total bilirubin or INR.

Treatment may be restarted with the approval of the Trial Manager following return of LFT results to the patient's baseline or to AST and ALT ≤3 x ULN and total bilirubin ≤ 1.5 x ULN. In addition, if the patient is being managed with corticosteroids, treatment should not be restarted until the steroids have been tapered to a prednisone dose (or dose equivalent) of ≤ 10 mg/day.

Dermatologic toxicity

The majority of cases of rash were mild in severity and self-limited, with or without pruritus.
A dermatologist should evaluate persistent and/or severe rash or pruritus. A biopsy should be performed unless contraindicated, and if possible, photographs of the rash should also be obtained and submitted to the Sponsor. Low-grade rash and pruritus irAEs have been treated with symptomatic therapy (e.g antihistamines). Topical or parenteral corticosteroids may be required for more severe symptoms. Any considerations for either study treatment hold or the use of systemic corticosteroids for rash and/or pruritus should be discussed with the Trial Manager.

Ocular events

Patients in the study are encouraged to maintain eye hydration, generally through the use of moisturizing eye drops. An ophthalmologist should evaluate visual complaints with an examination of the conjunctiva, anterior and posterior chambers, and retina; visual field testing and an electroretinogram should also be performed. Uveitis or episcleritis may be treated with topical corticosteroid eye drops.
Pembrolizumab should be permanently discontinued for immune-mediated ocular disease that is unresponsive to local immunosuppressive therapy.

Endocrine events

Hypothyroidism events assessed by the investigator as related to pembrolizumab had been reported. Patients with unexplained symptoms such as fatigue, myalgias, impotence, mental status changes, or constipation should be investigated for the presence of thyroid, pituitary, or adrenal endocrinopathies, as well as for hyponatremia or hyperkalemia. An endocrinologist should be consulted if an endocrinopathy is suspected. Thyroid-stimulating hormone and free T4 levels should be obtained to determine whether thyroid abnormalities are present. Thyroid-stimulating hormone, prolactin, and a morning cortisol level will help to differentiate primary adrenal insufficiency from primary pituitary insufficiency.
Management of hypothyroidism should follow accepted clinical practice guidelines. Study treatment interruption is usually not required unless patients are symptomatic. In symptomatic patients, pembrolizumab should be held until symptoms have resolved to baseline grade or better.
Autoimmune diabetes mellitus has been observed with pembrolizumab. Patients with persistent hyperglycemia should be evaluated for the potential of immune-mediated pancreatic endocrine insufficiency with measurement of serum C-peptide and islet-cell or anti-GAD autoantibodies. Consultation with an endocrinologist is appropriate in these settings.

Pulmonary toxicity

Dyspnea, cough, fatigue, hypoxia, pneumonitis, and pulmonary infiltrates have been associated with the administration of pembrolizumab, and have primarily been observed in patients with underlying NSCLC.
Mild to moderate organizing pneumonia and subacute ground glass opacities have been associated with pembrolizumab. Appropriate workup for pulmonary adverse events should include the following as appropriate, as well as ruling out alternative causes (e.g., pneumonia/infection lymphangitic carcinomatosis, infection, heart failure, or chronic obstructive pulmonary disease, or pulmonary hypertension):

- Measurement of oxygen saturation (i.e., arterial blood gas)
- High-resolution CT scan of the chest
- Bronchoscopy with bronchoalveolar lavage and biopsy
- Pulmonary function tests (with DLCO)

Pulmonary function testing and CT scan with a pulmonary embolism protocol may also be helpful in the diagnostic evaluation. Patients with worsening ground glass opacities in the absence of hypoxia or dyspnea may benefit from the initiation of prednisone 10 mg per day or equivalent. For patients with clinical symptoms such as acute hypoxia, treatment should include administration of high-dose IV methylprednisolone for 24[illegible]48 hours, followed by an oral steroid taper with prednisone at 40/60 mg/day (over a period of 2 weeks) and/or oxygen when indicated. Consultation with a pulmonologist is appropriate for a suspected lung irAEs, and a biopsy should be performed unless contraindicated prior to the administration of steroids.
Patients with known history of idiopathic pulmonary fibrosis, pneumonitis, risk of pulmonary toxicity, or evidence of active pneumonitis on screening chest CT scan will be excluded from the study.

Pancreatic disorders

Symptoms of abdominal pain associated with elevations of amylase and lipase, suggestive of pancreatitis, have been associated with the administration of other immuno-modulatory agents. The differential diagnosis of acute abdominal pain should include pancreatitis. Appropriate work-up should include an evaluation for obstruction, as well as serum amylase and lipase tests.

Neurologic disorders

Myasthenia gravis and Guillain-Barre syndrome have been observed with single agent pembrolizumab. Patients may present with signs and symptoms of sensory and/or motor neuropathy. Diagnostic work-up is essential for an accurate characterization to differentiate between alternate etiologies.

4.4. Study treatments compliance and accountability

Carboplatin, Paclitaxel and bevacizumab

Preparation sheets of used carboplatin and paclitaxel vials should be available for accountability.

Pembrolizumab

The Investigator, the Hospital Pharmacist, or other personnel allowed to store and dispense the drugs will be responsible for ensuring that Pembrolizumab is securely maintained and in accordance with the applicable regulatory requirements.

The Investigational Medical Product shall be dispensed in accordance with the Investigator's prescription and it is the Investigator's responsibility (or designee) to ensure that an accurate record of the study drug administered is maintained. These records include the date at which the IMP is received, dispensed to patients, and destroyed.

4.5. Concomitant medications and treatments

Concomitant medications, or therapy to provide adequate care, may be given as clinically necessary.

Non-oncological therapies that are in use at the beginning of the trial (baseline), start or change during the treatment phase are allowed.

Medications or vaccinations specifically prohibited in the exclusion criteria are not allowed during the ongoing trial. If there is a clinical indication for one of these or other medications or vaccinations specifically prohibited during the trial, discontinuation from trial therapy or vaccination may be required. The investigator should discuss any questions regarding this with the Sponsor team. The final decision on any supportive therapy or vaccination rests with the investigator and/or the subject's primary physician.

Contraindication, special warnings and precautions for use are listed in SmPCs for carboplatin, paclitaxel and bevacizumab, and in the Investigator's Brochure for the Pembrolizumab.

4.5.1. Supportive care

Patients should receive full supportive care, including transfusions of blood and blood products, antibiotics, antiemetics, etc., when appropriate. Patients who experience infusion-associated symptoms may be treated symptomatically with acetaminophen, ibuprofen, diphenhydramine, and/or ranitidine or another H2 receptor antagonist, as per standard practice. Serious infusion-associated events manifested by dyspnea, hypotension, wheezing, bronchospasm, tachycardia, reduced oxygen saturation, or respiratory distress should be managed with supportive therapies as clinically indicated (e.g., supplemental oxygen and b2-adrenergic agonists)

Systemic corticosteroids and TNF-a inhibitors may attenuate potential beneficial immunologic effects of treatment with pembrolizumab but may be administered at the discretion of the treating physician. If feasible, alternatives to corticosteroids should be considered. Premedication may be administered for the carboplatin-paclitaxel regimen. The use of inhaled corticosteroids and mineralocorticoids (e.g., fludrocortisone) for patients with orthostatic hypotension or adrenocortical insufficiency is allowed. Megestrol administered as an appetite stimulant is acceptable while the patient is enrolled in the study. Mineralocorticoids for orthostatic hypotension or adrenalcortical insufficiency are acceptable.

The use of granulocyte growth factors from the outset, as a preventive measure in the first cycle is not justified under international guidelines. Administration at Cycle ≥ 2 is possible at the discretion of the treating physician. The subcutaneous administration of glycosylated G-CSF (lenograstim) at 150 µg/m2/d from D5 to D10 is recommended for secondary prophylaxis for febrile neutropenia grade 3-4. Once started, the lenograstim (Granocyte®) is used for all subsequent cycles. This secondary prophylaxis can be offered after a grade 4 neutropenia lasting more than 7 days, or an extension greater than 7 days due to neutropenia <1500 / mm^3. If feasible, alternatives should be considered (decrease of chemotherapy dose).

Anemia should be treated by supportive measures, no dose reductions of pembrolizumab, paclitaxel or carboplatin ± Bevacizumab are recommended.

Subjects should receive appropriate supportive care measures as deemed necessary by the treating investigator. Suggested supportive care measures for the management of adverse events with potential immunologic etiology are outlined below and in greater detail in the Event of Clinical Interest (ECI) guidance document. Where appropriate, these guidelines include the use of oral or intravenous treatment with corticosteroids as well as additional anti-inflammatory agents if symptoms do not improve with administration of corticosteroids. Note that several courses of steroid tapering may be necessary as symptoms may worsen when the steroid dose is decreased. For each disorder, attempts should be made to rule out other causes such as metastatic disease or bacterial or viral infection, which might require additional supportive care. The treatment guidelines are intended to be applied when the investigator determines the events to be related to pembrolizumab.

Note: if after the evaluation the event is determined not to be related, the investigator is instructed to follow the ECI reporting guidance but does not need to follow the treatment guidance (as outlined in the ECI guidance document). Refer to 4.3 for dose modification.

It may be necessary to perform conditional procedures such as bronchoscopy, endoscopy, or skin photography as part of evaluation of the event. Suggested conditional procedures, as appropriate, can be found in the ECI guidance document.

4.5.2. Acceptable Concomitant Medications

All treatments that the investigator considers necessary for a subject's welfare may be administered at the discretion of the investigator in keeping with the community standards of medical care. All concomitant medication will be recorded on the case report form (eCRF) including all prescription, over-the-counter (OTC), herbal supplements, and IV medications and fluids. If changes occur during the trial period, documentation of drug dosage, frequency, route, and date may also be included on the eCRF.
All concomitant medications received within 28 days before the first dose of trial treatment and 30 days after the last dose of trial treatment should be recorded. Concomitant medications administered after 30 days after the last dose of trial treatment should be recorded for SAEs and ECIs as defined in Section 14.3.
Patients who require therapeutic anticoagulation with low molecular weight heparin (e.g., enoxaparin and tinzaparin) or fondaparinux at study entry will be eligible for enrollment. In addition, patients requiring therapeutic anticoagulation with these agents during study participation will be allowed to remain on study therapy.

4.5.3. Prohibited Concomitant Medications

Subjects are prohibited from receiving the following therapies during the Screening and Treatment Phase (including retreatment for post-complete response relapse) of this trial:

- Immunotherapy not specified in this protocol
- Chemotherapy not specified in this protocol
- Investigational agents other than pembrolizumab
- Radiation therapy

Note: Radiation therapy to a symptomatic solitary lesion or to the brain may be allowed at the investigator's discretion.

Traditional herbal medicines should not be administered because the ingredients of many herbal medicines are not fully studied and their use may result in unanticipated drug-drug interactions that may cause or confound assessment of toxicity.

- Live vaccines within 30 days prior to the first dose of trial treatment and while participating in the trial. Examples of live vaccines include, but are not limited to, the following: measles, mumps, rubella, varicella/zoster, yellow fever, rabies, BCG, and typhoid vaccine.
- Systemic glucocorticoids for any purpose other than to modulate symptoms from an event of clinical interest of suspected immunologic etiology. The use of physiologic doses of corticosteroids may be approved after consultation with the Sponsor.
- Patients are not allowed to receive immunostimulatory agents, including but not limited to IFN-[illegible], IFN-[illegible], or IL-2, during the entire study. These agents, in combination with Pembrolizumab, could potentially increase the risk for autoimmune conditions. In addition, all patients (including those who discontinue the study early) should not receive other immunostimulatory agents for 10 weeks after the last dose of pembrolizumab.
- Patients should also not receive immunosuppressive medications, including but not limited to cyclophosphamide, azathioprine, methotrexate, and thalidomide. These agents could potentially alter the activity and the safety of pembrolizumab.

Subjects who, in the assessment by the investigator, require the use of any of the aforementioned treatments for clinical management should be removed from the trial. Subjects may receive other medications that the investigator deems to be medically necessary.
The Exclusion Criteria describes other medications which are prohibited in this trial.
There are no prohibited therapies during the Post-Treatment Follow-up Phase.

4.5.4. Diet/Activity/Other Considerations

<u>The appropriate interval between the last dose of bevacizumab and major surgery is 8 weeks. Re-initiation of bevacizumab following surgery should not occur for 28 days and until wounds have fully healed. Re-initiation of bevacizumab after surgery requires documented approval from the Trial Manager.</u>

<u>Diet</u>
Subjects should maintain a normal diet unless modifications are required to manage an AE such as diarrhea, nausea or vomiting.

<u>Contraception</u>
Pembrolizumab may have adverse effects on a fetus in utero.
Female subjects will be considered of non-reproductive potential if they are either:

(1) postmenopausal (defined as at least 12 months with no menses without an alternative medical cause; in women < 45 years of age a high follicle stimulating hormone (FSH) level in the postmenopausal range may be used to confirm a post-menopausal state in women not using hormonal contraception or hormonal replacement therapy. In the absence of 12 months of amenorrhea, a single FSH measurement is insufficient.);

OR

(2) have had a hysterectomy and/or bilateral oophorectomy, bilateral salpingectomy or bilateral tubal ligation/occlusion, at least 6 weeks prior to screening;

OR

(3) has a congenital or acquired condition that prevents childbearing.

Female and male subjects of reproductive potential must agree to avoid becoming pregnant or impregnating a partner, respectively, while receiving study drug and for 120 days after the last dose of study drug by complying with one of the following:
(a) practice abstinence from heterosexual activity;
OR
(b) use (or have their partner use) acceptable contraception during heterosexual activity.

Acceptable methods of contraception are:
Single method (one of the following is acceptable):
• intrauterine device (IUD)
• vasectomy of a female subject's male partner
• contraceptive rod implanted into the skin

Combination method (requires use of two of the following):
• diaphragm with spermicide (cannot be used in conjunction with cervical cap/spermicide)
• cervical cap with spermicide (nulliparous women only)
• contraceptive sponge (nulliparous women only)
• male condom or female condom (cannot be used together)
• hormonal contraceptive: oral contraceptive pill (estrogen/progestin pill or progestin-only pill), contraceptive skin patch, vaginal contraceptive ring, or subcutaneous contraceptive injection

❖ Abstinence (relative to heterosexual activity) can be used as the sole method of contraception if it is consistently employed as the subject's preferred and usual lifestyle and if considered acceptable by local regulatory agencies and ERCs/IRBs. Periodic abstinence (e.g., calendar, ovulation, sympto-thermal, post-ovulation methods, etc.) and withdrawal are not acceptable methods of contraception.

❖ If a contraceptive method listed above is restricted by local regulations/guidelines, then it does not qualify as an acceptable method of contraception for subjects participating at sites in this country/region.

Subjects should be informed that taking the study medication may involve unknown risks to the fetus (unborn baby) if pregnancy were to occur during the study. In order to participate in the study subjects of childbearing potential must adhere to the contraception requirement (described above) from the day of study medication initiation (or 14 days prior to the initiation of study medication for oral contraception) throughout the study period up to 4 months after the last dose of trial therapy, and six months after the last dose of bevacizumab, or paclitaxel, or carboplatin. If there is any question that a subject of childbearing potential will not reliably comply with the requirements for contraception, that subject should not be entered into the study.

Use in Pregnancy

If a subject inadvertently becomes pregnant while on treatment with pembrolizumab, the subject will immediately be removed from the study. The site will contact the subject at least monthly and document the subject's status until the pregnancy has been completed or terminated. The outcome of the pregnancy will be reported to the Sponsor without delay and within 24 hours to the Sponsor if the outcome is a serious adverse experience (e.g., death, abortion, congenital anomaly, or other disabling or life-threatening complication to the mother or newborn).

The study investigator will make every effort to obtain permission to follow the outcome of the pregnancy and report the condition of the fetus or newborn to the Sponsor.

Use in Nursing Women
It is unknown whether pembrolizumab is excreted in human milk. Since many drugs are excreted in human milk, and because of the potential for serious adverse reactions in the nursing infant, subjects who are breast-feeding are not eligible for enrollment.

4.6. Treatment discontinuation

4.6.1. Discontinuation of treatment (in both arm)

Patient may be discontinued from treatment in the following situations:

- Objective radiological disease progression according to RECIST 1.1 (see Appendix 5) criteria, unless in the Investigator's opinion patient is benefiting from treatment and patient does not meet any other discontinuation criteria as outlined in this section.
- Unacceptable Adverse Event.
- Intercurrent illness which prevents further treatment
- Investigator decision
- Severe non-compliance to study protocol.
- Patient decision. The patient is at any time free to discontinue treatment, without prejudice to further treatment.
- Pregnancy

Patients who decide to discontinue investigational product will always be asked about the reason(s) of discontinuation and the presence of any adverse events. If possible, they will be seen and assessed by the investigator(s). (

By discontinuing from treatment, the patient is not withdrawing from the study. Patients should be followed for progression (if discontinuation in the absence of progression), PFS2 and OS following treatment discontinuation as per the protocol schedule. If a patient is withdrawn from study see the corresponding paragraph below

Any patient discontinuing investigational product should be seen before the introduction of a new treatment and no later than 30 days post discontinuation for the evaluations outlined in the study schedule. The patient's tumor status should be assessed clinically and, if appropriate, disease progression should be confirmed by radiological assessment. After discontinuation of study medication, the Investigator will perform the best possible observation(s), test(s) and evaluation(s) as well as give appropriate medication and all possible measures for the safety of the patient. In addition, they will record on the eCRF the date of discontinuation, the reasons, manifestation and treatment at the time of discontinuation. Patients will be required to attend the treatment discontinuation visit. The patient should return all study medication.

After discontinuation of the study medication at any point in the study, all ongoing Aes or SAEs must be followed until resolution unless, in the Investigator's opinion the condition is unlikely to resolve due to the patients underlying disease, or the patient is lost to follow up. All new Aes and SAEs occurring during the 30 calendar days after the last dose of study medication must be reported (if SAEs, they must be reported to ARCAGY-GINECO for Safety within 24 hours as described in Section 14 and followed to resolution as above. Patients should be seen at least 30 days after discontinuing study medication to collect and / or complete AE information. Any untoward event occurring subsequent to the 30-day follow-up AE reporting period that the investigator assesses as possibly related to the study medication should also be reported as an AE.

Any patient who has not yet shown objective radiological disease progression at withdrawal from IP should continue to be followed as per RECIST as detailed in section 12.

Details of first and subsequent therapies for cancer and/or details of surgery for the treatment of the cancer, after discontinuation of treatment, will be collected. Reasons for starting subsequent anti-cancer therapies will be collected and included in the exploratory assessments of OS.

All patients must be followed for survival, up to the final analysis.

The maximum duration of the study will be 24 months treatment (from the randomization to the last dose of experimental product)

4.6.2. Withdrawal from study

Only patients who withdraw consent prematurely terminated the study.

Patients are at any time free to withdraw from study (product and assessments), without prejudice to further treatment (withdrawal of consent). Such patients will always be asked about the reason(s) and the presence of any adverse events. If possible, they will be seen and assessed by an investigator.

The status of ongoing, withdrawn (from the study) and "lost to follow-up" patients at the time of an overall survival analysis should be obtained by the site personnel by checking the patient notes, hospital records, contacting the patient's general practitioner and checking publicly available death registries. In the event that the patient has actively withdrawn consent to the processing of their personal data the vital status of the patient can be obtained by site personnel from publicly available resources where it is possible to do so under applicable local laws. Withdrawn patients will not be replaced.

If a patient withdraws consent, she will be specifically asked if they are withdrawing consent to:

- Further participation in the study including any further follow up (e.g., survival calls)
- The use of their study generated data
- The use of any samples (see section 16)

5. PATIENT ENROLMENT

The investigator or designee staff will have to proceed to the following information/procedures during the inclusion visit:

- Inform the patient of the treatments, the objectives and the design of the study, answer to questions and sign the informed consent form with her.
- Check the selection criteria list and perform the following exams:

ASSESSMENTS / PROCEDURES AT INCLUSION	TIMING
Informed consent<br>Inclusion and non-inclusion criteria verification<br>**Mandatory blood samples for translational research**<br>**Mandatory tumor block from initial laparoscopy collection for translational research**	Within 28 days prior randomization<br><br>*Within 7 days prior randomization
**Demographics**	
**Relevant concomitant treatments and symptoms**<br>Assessment of baseline signs and symptoms (concomitant disease)<br>Prior/concomitant therapies (within 4 weeks prior study entry)<br>Medical history	
**Complete physical examination**<br>(height, weight, Performance status (ECOG), blood pressure	

***Pregnancy tests** (if applicable) urine or serum<br>Serum or urine pregnancy tests<br>If urinary test is positive, a serum test should be performed	Within 28 days prior randomization<br><br>*Within 7 days prior randomization
**Cardiac function assessment**<br>ECG	
***Tumor marker**<br>CA-125	
**Completion of Sugarbaker index (see appendix 3)**	
**Tumour assessment**<br>*Thoracic-abdominal-pelvic CT scan; possibility to perform MRI +Chest X-Ray if CT-Scan not feasible*	
**Mandatory blood samples for translational research**<br>**Mandatory tumor block from initial laparoscopy collection for translational research**	
***Laboratory tests**<br>*Haematology*: haemoglobin, white blood cells (WBC), Absolut neutrophil Count (ANC), platelet<br>*Coagulation panel*: INR or PT, aPTT,<br>*Chemistry*: Albumin, creatinin, [1]creatinin clearance, ASAT, ALAT, LDH, alkaline phosphatase, total bilirubin, urine analyses, TSH, T3, T4	

[1] Creatinine clearance is calculated according to Cockcroft formula or to MDRD formula for patients older than 65years-old. Glomerular filtration rate or creatinine clearance according to MDRD formula is: GFR = 186 x (creatinine (µmol/l) x 0,0113)$^{-1,154}$ x age$^{-0,203}$ x 0.742.

After the screening visit, if the patient is eligible, she will be randomised to be allocated to a treatment arm. Randomization must be done as close as possible to the start of treatment (maximum 14 days before first treatment intake). It may be performed on the day of visit 1, but before application of the first dose of study treatment.

Randomization will be done via the e-CRF. The patient's study ID and assigned treatment regimen will be e-mailed to the investigator.

During the course of the study, all patients entering the study must be evaluated according to the schedule summarized in General Study Flow-chart.

6. ON-NEO-ADJUVANT-TREATMENT PERIOD ASSESSMENTS

ASSESSMENTS / PROCEDURES DURING ON-NEO-ADJUVANT-TREATMENT PERIOD	TIMING	
	Day -3 to day 1 of each cycle	Day -7 to day 1 of cycle 3
**Complete physical examination**<br>(weight, Performance status (ECOG), blood pressure	X	
**Change in concomitant treatments and symptoms**<br>AE, concomitant review	X	
**Tumor marker**<br>CA-125	X	
**Laboratory tests**<br>*Haematology*: haemoglobin, white blood cells, (WBC), Absolut neutrophil Count (ANC), platelet<br>*Coagulation panel*: INR or PT, aPTT,<br>*Chemistry*:, creatinin, creatin clearance, ASAT, ALAT, alkaline phosphatase, total bilirubin,<br>*Hormonology (only for patients in arm B):* TSH, T3, T4	X	
**RECIST Tumour assessment**		X

Thoracic-abdominal-pelvic CT scan; possibility to perform MRI +Chest X-Ray if CT-Scan not feasible		
**Mandatory blood samples for translational research at C2 and C4**		

7. END OF NEO-ADJUVANT-TREATMENT ASSESSMENTS

ASSESSMENTS / PROCEDURES DURING END OF ON-NEO-ADJUVANT-TREATMENT VISIT	TIMING<br>3 weeks +/- 3 days after chemotherapy administration of last cycle
**Complete physical examination**<br>(weight, Performance status (ECOG), blood pressure	X
**Change in concomitant treatments and symptoms**<br>Signs and symptoms, AE, concomitant review	X
**Tumor marker**<br>(serum CA-125)	X
**Laboratory tests**<br>*Haematology*: haemoglobin, white blood cells (WBC), Absolut neutrophil Count (ANC), platelet<br>*Coagulation panel*: INR or PT, aPTT,<br>*Chemistry*: creatinine, creatin clearance, ASAT, ALAT, alkaline phosphatase, total bilirubin,<br>*Hormonology (only for patients in arm B)*: TSH, T3, T4	X
**RECIST Tumour assessment**<br>*Thoracic-abdominal-pelvic CT scan; possibility to perform MRI +Chest X-Ray if CT-Scan not feasible*	X

8. INTERVAL DEBLUKING SURGERY ASSESSMENTS

IDS will be performed 3 to 4 weeks after the last chemotherapy administration.

ASSESSMENTS / PROCEDURES DURING INTERVAL DEBLUKING SURGERY	TIMING		
	Before start of debluking	During debluking	After debluking
**Completion of Sugarbaker index** (PCI: Peritoneal Cancer Index) before any surgical procedure (see appendix 3)	X		X
**Completeness of Cytoreduction Index (CCI) (see appendix 3)**			X
**Completion of a report on procedures performed**		X	
**List and grading of per-operative complications according to Clavien-Dindo classification (see appendix 4)**			X
**Mandatory collection of tumor samples from ovary and metastasis**		X	

9. POST OPERATIVE ASSESSMENTS

ASSESSMENTS / PROCEDURES DURING POST-OPERATIVE VISIT	TIMING<br>4 weeks ± 15 days after surgery
**Complete physical examination**<br>(weight, Performance status (ECOG), blood pressure))	X
**Collection of all AEs/complications** which occurred after the surgical procedures, with a special emphasis on AE of special interest:<br>- Wound healing complications,<br>- Bowel perforation or fistula,<br>- Occlusion,<br>- Bleeding,<br>- Infection or post-operative fever,	X

- Thrombo-embolic event,<br>- Cardiac failure,<br>- Respiratory failure,<br>- Multi-organ failure	

NB: this assessment and the on-adjuvant treatment period assessment could be done in the same time.

10. ON-ADJUVANT-TREATMENT PERIOD ASSESSMENTS

Adjuvant treatment will start 4 to 6 weeks after the surgery.

ASSESSMENTS / PROCEDURES DURING CHEMOTHERAPY	TIMING<br>Day -3 to day 1 of each cycle
**Complete physical examination**<br>(weight, Performance status (ECOG), blood pressure	X
**Change in concomitant treatments and symptoms**<br>Signs and symptoms, AE, concomitant review	X
**Tumor marker**<br>(serum CA-125)	X
**Laboratory tests**<br>*Haematology*: haemoglobin, white blood cells (WBC), Absolut neutrophil Count (ANC), platelet<br>*Coagulation panel*: INR or PT, aPTT,<br>*Chemistry*: creatinine, creatin clearance, ASAT, ALAT, alkaline phosphatase, total bilirubin, and urine analysis (only for patients receiving bevacizumab)<br>*Hormonology (only for patients in arm B)*: TSH, T4, T3	X

11. END OF ADJUVANT TREATMENT ASSESSMENTS

ASSESSMENTS / PROCEDURES DURING END OF ADJUVANT TREATMENT VISIT	TIMING<br>3 to 4 weeks after chemotherapy administration of last cycle
**Complete physical examination**<br>(weight, Performance status (ECOG), blood pressure	X
**Change in concomitant treatments and symptoms**<br>Signs and symptoms, AE, concomitant review	X
**Tumor marker**<br>(serum CA-125)	X
**Laboratory tests**<br>*Haematology*: haemoglobin, white blood cells (WBC), Absolut neutrophil Count (ANC), platelet<br>*Coagulation panel*: INR or PT, aPTT,<br>*Chemistry*: creatinine, creatin clearance, ASAT, ALAT, alkaline phosphatase, total bilirubin, and urine analysis (only for patients receiving bevacizumab)<br>*Hormonology (only for patients in arm B* : TSH, T4, T3.	X
**RECIST Tumour assessment**<br>*Thoracic-abdominal-pelvic CT scan; possibility to perform MRI +Chest X-Ray if CT-Scan not feasible*	X

In arm A, patient who will not progress after 6 courses of combination therapy should continue with bevacizumab (if previously introduced with chemotherapy regimen) & if the patient is eligible for continuation of therapy until 15 months in total.
In arm B, patient who will not progress after 6 courses of combination therapy should continue with pembrolizumab monotherapy or in combination with bevacizumab (if previously introduced with chemotherapy) & if the patient is

eligible for continuation of therapy until 15 months in total. There should be no interruption of pembrolizumab between the last chemotherapy cycle and maintenance period.

12. POST CHEMOTHERAPY ASSESSMENTS

12.1. Patient without treatment maintenance (ARM A without bevacizumab)

For patient receiving neither bevacizumab nor pembrolizumab, patients will be followed for the post-treatment observation period until progression, death or until lost-to-follow up for a maximum duration of 4 years.
Post-treatment observation visits will routinely be performed every 3 months during the first year of the post treatment observation period and then every 6 months during 3 years.

ASSESSMENTS / PROCEDURES DURING POST CHEMOTHERAPY PERIOD	TIMING<br>Every 3 months during the first year of the post treatment observation period and then every 6 months during 3 years
**Complete physical examination**<br>(height, weight, Performance status (ECOG), blood pressure	X
**Patient Status** (including assessment of progression, date and cause of death (if applicable), other anti-cancer-treatments (if applicable) or lost to follow-up).	X
**Tumor marker**<br>CA-125	X
**RECIST Tumour assessment**<br>*Thoracic-abdominal-pelvic CT scan; possibility to perform MRI +Chest X-Ray if CT-Scan not feasible*	X*

* tumor assessment will be performed every 6 months during the first year, then, at the time of suspected progression, imaging should be performed, and when required according to investigator.

12.2. Patient with treatment maintenance (ARM A with bevacizumab and ARM B)

12.2.1. On maintenance therapy assessment

Pembrolizumab and bevacizumab will be administered each 3 weeks during **15 months in total** (since surgery) or until disease progression which ever occur first.
There should be no interruption of pembrolizumab between the last chemotherapy cycle and maintenance period.
For patient under pembrolizumab and/or bevacizumab, visit to the investigator will be performed every 3 weeks:

ASSESSMENTS / PROCEDURES DURING MAINTENANCE THERAPY	TIMING	
	Every 3 weeks	Every 3 months
**Complete physical examination**<br>(weight, Performance status (ECOG), blood pressure))	X	
**Change in concomitant treatments and symptoms**<br>Signs and symptoms, AE, concomitant review	X	
**Tumor marker**<br>CA-125		X
**Laboratory tests**<br>*Haematology*: haemoglobin, white blood cells (WBC), Absolut neutrophil Count (ANC), platelet<br><br>*Coagulation panel*: INR or PT, aPTT,<br><br>*Chemistry*: creatinine, creatin clearance, ASAT, ALAT, alkaline phosphatase, total bilirubin, and urine analyses,	X**	X

Hormonology (only for patients in arm B): TSH, T4, T3.		
**RECIST Tumour assessment**<br>*Thoracic-abdominal-pelvic CT scan; possibility to perform MRI +Chest X-Ray if CT-Scan not feasible*		X*

* tumor assessment will be performed each 6 months, and each 3 months if required
**hematology and coagulation will be repeated if clinically indicated

12.2.2. End of maintenance period assessments

ASSESSMENTS / PROCEDURES DURING END OF MAINTENANCE PERIOD VISIT	TIMING<br>Within 4 weeks after last treatment intake.
**Complete physical examination**<br>(weight, Performance status (ECOG, blood pressure)	X
**Change in concomitant treatments and symptoms**<br>Signs and symptoms, AE, concomitant review	X
**Reason for termination of treatment**	X
**Tumor marker**<br>(serum CA-125)	X
**Laboratory tests**<br>*Haematology*: haemoglobin, white blood cells (WBC), Absolut neutrophil Count (ANC), platelet<br><br>*Coagulation panel*: INR or PT, aPTT,<br><br>*Chemistry*: creatinine, creatin clearance, ASAT, ALAT, alkaline phosphatise, total bilirubin, and urine analyse,<br>*Hormonology (only for patients in arm B)*: TSH, T4, T3.	X
**RECIST Tumour assessment**<br>*Thoracic-abdominal-pelvic CT scan; possibility to perform MRI +Chest X-Ray if CT-Scan not feasible*	X

12.2.3. Post treatment observation after maintenance therapy

ASSESSMENTS / PROCEDURES DURING POST MAINTENANCE PERIOD	TIMING<br>Every 3 months during the first year of the post treatment observation period and then every 6 months during 3 years
**Complete physical examination**<br>(weight, Performance status (ECOG, blood pressure)	X
**Patient Status** (including assessment of progression, date and cause of death (if applicable), other anti-cancer-treatments (if applicable) or lost to follow-up).	X
**Tumor marker**<br>(serum CA-125)	X
**RECIST Tumour assessment**<br>*Thoracic-abdominal-pelvic CT scan; possibility to perform MRI +Chest X-Ray if CT-Scan not feasible*	X*

* tumor assessment will be performed every 6 months during the first year, then, at the time of suspected progression, imaging should be performed, and when required according to investigator.

13. EFFICACY EVALUATION

Patients will be assessed for disease response or progression throughout the study (according to RECIST v1.1 criteria). A mandatory tumor assessment via cross sectional imaging (by CT, or MRI in case of contrast allergy) of the pelvis and abdomen and chest will be performed at baseline, within 7 days before C3, at the end of adjuvant treatment visit (3 to 4 weeks after last chemotherapy administration), then every 6 months during maintenance, and then every 6 months during the first year then, at the time of suspected progression.

Tumor measurements should be made by the same investigator/radiologist for each patient during the study to the extent that this is feasible. All subsequent follow-up imaging should be the same modality.

Results of tumor assessments must be available before next scheduled cycle (D1) in order to exclude disease progression.

Progression:
Determination of the time point of progression will be based first but not exclusively on imaging assessment of tumor manifestations according to modified RECIST v1.1 criteria (see Appendix 5). Due to the intrapelvic location of the primary tumor and the frequent occurrence of diffuse peritoneal disease at recurrence, both CT and MRI may not always be reliable for documentation of progressive disease. Therefore, criteria other than imaging may be applicable to define progressive disease such as global deterioration in health status attributable to the disease requiring a change in therapy without objective evidence of progression.

NB: In case of clinical progression, a CT scan or MRI must be performed.

14. SAFETY EVALUATION / ADVERSE EVENTS

14.1. Definitions

14.1.1. Adverse Event

An adverse event (AE) is the development of an undesirable medical condition or the deterioration of a pre-existing medical condition following or during exposure to a pharmaceutical product, whether or not considered causally related to the product. An undesirable medical condition can be symptoms (e.g. nausea, chest pain), signs (e.g. tachycardia, enlarged liver) or the abnormal results of an investigation (e.g. laboratory findings, electrocardiogram).

All events occurring during the trial (from the inform consent signature to 30 days after treatment discontinuation) will be listed on the eCRF.

As far as possible, each AE should be evaluated to determine:

1. The severity of the grade (grade 1 to 5 according to CTCAE V4.03 criteria)
2. The relationship with each trial product (suspected/no suspected)

The investigator must do his best to explain each adverse event and establish, when there is, and the link with each trial product.

The link cause and effect will be established for each trial product in the following manner:

- No, there is no reasonable causal relationship between the investigational drug administered and the AE.
- Yes, there is a reasonable causal relationship between the investigational drug administered and the AE according to the following criteria:
    - The product pharmacology is known

- The effects are similar in nature that known effect still been reported for the product or for another product of the same family or the same category, adverse event previously reported in the literature for a similar product and considered to be drug-related
- Adverse event closely based on the treatment period (between start of administration – administration period – end of administration – treatment discontinuation period) or positive rechallenge of the product.

3. degree of severity or seriousness

14.1.2. Serious Adverse Event

A serious adverse event (SAE) is defined as any AE that fulfils one or more of the following criteria:

- Results in death,
- Is immediately life-threatening,
- Requires in-patient hospitalization or prolongation of existing patient hospitalization,
- Results in persistent or significant disability / incapacity or substantial disruption of the ability to conduct normal life functions,
- Is a congenital anomaly / birth defect,
- Is another medically significant

The expression “life threatening” is reserved to immediate vital threat, at the time of the adverse event, and regardless of consequences of a symptomatic treatment.

The term of “disability” and “incapacity” corresponds to all physical or mental disability, either temporary or permanent, clinically relevant and with physical and/or patient’s quality of life consequences.

Is considered to be “medically significant” any clinical event or laboratory result judged as serious by the investigator or/and the sponsor and not corresponding to intensity criteria defined above. Patient cannot be put at risk by it and require medical intervention to prevent from issue corresponding to one of the intensity criteria defined above (overdose, second primary cancers, intensive treatment in an emergency room can be considered as medically significant).

The following events, in the context of this trial, should not be considered as SAEs. No SAE form is required and they are exempt from expedited reporting. They must be reported on the appropriate CRF section:

- An hospitalization < 24 hours without any other seriousness criteria
- An hospitalization planned for a concerned care, medical acts, all exam or investigation done on an outpatient basis and without any associated seriousness criteria
- An hospitalization planned at the beginning of the trial and/or provided in the protocol (biopsy, chemotherapy…) and/or for progression disease management
- An hospitalization or prolongation of hospitalization caused by a drug related event related to product administration or planned transfusion
- An event related to the disease progression
- An event occurring between inform consent signature and treatment administration, and not related to protocol procedures
- A death related to disease progression

14.1.3. Expected Serious Adverse Event

An expected serious adverse event is an event that is mentioned in the Investigator Brochure or Summary of Product Characteristics latest version for product that granted marketing authorization, even if it is not in the same studied population.

14.1.4. Unexpected Serious Adverse Event or SUSAR

An unexpected adverse event is an event that is not mentioned or with a different nature or intensity that in the Investigator Brochure or Summary of Product Characteristics latest version for product that granted marketing authorization, even if it is not in the same studied population.

14.1.5. New fact

A new fact is any new data which may lead to:

- A reassessment of the risk-benefits balance of the research or product used on the research,
- Changes in the use of the products, the conduct or documents related to the research,
- Suspend or interrupt or modify the protocol of the search or similar searches.

14.1.6. Events of Clinical Interest (ECI)

An event of clinical interest (ECI) is one of scientific and medical interest specific to understanding of the Investigational Product and may require close monitoring and rapid communication by the investigator to the sponsor. An ECI may be serious or non-serious. The rapid reporting of ECIs allows ongoing surveillance of these events in order to characterize and understand them in association with the use of this investigational product.

Selected non-serious and serious adverse events (SAE) are also known as ECI and must be recorded as such on the SAE report forms/worksheets and reported within 24 hours to the Sponsor.

Events of clinical interest for this trial include:

Table 1: Events of Clinical Interest

**Pneumonitis (reported as ECI if ≥ Grade 2)**		
Acute interstitial pneumonitis	Interstitial lung disease	Pneumonitis
**Colitis (reported as ECI if ≥ Grade 2 or any grade resulting in dose modification or use of systemic steroids to treat the AE)**		
Intestinal Obstruction	Colitis	Colitis microscopic
Enterocolitis	Enterocolitis hemorrhagic	Gastrointestinal perforation
Necrotizing colitis	Diarrhea	
**Endocrine (reported as ECI if ≥ Grade 3 or ≥ Grade 2 and resulting in dose modification or use of systemic steroids to treat the AE)**		
Adrenal Insufficiency	Hyperthyroidism	Hypophysitis
Hypopituitarism	Hypothyroidism	Thyroid disorder
Thyroiditis	Hyperglycemia, if ≥Grade 3 and associated with ketosis or metabolic acidosis (DKA)	
**Endocrine (reported as ECI)**		
Type 1 diabetes mellitus (if new onset)		
**Hematologic (reported as ECI if ≥ Grade 3 or any grade resulting in dose modification or use of systemic steroids to treat the AE)**		
Autoimmune hemolytic anemia	Aplastic anemia	Thrombotic Thrombocytopenic Purpura (TTP)
Idiopathic (or immune) Thrombocytopenia Purpura (ITP)	Disseminated Intravascular Coagulation (DIC)	Haemolytic Uraemic Syndrome (HUS)
Any Grade 4 anemia regardless of underlying mechanism		
**Hepatic (reported as ECI if ≥ Grade 2,** or any grade resulting in dose modification or use of systemic steroids to treat the AE)		
Hepatitis	Autoimmune hepatitis	Transaminase elevations (ALT and/or AST)
**Infusion Reactions (reported as ECI for any grade)**		
Allergic reaction	Anaphylaxis	Cytokine release syndrome
Serum sickness	Infusion reactions	Infusion-like reactions
**Neurologic (reported as ECI for any grade)**		
Autoimmune neuropathy	Guillain-Barre syndrome	Demyelinating polyneuropathy
Myasthenic syndrome		
**Ocular** (**report as ECI if ≥ Grade 2 or any grade resulting in dose modification or use of systemic steroids to treat the AE)**		
Uveitis	Iritis	
**Renal (reported as ECI if ≥ Grade 2)**		
Nephritis	Nephritis autoimmune	Renal Failure
Renal failure acute	Creatinine elevations (report as ECI if ≥Grade 3 or any grade resulting in dose modification or use of systemic steroids to treat the AE)	
**Skin (reported as ECI for any grade)**		
Dermatitis exfoliative	Erythema multiforme	Stevens-Johnson syndrome
Toxic epidermal necrolysis		
**Skin (reported as ECI if ≥ Grade 3)**		
Pruritus	Rash	Rash generalized
Rash maculo-papular		
Any rash considered clinically significant in the physician's judgment		
**Other (reported as ECI for any grade)**		
Myocarditis	Pancreatitis	Pericarditis
Any other Grade 3 event which is considered immune-related by the physician		

14.2. Assessment of intensity

The investigator evaluates the intensity of the events according to CTCAE v4.03 criteria in the eCRF.
The CTCAE reports 1 to 5 grades with specific severity clinical description of each adverse event following the general procedure:

CTC Grade	Equivalent To	Definition
Grade 1	Mild	asymptomatic or mild symptoms; clinical or diagnostic observations only; intervention not indicated
Grade 2	Moderate	minimal, local or noninvasive intervention indicated; limiting age-appropriate instrumental activity of daily living (ADL)
Grade 3	Severe	severe or medical significant but not immediately life-threatening hospitalization or prolongation of hospitalization indicated; disabling; limiting self-care ADL
Grade 4	Life threatening	Life-threatening consequences; urgent intervention indicated
Grade 5	Death	death related to AE

Causality assessment:
The investigator evaluates the SAE causal effect with experimental product, acts/procedures added by the research according to the following degrees of causality:

Imputability Grade	Evaluation criteria
Certain	• Clinical event or laboratory abnormalities, with plausible temporal relationship with drug administration<br>• Event that could not be explained by no illness or other drug<br>• Plausible answer to the treatment discontinuation<br>• Event identified by pharmacologic and physio pathologic point of view<br>• Positive re-challenge (where required)
Likely	• Clinical event or laboratory abnormalities, with reasonable temporal relationship with drug administration<br>• Not appear to be related to any illness or other drug<br>• Reasonable answer to the treatment discontinuation (clinical)<br>• Re-challenge information required
Possible	• Clinical event or laboratory abnormalities, with reasonable temporal relationship with drug administration<br>• Not appear to be related to any illness or other drug<br>• Not clear or lacking information concerning treatment discontinuation
Unlikely	• Clinical event or laboratory abnormalities, with improbable temporal relationship with drug administration<br>• Illness or other drug explaining plausible occurrence of the event

14.3. Reporting of Serious Adverse Event

14.3.1. Initial notification

The investigator informs ARCAGY-GINECO pharmacovigilance of all SAEs, expected or unexpected, imputable to the research or not, occurring during the trial (after consent signature) and until the 30th day after treatment discontinuation (last treatment administration).

All delayed SAEs (occurring after this 30 day period) considered as having a reasonable relationship with experimental product, must be reported immediately.

The notification shall be made using an SAE form, documented as accurately as possible, by fax, or email to the ARCAGY-GINECO pharmacovigilance. The form has to be dated, signed by the investigator and sent immediately when he or she becomes aware of the event.

[redacted]

The investigator will indicate for ea[redacted]

- Identity patient number
- Identify of the investigator, site number,
- Event description as clearly as possible using medical terminology,
- Intensity,
- Seriousness criteria,
- Onset and end date,
- Current or previous medical history of the patient,
- Treatments received (experimental and concomitant treatments,
- Measures taken and need of corrective treatment,
- If the trial drug has been discontinued,
- Evolution of the event. In case of no fatal incidence, the evolution will be monitored up until the condition is cured, or return to previous state or potential return to stability,
- Assessment of the relationship of the event with the study drug or research related constraint (treatment discontinuation period, additional examinations requested in the conduct of the research, etc),
- Assessment of the relationship of the event with the study drugs, pathology treated or other treatment.

If possible, the investigator should join to the SAE form:

- A copy of the hospitalization or prolongation of hospitalization report,
- A copy of all additional exams realised, including relevant results containing laboratory normal values,
- All judged relevant documents,
- A copy of the final autopsy report.

All these documents must be anonymised.
Additional information's could be requested (by fax, phone or during monitoring visit) by the pharmacovigilance or an ARCAGY-GINECO CRA.

In case of unexpected SAE, additional information will be requested by the pharmacovigilance as soon as possible.

14.3.2. SAE follow up

The investigator will be assuming the appropriate medical follow-up until resolution or stabilisation of the effect or until the patient death. This could mean a prolonged follow-up after the patient study withdrawal.
The investigator has to answer to additional information requested by the ARCAGY-GINECO pharmacovigilance to document the initial notification.

The investigator forwards the additional information to the pharmacovigilance with an SAE form (checking the box "follow up N°X" to inform it is a follow up and not an initial notification) within 48 hours after they were obtained. He also forwards a last follow up at the SAE resolution or stabilisation.
The investigator keeps the SAE documentation, in order to give more information on those previously forwarded.

14.4. Pregnancy notification

All confirmed or suspected pregnancy (positive test) during the study and until 30 days after treatment discontinuation must be notified immediately to the pharmacovigilance using a pregnancy declaration form.
The investigational product should be discontinued immediately. The patient should be monitored by a doctor experienced in teratology to monitor and give appropriate advices in this sort of pregnancy.

The investigator should provide follow-up information regarding the course of the pregnancy, including perinatal and neonatal outcome using a pregnancy declaration form.

The outcome of pregnancy could be:

- "Normal" viable birth
- Spontaneous or therapeutic abortion, infant death, neonatal death or/and congenital anomaly

In all case, it is a SAE that should be notified within 24 hours flowing discovery.

Exposure during breastfeeding

The exposure during breastfeeding happens when a child could have been exposed to a drug via breastfeeding of a treated by a study drug mother.
It is not considered as a SAE, but it should be notified by the investigator to the sponsor from apprehension. After a suspected pregnancy not confirmed, the investigator will decide of the patient's trial participation.

14.5. Overdose/misuse notification

Reports of overdose of study drug with or without symptoms should be handled as SAE.
Overdose means that the patient was administered a higher dose than the dose prescribed for the assigned treatment group in this protocol.
Any event associated with (or observed in association with) an overdose (accidental or deliberate) or abuse of product and / or a cessation phenomenon is considered as serious adverse event.

Although an overdose is no longer considered as an adverse event by the authorities (unless it involves an event with serious consequences), information about overdoses among participants in a clinical trial will be collected.

For purposes of this trial, an overdose of pembrolizumab will be defined as any dose of 1,000 mg or greater (≥5 times the indicated dose). No specific information is available on the treatment of overdose of pembrolizumab. Appropriate supportive treatment should be provided if clinically indicated. In the event of overdose, the subject should be observed closely for signs of toxicity. Appropriate supportive treatment should be provided if clinically indicated.
If an AE is associated with ("results from") the overdose of a Pembrolizumab, the AE is reported as a SAE, even if no other seriousness criteria are met.
If a dose of Pembrolizumab meeting the protocol definition of overdose is taken without any associated clinical symptoms or abnormal laboratory results, the overdose is reported as a non-serious Event of Clinical Interest (ECI), using the terminology "accidental or intentional overdose without adverse effect."

If a patient has an overdose (as defined above) during the course of the study, the principal investigator or his representative must contact ARCAGY-GINECO within 5 days after discovery after the principal investigator or his representative was aware of overdose (if a SAE occurs in association with overdose should meet the deadline for

reporting SAE). Tracking information on the evolution of overdose should also be reported to the pharmacovigilance of ARCAGY-GINECO.

14.6. Death notification

If an AE conducts to the death of a patient, it will be considered as an SAE and will be notified to the pharmacovigilance using a SAE form.

All disease progression related death is not to notify as SAE and have to be listed on the eCRF with the causality.

15. STATISTICAL CONSIDERATIONS

15.1. Determination of sample size

The sample size calculation in the experimental arm is based on the Fleming's A'Hern single-stage design in which number of patients is estimated for success rate respectively larger than P0 and smaller than P1, with reasonable values of power and risk.

In our study, we consider that a rate of complete resection rate < 50% (P0) is insufficient for the combination of Pembrolizumab and chemotherapy in the neo adjuvant setting, while a rate of complete resection rate ≥ 70% (P1) should be considered as sufficient to justify further evaluation of the combination of Pembrolizumab plus chemotherapy.
Using a one-sided risk α=0.05 and a power of 90%, 54 evaluable patients are required in the Pembrolizumab plus chemotherapy group.

No formal comparison will be made between the two arms. The control arm is only designed to validate the complete resection rate in a 'standard' situation. Therefore, more patients will be included in the experimental arm justifying the choice of a 1: 2 randomization rate. The control group requires 27 patients. At the end of the study, experimental treatment will be considering as promising if at least 33 patients have a complete resection (CC0) over the 54 evaluable patients expected in the experimental arm.

Assuming that 10% of patients will be lost to follow-up or non-evaluable for the primary criteria, a total of 90 patients will be included and randomized (60 in the experimental arm and 30 in the control arm).

An **Independent Data Monitoring Committee** will be in charge of reviewing safety data at regular intervals throughout the study.

Stratification

- Centre
- The FIGO-stage: IIIC versus IV
- Metastases volume (< 5cm; ≥ 5cm)
- Planned used of Bevacizumab after IDS (either arm A or B) (yes or no)

Randomization will be performed using a minimization process.

15.2. General considerations

Study populations

Intention-to-treat (ITT) population: is defined as all patients randomized in the trial, regardless of whether they actually received treatment. The population will be described according to randomization.

Modified Intention-to-treat population: is a subgroup of the ITT population containing all patients without any major protocol violation which may bias efficacy evaluation and which received at least one dose of pembrolizumab. Major deviations will be defined during the blind review.

Safety population: All patients having received at least one administration of pembrolizumab.

This analysis will include following parameters:

Concomitant medications

The concomitant medications will be presented by International Non-proprietary Name (INN) and Low-Level term (LLT) according to the WHO-DRUG coding system.

Vital signs

Descriptive statistics will be performed for the following parameters by study group and globally:

- Height & Weight
- ECOG performance status
- Blood pressure
- ECG

Laboratory parameters

Descriptive summaries will be performed for the following parameters by study group and globally:

- Urinalysis
- Biochemistry: creatinine, ASAT, ALAT, Alkaline Phosphatase, total bilirubin, Thyroid Stimulating Hormone (TSH)
- Haematology: haemoglobin, white blood cell count, neutrophils and platelets
- Haemostasis: International Normalized Ratio (INR) or Prothrombin Time (PT) and Activated Partial Thromboplastin Time (aPTT)

Description of the study parameters

Parameters concerning study conduct and exposure to the study products will be described by group and globally:

- Study product exposure
- Study product compliance

15.3. Stopping rules

Both the sponsor and the coordinating investigator reserve the right to terminate the study at any time. Should this be necessary, both parties will arrange the procedures on an individual study basis after review and consultation. In terminating the study, the sponsor and the coordinating investigator will ensure that adequate consideration is given to the protection of the patient's interests.

The ethic committee and the competent authority need to be notified about the end of the trial or early termination of the trial.

15.4. Statistical analysis

No statistical test will be performed in this study.
Quantitative variables will be described using means and standard deviations for continuous variables (+median min/max).
Qualitative variables will be described using frequencies and percentages.
All parameters will be presented with their confidence interval (CI). All confidence intervals will be presented two-sided with a confidence level of 95%, except for primary endpoint for which sample size calculation is associated with a one-sided 95% CI.

Survival parameters (PFIbio, PFS, OS) will be analyzed using the Kaplan-Meier method and will be described in terms of median per arm, associated with its 2-sided 95% CI.

15.5. Interim analysis

Safety analyses will be performed during the study. These analyses are planned:

- After the randomization of 10 patients treated with cycles with the combination of bevacizumab plus pembrolizumab, (after 4 cycles)
- After the randomization of 20 patients in the experimental arm once these patients have received 4 neo adjuvant treatment cycles.
- Sequentially for the subgroup of patients having completed 2 consecutive treatment cycles with the combination of bevacizumab plus pembrolizumab

Safety analysis data will be assessed by the Data Safety Monitoring Board (DSMB). The DSMB members will be informed prospectively of all serious and unexpected adverse events.

Safety analysis of the combination bevacizumab plus pembrolizumab will be focused on Dose Limiting Toxicity (DLT) events of special interest defined as all non-hematologic events of grade 3/4 according to NCI CTCAE v4.03 occurring during the first two cycles of treatment,
Including:

- Conditions which may be suggestive of an immune-related disorder
- Cases of an elevated ALT or AST in combination with either an elevated bilirubin or clinical jaundice
- Events suggestive of hypersensitivity, cytokine release, influenza-like illness, systemic inflammatory response syndrome (SIRS) or infusion reaction syndromes
- And excluding:
- Nausea, vomiting, fatigue, diarrhea, edema, hyperglycemia and changes in serum electrolytes.

Monitoring of the safety of the combination will be based on the occurrence of DLT compared to a rate deemed acceptable based on our clinical experience (absolute excess toxicity). This comparison will be done by specifically considering subgroup of patients receiving the combination bevacizumab plus pembrolizumab, and in regards of patients receiving bevacizumab in the control arm. A descriptive analysis of all grade 3/4 toxicities in these subgroups of patients receiving bevacizumab with or without pembrolizumab will be performed.

The safety of the combination pembro+beva+chimio will be performed according to Kramar and Mollevi rules: 'Early Stopping Rules in Clinical Trials Based on Sequential Monitoring of Serious Adverse Events' (Medical Decision Making # 2009, 29(3): 343-350).
After the occurrence of each DLT, the stopping rule will compare the number of included patients receiving the combination Pembrolizumab + bevacizumab to the number of patients satisfying maximum DLT criteria. The nominal type I error, power, and average sample number (ASN) under specific hypotheses are obtained through simulations.
Using 5% as the maximal acceptable rate of DLT with the combination, we can define the following rules:

Analysis after the occurrence of kth event	Number of patients that should be included and received combination at the time of the kth analysis
2	7
3	15
4	25
5	33
6	42

No statistical rules are invoked for the first observed SAE, that is, k =1.

For example, using this procedure, if we observe the second DLT event among the first N=12 patients or the third among the first N=24, we can conclude that the toxic rate percentage is significantly greater than 3%, and we would recommend early trial termination due to an unacceptable toxic rate.

The DSMB members will be informed prospectively of all serious and unexpected adverse events, and may if necessary (unexpected levels of toxic events of grade 4 in the experimental arm with Bevacizumab relative to the control arm with Bevacizumab) at any time propose a schema changes treatment in the experimental arm.

15.6. Endpoint analysis

15.6.1. Primary endpoint

Rate of complete debulking

Rate of complete debulking is based on the presence or absence of residual ovarian cancer following debulking. This rate is recognized as one of the most important prognostic factors.
After resection, cyto-reduction has to be assessed according to a score of radicalism (CCI: Completeness Cytoreductive Index) described by P. Sugarbaker as followed:

Resection is classified as complete or incomplete:

- CC0: no macroscopic residual disease,
- CC1: residual disease < 0.25 cm,
- CC2: residual disease 0.25 cm <R< 2.5 cm,
- CC3: residual disease >2.5 cm.

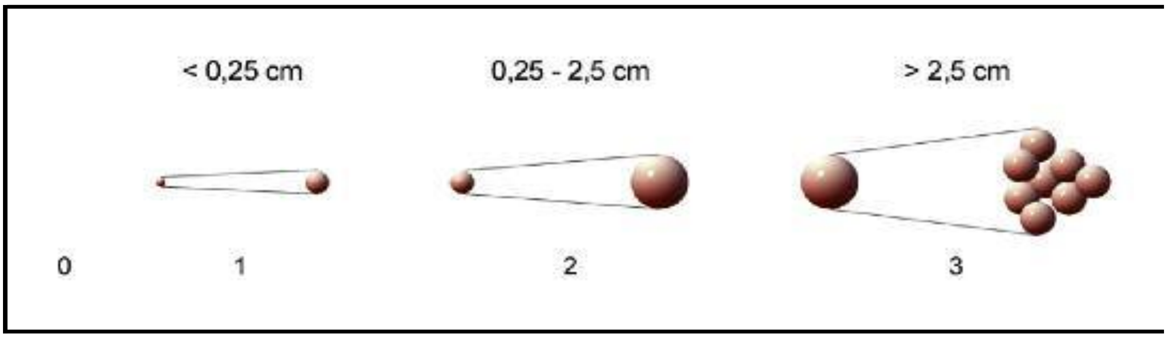

The number of complete debulking score CC0 (success), evaluated by central medical review, will be presented in both arms. A 95% one- sided Confident Interval (95CI) will be associated to the rate of success.
Primary endpoint will be analyzed on the modified ITT population. If surgery could not be performed due to progression disease before the end of the neo adjuvant period, or due to insufficient efficacy of the neo adjuvant therapy, patient will be considered as failure. If surgery could not be performed due to other reason, patients will be considered as non-evaluable. Thirty-three successes over the 54 evaluable patients are expected to declare the experimental treatment as promising. If the number of evaluable patients is different than 54, the lower limit of the one sided 95%CI will be used and compared to 50% to conclude.

A sensitivity analysis will be performed to measure the complete resection rate using the CCI score evaluated by locally surgeons.

15.6.2. Secondary endpoints

All the planned analyses for the secondary efficacy criteria will be detailed in the statistical analysis plan. Those analyses will be performed on modified-ITT population.

CCI score

Rate of patient in CC0 to CC3 will be presented according to central review and by local center

PCI score

Sugarbaker index is also known as Peritoneal Cancer Index. The centers and the central medical review measure the distribution of cancer in 13 different regions of the abdomen and gives each region a score from 1 to 3. These scores are added together in an overall score. The score allows an estimate of prognosis: the lower the score is, the better is the prognosis.
PCI score will be presented according to central review and by center.
CCI score and PCI scores will be presented by arm with their 95% CI.

Overall Response rate (ORR) Response rate to neo adjuvant chemotherapy +/- pembrolizumab assessed by RECIST 1.1 after the first 4 cycles compared to baseline.

ORR is defined as the rate of patients with an observed tumor response (CR+PR).
ORR will be presented by arm with their 95% CI.

Best response
Best response to the global strategy of neoadjuvant chemotherapy +/- Pembrolizumab assessed by RECIST 1.1, CA125 and physical examination at the end of treatment visit compared to baseline
Best overall response will be assessed by tumor measurements and is defined as best response observed at any time from the date of randomization until the end of treatment. Best response will be presented by arm with their 95% CI
The response rate will be evaluated by subgroup based to PD-L1 expression (yes or not) on tumor samples.
Correlation between response rate and PDL1 expression will be performed in the experimental arm and test using a fisher exact test.

The rate of pCR after surgery in both arm
Pathological reports must specify the amount and localization (Peritoneum, organ, node) of any tumor residual.
Pathological complete response is defined a No tumor residue found in the tissue collected during the surgery (62).
This rate will be evaluated on all patient included in the trial.

Progression Free Interval (PFS)
PFS will be assessed by tumor measurements according to the Response Evaluation Criteria in Solid Tumors version 1.1 and will be measured from the date of randomization until the date of disease progression or death, from any cause, whichever occurs first.
Progressive disease (PD) will be diagnosed in this trial based on tumor assessment shows PD according to RECIST 1.1 criteria. Progressive disease shall not be diagnosed in case of “CA-125” progression alone.

Median PFS as well as PFS rate at 6, 12, 18 and 24 months post randomization in both arms will be presented based on a Kaplan-Meier approach on ITT population. A 95% Confident Interval (CI95) will be estimated at each time point. Patients who have not progressed or died at the time of analysis will be censored at the time of the latest date of assessment from their last evaluable RECIST assessment. A sensitivity analysis will be performed on PP population in order to assess the robustness of the results.

Biologic Progression Free Interval (PFIbio)
PFIbio is defined on the basis of a progressive serial elevation of serum CA-125 from the date of randomization to biological progression using GCIG criteria based on CA-125.

PFI_{bio} will be analyzed based on serum CA-125 values assessed according to the GCIG criteria. Time from the date of randomization to biological progression using GCIG criteria based on CA-125 will be calculated. Patients without Biological progression will be censored to the last CA-125 evaluation. Median PFI_{bio} in both arms will be presented based on a Kaplan-Meier approach and will be associated with its 95% Confident Interval (CI95)

Overall survival
Overall survival is defined as the time from the date of randomization to the date of death, regardless the cause of the death.

Median OS in both arms will be presented based on a Kaplan-Meier approach and will be associated with its 95% Confident Interval (CI95). Any patient not known to have died at the time of analysis will be censored based on the last recorded date on which the patient was known to be alive.
Cause of death will be described.

Safety endpoints
Safety parameter will be performed according to NCI CTCAE v4.03.

The analysis of adverse events will be performed to evaluate the number of patients with at least:
- One adverse event and the number of adverse events by arm,
- One Serious AE,
- One AE with grade 3 or above.

Particular attention will be done to post-operative mortality. Rate of patients who died from any cause during the 30 days following surgery will be presented with its 95%CI.

Serious adverse events will be recorded together with causal relationship to the study product.
All adverse events will be summarized by patients. The analysis of all adverse events (emergent or not emergent) will be performed to evaluate the number of patients with at least one adverse event and the number of adverse events by study group.
Descriptive statistics will be provided for characterizing and assessing patient tolerance to treatment. Adverse events of special interest specific for pembrolizumab should be analyzed in addition to safety analysis based on system organ class classifications. The period of safety analysis will be defined from the first dose of any study treatment to 30 days after end of last study treatment.

A descriptive analysis based on the Clavien-Dindo classification/grading will be performed on:
- Operative and post-operative complications rate
- Operative and post-operative complications (described below) occurring during surgery and within the 30 days after surgery:
    - Wound healing complications,
    - Bowel perforation or fistula,
    - Occlusion,
    - Bleeding,
    - Infection or post-operative fever,
    - Thrombo-embolic event,
    - Cardiac failure,
    - Respiratory failure,
    - Multi-organ failure
- Calculation of the CCi score

Post-operative mortality
Post-operative mortality defined as rate of patients died due to any cause during the 30 days post-surgery.

Post-operative morbidity (CCi).
Post-operative morbidity is defined as a comprehensive complication index (CCi) that integrates all events with their respective severity during the 30 days post-surgery. The CCi summarizes all postoperative complications and is more sensitive than preexisting morbidity endpoints. It may serve as a standardized and widely applicable primary endpoint in surgical trials and other interventional fields of medicine. The CCi can be readily computed on the basis of tabulated complications according to the Clavien-Dindo classification (available at www.assessurgery.com).

15.6.3. Exploratory analysis

The CCI and the PCI score were evaluated by the center and by a central medical review. The differences between the two assessments will be described and discussed.

16. TRANSLATIONAL RESEARCH PROGRAM

This translational research program will be realized according to the funding received:

Objectives:

Although the clinical responses are impressive, in melanoma and other tumors around 60% of patients present a primary resistance to anti-PD1 treatments. This resistance is not well understood. Indeed, although early reports have shown that patients with PDL1-expressing tumor have a higher response rate[12], not all $PDL1^{+}$ tumors will respond and a significant proportion of $PDL1^{neg}$ tumors will respond[20]. Type-II[21] and type-I IFN[22] upregulate PDL1 expression that might confer resistance to anti-PD1 treatment, but also explaining response of tumors $PDL1^{neg}$ before therapy.
While anti-CTLA4 therapy was recently reported to broaden the melanoma-reactive $CD8^{+}$ T cells response[23], rather than boosting the pre-existing immune response, PD1 inhibition will reverse exhausted memory $CD8^{+}$ T cells[24]. Very recent reports demonstrate that a pre-existing $CD8^{+}$ T cell response correlates with therapeutic efficacy of anti-PD1 in melanoma[25] and that the number of non-synonymous mutations determines sensitivity to PD1 blockade in non-small cell lung cancer[26].
The biological studies of the present protocol are designed to identify different immune and/or tumor parameters between responders and non-responders to anti-PD1 treatment in order to establish a strategy to increase the response rate. The hypotheses sustaining this objective are: 1) responding patients display i) pre-existing anti-tumor immune response, ii) PDL1/L2 ligands expression in the tumor or its microenvironment initially or induced upon therapy, iii) high mutation load in their tumor; 2) anti-PD1 treatment elicits cellular long lasting anti-tumor immunity against multiple TAA, but also regulates TFh and humoral anti-tumor immunity; 3) non-responding patients are dominated by other immunosuppressive networks.
The primary objective will be to define biomarkers (pre-existing immune parameters and/or tumor characteristics) predicting the response to anti-PD1 treatment or to alternative best options (other ICP) and combinations to improve treatment efficacy. This is of prime importance because: i) probably not all patients with ovarian cancer patients are expected to benefit from the therapy, ii) this treatment is potentially associated to significant toxicities (auto-immune manifestations, less than < 5% grade 3-4), and iii) this treatment is expensive. Importantly, for the patients that do not benefit from the anti-PD1 treatment, biomarkers allowing defining alternative best option need to be determined.
The response to anti-PD1 in combination with chemotherapy will be the primary endpoint associated with the primary objective. The clinical response will be determined as complete resection rate (CRR) and/or as pathological complete response (pCR). We will analyse the following immune parameters according to response to anti-PD1 treatment:

1) Pre-existing anti-tumor immunity (immune infiltrate composition/ structuration, peripheral immune parameters, anti-TAA $CD8^{+}$ T cell and B cell intensity/ diversity),
2) PDL1/L2 expression/regulation,
3) Tumor molecular alteration and mutation load (CGH, RNAseq),
4) Other immunosuppressive pathways (immune checkpoints, IHC, RNAseq).

Importantly, in all current clinical trials, none ancillary study has evaluated the consequence of the anti-PD1 blockade on the humoral response. Indeed, in human, PD1 is highly expressed on Follicular Helper T cells (TFh)[43]. Interaction of PD1 on TFh with PDL1/PDL2 on B cells finely tunes the humoral immune response[44]. Indeed, ectopic tertiary lymphoid structures (TLS), with segregated T and B cell zones, have been documented in many cancers, including melanoma[45-48] suggesting the local induction or reactivation of an Ab response. **Thus as, the secondary objectives** will follow 2 axes:

- First, we will decipher the **importance of the PD1 pathway in regulating the intensity of the humoral response and its contribution in therapeutic response. The associated endpoint will be the increase of the anti-tumor humoral response under anti-PD1 treatment**. This will be evaluated, before and during therapy, through the analysis of 1) TLS, B cell populations, and IgG (inflammatory)/ IgA (anti-inflammatory)-producing plasma cells in tumors (IHC), 2) anti-tumor specific humoral response at tumor site and in blood, 3) TFh frequency, activation status and function, and iv) BCR Repertoire (RNAseq).

- The other secondary objective will aim at defining **biomarkers for treatment suspension in responding patients**. This will represent the next major medical concern after the definition of biomarkers of therapeutic response. The associated endpoint will be the **stabilisation of key anti-tumor immune parameters during anti-PD1 treatment in responding patients**. For this purpose the parameters evaluated in the primary objective at inclusion will be monitored during the course of the therapy (*see* primary endpoint).

Technics and Methods:

1 Mandatory biological specimens: FFPE tumor biopsies at inclusion and at surgery following neo-adjuvant treatment and at relapse (optional)

1.1 Molecular profiling, muitational landscape and transcriptome

- CGH array profile and NGS using the ProfilER panel (EudraCT : 2013-003058-25)
- Exome and RNA sequencing from whole FFPE tumor
  - From RNAseq: System biology approaches from transcriptome analysis for immune signature and pathway analysis to discover immune resistance mechanisms and targets.
  - From Exomeseq (tumor+ blood DNA see 3) + RNAseq: Neo-antigen identification (mutation, frame shift, splicing deregulation, genetic instability (fusion, amplification,…) coupled to MHC class I and II peptide binding prediction (see 4 & 5)

- IHC for PDL1 and PDL2 expression on tumor cells and immune infiltrate
- IHC for other immune check point receptors and ligands
- Immune infiltrate by IHC for T cells (CD3, CD8), B cells (CD20, IgG), NK (NKp46), Treg (FOXP3).
- Immune cell activation: double IHC and multiple-IF staining for CD3 or CD8 with KI67, ICOS, CD39, CD137, PD1, CTLA4
- IHC and multiple-IF for DC subpopulations (DC-LAMP, CD1a, BDCA3/Clec9A, pDC/BDCA2), macrophages (CD163, CD68, M-CSFR) & neutrophils (MPO), other myeloid cells (CD11b/c).

1.2 Immune infiltrate analysis:

- IHC for PDL1 and PDL2 expression on tumor cells and immune infiltrate
- IHC for other immune check point receptors and ligands
- Immune infiltrate by IHC for T cells (CD3, CD8), B cells (CD20, IgG), NK (NKp46), Treg (FOXP3).
- Immune cell activation: double IHC and multiple-IF staining for CD3 or CD8 with KI67, ICOS, CD39, CD137, PD1, CTLA4
- IHC and multiple-IF for DC subpopulations (DC-LAMP, CD1a, BDCA3/Clec9A, pDC/BDCA2), macrophages (CD163, CD68, M-CSFR) & neutrophils (MPO), other myeloid cells (CD11b/c).

2 Mandatory plasma/serum before treatment (W0, W3, W12, relapse)

- Systemic humoral anti-tumor immunity.
- Levels of circulating growth factors and cytokines (>20 cytokines by Luminex)
- Metabolome with a high field NMR untargeted approach
- Soluble PDL1 and PDL2 by Elisa.
- Host SNP impact on therapeutic response.

3 Mandatory frozen whole blood

- Exome sequencing needed to determine mutations and neo-epitopes (see 1.1)
- Host SNP impact on therapeutic response.

4 Mandatory frozen PBMC before and on treatment (W0, W3, W12, relapse)

- Frozen viable PBMC for phenotype and T cells functional assay.
- Frequency of circulating TAA-specific CD4 & CD8+ T cells (link to 1.1)
- Measure TCR repertoire diversity through multiplexing PCR approach

6 Optional fresh biological tumor specimen following neo-adjuvant therapy when available:

- Frozen viable cells from tumor suspension
- 12 color multiparametric flow cytometry and T cells functional assay.
- Frequency of tumor infiltrating TAA-specific CD4 & CD8+ T cells (link to 1.1).

Supporting Tumor Tissue Collection and Correlative Studies Blood Sampling:

- **Tumor tissue:**
  - Mandatory FFPE tumor biopsies at inclusion (1bloc)
  - Mandatory FFPE surgical tumor specimen following neo-adjuvant treatment (4 blocs)
  - Optional Fresh biological tumor specimen following neo-adjuvant therapy (Frozen viable cells from tumor suspension)
  - Optional FFPE tumor biopsies at relapse (1bloc)

- **Whole blood** W0 only: Mandatory 1 tube 4 ml for constitutive DNA (2 frozen vials)

- **Frozen PBMC W0, W3, W12, relapse**:
  - Mandatory 8 tubes of ficol (= 40ml), 10 vials of viable cryopreserved PBMC (10x106PBMC/vials, 1ml volume in serum+DMSO)

- **Plasma/or serum W0, W3, W12, relapse:**

- Mandatory plasma from the 8 ficol tubes (see above): 10 vials of 500microL plasma
- Optional 1 tube 5 ml without preservative: 5 tubes of 500microL serum

- **Ascitis:** Optional when available
  Cryotubes: (After centrifugation, and washing in cryotube in 10% DMSO.
  50 million cells per ml/tube / 10 tubes at each time

17. STUDY COMMITTEES

17.1.1. Steering committee

A steering committee will be composed of Sponsor representatives, including the project manager, the principal investigator, three representatives of main investigators involved in the study, at least one surgeon, and other external participants, as needed.

It will be regularly informed of the accrual rate of inclusion and of any emergent problems and will review the efficacy and safety data at the end of the study.

Meetings via teleconference will be scheduled when necessary.

On an ongoing basis, the person responsible for pharmacovigilance, the principal investigator and the project manager will review all Grade ≥ 3 adverse events and Critical Event.

17.1.2. Data Safety Monitoring Committee (DSMB)

The Data Safety Monitoring Committee (DSMB) will be constituted by the sponsor on the steering committee proposal, and will be composed of statisticians and two medical experts (one surgeon) in the field of ovarian cancer.

In general, the role of the DSMB will be to ensure the safety of the patient and the ethical conduct of the study.

In particular this committee will have to:

- Meet after the randomization of 10 patients treated with cycles with the combination of bevacizumab plus pembrolizumab (after 4 cycles)
- Meet after the 20th included patient to evaluate the safety.
- Review safety Data and will make an assessment of the safety profile and of the benefit risk ratio

- Review each event that could modify the benefit risk ratio of using the study drug in the indication studied (i.e. scientific, safety, ethics events).

The members will receive all the data needed for the evaluation. The data should remain confidential.
The role of the DSMB is consultative, the committee should inform the sponsor about their recommendations. The sponsor decided to follow or not the DSMB advices.
Disagreement between the DSMB and the sponsor should be notified in the final publication.
No fees will be allowed for the DSMB members.
Additional meetings may be called at any time if an event occurs or on request by one or more members.
The DSMB will be regularly informed of the study, and of any emergent problems.

17.1.3. Centralized review Committee

The committee review will be composed of three experts/investigators involved in the study: the coordinating investigator and two surgeons.
The members will meet in person or via teleconference to review the anonymized operative and pathological reports at screening, at the IDS and Other Debulking Surgery of all patients. The aim of this review is to reach a consensus on the data to extract from reports.

18. DATA QUALITY CONTROL

For the study, the site will transcribe data on the e-CRF.
The monitoring will be performed, in accordance to the GCP and FDA guidance (august 2013) on the «risk based approach to monitoring».
A comprehensive validation check program will be generated according to the Data ValidationPlan in order to verify the data.
The investigator or designee will ensure discrepancies resolution through queries.

19. MONITORING OF RESEARCH

In order to guarantee the authenticity of the data in accordance to the GCP, and following the FDA guidance from august 2013 on the «risk-based approach to monitoring», the sponsor establishes a quality insurance system which involves:

- The management of the monitoring committees of the trial, of the pharmacovigilance and of the DSMB,
- The data quality control (for all data from sites) with check of:
  - The compliance with the protocol,
  - The inform consent and of the eligibility of patient participating in the trial,
  - The data consistency of the eCRF,
  - Each SAE notification,
  - The traceability of the medicinal product

An audit of participating sites can be set up if this measure is deemed necessary.

The CRA will be provided by the sponsor. Patient's data must be accessible to them to carry out the mission and are bound by their duty of professional secrecy in accordance to the penal code regulation.
Written report publication is mandatory to ensure the monitoring traceability.
A monitoring plan will be establish according to the «risk based approach to monitoring».

According to the ICH Guidelines of Good Clinical Practice, the study will be monitored on a regular basis. The applicable directives for data protection law will be kept. It is the responsibility of the clinical monitor to follow the study via telephone contact, written correspondence, and regular visits to the Investigator and study sites to review records and drug supplies. The clinical monitor will maintain current, personal knowledge of the study through observation, review of the records, comparison with source documents, and discussion of the conduct of the study with the investigators.

20. AUDITS AND INSPECTIONS

The investigator should understand that source documents for this trial should be made available to authorized representatives of the sponsor and the regulatory agency(s) after appropriate notification. The verification of the eCRF data must be by direct inspection of source documents. This includes examining, analyzing, verifying, and reproducing any records and reports that are important to the evaluation of the study. The investigator is responsible for giving any requested support for any inspection or audit visit and has to be available during these visits. In case of audits or inspections a direct access to the eCRF will be provided.

21. ETHICAL, LEGISLATIVE AND REGULATORY CONSIDERATIONS

21.1.1. Ethical considerations

The study will be performed in accordance with ethical principles that have their origin in the Declaration of Helsinki and are consistent with International Conference on Harmonization (ICH)/Good Clinical Practice (GCP) and the applicable regulatory requirements. A special emphasis will be placed on data protection.
As this clinical study will be carried out in France only, the study will be conducted in accordance with the "Code de la Santé Publique" and data collection and recording will be done in accordance with the "Méthodologie de Référence MR-001" of the Commission Nationale Informatique et Libertés.

21.1.2. Sponsor responsibilities

Main responsabilities of the sponsor are:

- The subscription of a civil liability insurance,
- Obtaining EudraCT number and registration of the clinical trial on the European Database (European Drug Regulatory Authorities Clinical Trials),
- The registration of the clinical trial on Clinical.gov
- Obtaining authorization of the clinical trial initial project and possible amendment from the ethic committee and the competent authorities: ethic committee favourable opinion and competent authority's approval.
- The declaration to competent authorities and in the EudraVigilance database of any suspicious suspected unexpected serious adverse reaction and forward to the ethic committee and to the trial investigators,
- The transmission of the annual safety report to the ethic committee and the competent authority,
- The information about the trial to the Directors, pharmacists and investigators,
- The declaration of the first inclusion and the end of inclusion period to the ethic committee and to the competent authorities,
- Writing of the final report and transmission of the synopsis to the competent authorities,
- Trial results information to the ethic committee, to the competent authorities and to the research participant,
- Archiving of essential documents in the sponsor trial master file for a minimum of 15 years after the end of the trial.

21.1.3. Investigator responsibilities

The principal investigator of each site must conduct the clinical trial in accordance with the research protocol and with current regulations.

The investigator should not implement any deviation from, or changes of the protocol without authorized in writing by the sponsor and without previous ethic committee and competent authorities' authorization of proposed changes.

The principal investigator is responsible for:

- Respect the trial confidentiality,
- Give his curriculum vitae and those of the co-investigators,
- Identify the members of the team who participate to the study and define their role and responsibilities,
- Start the patient recruitment after sponsor authorization,
- To make himself available for the monitoring visit and for investigator meetings.

It is the responsibility of each investigator to:

- Respect the trial confidentiality,
- Collect informed consent that should be signed and personally dated by patients before any procedure for selecting,
- Regularly complete the eCRF of each patient included and give an access to source document to the CRA provided by the sponsor in order to validate data,
- Notify as soon as possible any SAE and event of particular interest arising in the course of the research,
- Accept regular monitoring visit or calls of the CRA and possibly by auditors mandated by the sponsor or by the competent authorities, and give direct access to source document about patients (medical or hospital record, nursing follow-up, results of additional tests …)

Clinical investigators must give patient's files direct access to the CRA and the competent authorities

21.1.4. Informed consent

The informed consent form must include all elements required by the International Conference on Harmonization (ICH), Good Clinical Practice (GCP) and applicable regulatory requirements and must adhere to the ethical principles that have their origin in the Declaration of Helsinki. The ICF will be approved by the Ethics committee. (previously reviewed by the patient committee of ligue nationale contre le cancer)

Prior to patient participation in the trial, written informed consent must be obtained from each patient according to ICH GCP and to the regulatory and legal requirements.

The investigator or his/her entitled designee (as defined on the delegation list) must provide the participant with a copy of the ICF and written information about the study in the language in which the participant is most proficient. The language must be nontechnical and easily understood.

The investigator will be available to answer questions regarding procedures, risks and alternatives, after which the ICF must be signed and personally dated by the participant and by the person who conducted the informed consent discussion. The participant must receive a hard copy of the signed ICF.

The inform consent will be reviewed each time a new information is available on the protocol and/or the informed consent if the informed consent form is impact by this new information.

Patient can refuse to participate in the study or withdrawn her consent at any time during the protocol without any impact for her care.

22. PROCESSING OF DATA AND STUDY DOCUMENTS ARCHIVING

22.1.1. eCRF completion

Data will be captured via electronic data capture.
The investigator will approve the data with using electronic signature, and this approval is used to confirm the accuracy of the data recorded.

The investigator's data documented in eCRF will be accessible from the investigator's site throughout the trial. Relevant medical history prior to enrolment will be documented at the baseline visit. Thereafter during the trial, narrative statements relative to the patient's progress during the trial will be maintained. The eCRFs must be kept current to reflect patient status at each phase during the course of the trial. The patients must not be identified on the eCRF by name. Appropriate coded identification (i.e. Patient Number) must be used. The investigator must make a separate confidential record of these details (patient identification code list) to permit identification of all patients enrolled in a clinical trial in case follow-up is required.

If corrections are needed, it will be done by the investigator or designee team member in the following manner:

- Accurate data shall be drawn through remaining readable, and the correct data must be written close to it.
- Corrections must be certified. For SAE corrections or main efficiency variable, reason for correction must be done.

During the trial, Data Clarification Form (DCFs) could be sent to validate coherence of data. It will need to be complemented as the same manner by investigator or designee.

22.1.2. Source documents

Source documents provide evidence for the existence of the patient and substantiate the integrity of the data collected. Source documents are filed at the investigator's site.
Data entered in the eCRFs that are transcribed from source documents must be consistent with the source documents or the discrepancies must be explained. The investigator may need to request previous medical records or transfer records, depending on the trial; also, current medical records must be available. For eCRFs all data must be derived from source documents.
Data on the quality of life questionnaire are considered source data and have to be stored along with the patient file. The data of the quality of life questionnaire will be entered into the data base by the site.

22.1.3. Direct access to source data-documents

The investigator/institution will permit trial-related monitoring, audits, IRB/IEC review and regulatory inspection, providing direct access to all related source data/documents.
Electronic CRFs and all source documents, including progress notes and copies of laboratory and medical test results must be available at all times for review by the sponsor's clinical trial monitor, auditor and inspection by health authorities. The Clinical Research Associate (CRA)/on site monitor and auditor may review all eCRFs, and written informed consents. The accuracy of the data will be verified by reviewing the documents described in Section above (22.1.1).

22.1.4. Maintenance of records

The investigator shall maintain the records of drug disposition, final eCRFs, worksheets and all other study-specific

documentation (eg, study file notebooks and source documentation) until notified by the Sponsor that records may be destroyed. If the application is not filed or is withdrawn, the investigator shall maintain the records for at least 15 years after completion or discontinuation of the study. After that period of time the documents may be destroyed, subject to local regulations.

To avoid error, the investigator should contact the Sponsor or his representative before the destruction of any records pertaining to the study to ensure they no longer need to be retained.

23. CONIDENTIALITY OF TRIAL DOCUMENTS AND PATIENT RECORDS

The investigator must ensure that patients' anonymity will be maintained and that their identities are protected from unauthorised parties. On eCRFs or other documents submitted to the sponsor, patients should not be identified by their names, but by an identification code. The investigator should keep a patient enrolment log showing codes, names and addresses. The investigator should maintain documents e.g., patients' written consent forms, in strict confidence, at the site.
The investigator should understand that source documents for this trial should be made available to appropriately qualified personnel from the sponsor or to health authority inspectors after appropriate notification.

24. OWNERSHIP OF DATA

ARCAGY-GINECO will be the owner of all data generated from the Study Research.

25. PUBLICATIONS

The biostatistician(s) will compile a final report. It will include tables giving the raw data and the statistical report on the data.
The publication of the final report of the results of the study shall be in accordance with the protocol, and the GINECO publication guidelines.

General Policy:

Authorships and Co-authorships are not granted by individual but by institutions or consortia of study centers. All calculations regarding the number and position of co-authorships will be based on numbers of recruited patients by institution. Each institution is completely free and independent to fill in individual names according to its number and position of co-authorships (the group even may appoint persons not having recruited patients by themselves). The following "rules" should guarantee participation and benefits for all institutions involved and share as much attractive positions among the institutions as possible.

Fixed authorship positions:

All co-authorship positions depend on recruitment of groups except one authorship position of the chairman and one for the statistician (4th position). The chairman's position is first author or senior author depending on the occasion unless he grants this to anyone else.

Flexible co-authorship positions:

The specific place of the group's representative is defined by the overall recruitment by the institution. The number of authors per institution will also depend on the overall recruitment by the institution and will be defined by the steering committee.

Additional publications of subgroup data or sub-projects:

First author should be of the institution performing the analysis. The 2nd, 3rd position and senior author are also granted to the institution performing subprojects. Other institutions should be mentioned and have co-authorship positions similar to the rules for primary and main publication. PI usually senior author. All sub-publications or meta-analyses can only be published after the full manuscript of the study has been published.
Full paper on general analyses of secondary endpoints (eg quality of life etc.) should be shared among the institutions with first author by institution A, then 2nd general paper first author by institution B etc.

Presentations:

The study should be presented as often as possible to give as many institutions as possible the opportunity to present. Local and national presentations should be done by the national institutions as first author (with mentioning all other groups, PI usually senior author).

26. REFERENCES

1. Gatta G, van der Zwan JM, Casali PG, Siesling S, Dei Tos, AP, Kunkler I, Otter R, Licitra L, Mallone S, Tavilla A, Trama A, and Capocaccia R. **Rare cancers are not so rare: the rare cancer burden in Europe.** *Eur. J. Cancer* 2011; 47(17):2493-2511.
2. Jemal A, Siege l R, Ward E, Hao Y, Xu J, Murray T, and Thun MJ. **Cancer statistics.** *CA. Cancer J. Clin.* 2008; 58(2): 71-96.
3. Cannistra SA (2004). Cancer of the ovary. *The New England journal of medicine* **351**(24)**:** 2519-2529.
4. Vergote I, Tropé CG, Amant F, Kristensen G, Ehlen T, Johnson N, Verheijen R, van der Burg M, Lacave A, Benedetti Panici P, Kenter G, Casado A, Mendiola C, Coens C, Verleye L, Stuart G, Pecorelli S, Reed N. **Neoadjuvant Chemotherapy or Primary Surgery in Stage IIIC or IV Ovarian Cancer.** *N Engl J Med* 2010; 363(10): 943-53.
5. Du Bois A, Reuss A, Pujade-Lauraine E, Harter P, Ray-Coquard I, Pfisterer J. **Role of surgical outcome as prognostic factor in advanced epithelial ovarian cancer: a combined exploratory analysis of 3 prospectively randomized phase 3 multicenter trials: by the Arbeitsgemeinschaft Gynaekologische Onkologie Studiengruppe Ovarialkarzinom (AGO-OVAR) and the Groupe d'Investigateurs Nationaux Pour les Etudes des Cancers de l'Ovaire (GINECO).** *Cancer* 2009 Mar 15;115(6):1234-44.
6. Vergote I, Trope CG, Amant F, Ehlen T, Reed NS, Casado A Neoadjuvant chemotherapy is the better treatment option in some patients with stage IIIc to IV ovarian cancer. *Journal of clinical oncology : official journal of the American Society of Clinical Oncology* (2011). **29**(31)**:** 4076-4078.
7. Stuart GC1, Kitchener H, Bacon M, duBois A, Friedlander M, Ledermann J, Marth C, Thigpen T, Trimble E; participants of 4th Ovarian Cancer Consensus Conference (OCCC); Gynecologic Cancer Intergroup.
8. Kang S, Nam BH. **Does neoadjuvant chemotherapy increase optimal cytoreduction rate in advanced ovarian cancer? Meta-analysis of 21 studies**. *Ann Surg Oncol.* 2009 Aug;16(8):2315-20
9. Bristow RE, Tomacruz RS, Armstrong DK, Trimble EL, Montz FJ. **Survival effect of maximal cytoreductive surgery for advanced ovarian carcinoma during the platinum era: a meta-analysis.** *Journal of clinical oncology : official journal of the American Society of Clinical Oncology* 2002. 20(5): 1248-1259.
10. Bristow RE, Chi DS. **Platinum-based neoadjuvant chemotherapy and interval surgical cytoreduction for advanced ovarian cancer: a meta-analysis.** *Gynecologic oncology 2006.* **103**(3)**:** 1070-1076.
11. Ferron JG, Uzan C, Rey A, Gouy S, Pautier P, Lhomme C*, et al.* (2009**). Histological response is not a prognostic factor after neoadjuvant chemotherapy in advanced-stage ovarian cancer with no residual disease.** *European journal of obstetrics, gynecology, and reproductive biology* **147**(1)**:** 101-105.
12. Hoskins PJ. **Which is the better surgical strategy for newly diagnosed epithelial ovarian cancer: primary or interval debulking?** *Curr Opin Oncol.* 2011 Sep;23(5):501-6.
13. Onda T, Yoshikawa H. **Neoadjuvant chemotherapy.** *Expert Rev Anticancer Ther.* 2011 Jul;11(7):1055-69.
14. Stoeckle E, Bourdarias L, Guyon F, Croce S, Brouste V, Thomas L, Floquet A. **Progress in survival outcomes in patients with advanced ovarian cancer treated by neo-adjuvant platinum/taxane-based chemotherapy and late interval debulking surgery.** *Ann Surg Oncol.* 2014 Feb;21(2):629-36.
15. **19th International Meeting of the European Society of Gynecological Oncology (ESGO 2015).***Int J Gynecol Cancer.* 2015 Oct;25(9 Suppl 1)
16. Querleu D, Rafii A, Colombo PE, Ferron G, Rouanet P, Martinez A. **Randomized study of aggressive surgery for advanced ovarian cancer.** *Int J Gynecol Cancer.* 2013 Sep;23(7):1170.
17. McGuire WP1, Hoskins WJ, Brady MF, Kucera PR, Partridge EE, Look KY, Clarke-Pearson DL, Davidson M. **Cyclophosphamide and cisplatin compared with paclitaxel and cisplatin in patients with stage III and stage IV ovarian cancer.** *N Engl J Med.* 1996 Jan 4;334(1):1-6.
18. Burger RA, Brady MF, Bookman MA, Fleming GF, Monk BJ, Huang H, Mannel RS, Homesley HD, Fowler J, Greer BE, Boente M, Birrer MJ, and Liang SX. **Incorporation of bevacizumab in the primary treatment of ovarian cancer.** *N. Engl. J. Med.* 2011; 365(26):2473-2483.
19. Perren TJ, Swart AM, Pfisterer J, Ledermann JA, Pujade-Lauraine E, Kristensen G, Carey MS, Beale P, Cervantes A, Kurzeder C, du Bois A, Sehouli J, Kimmig R, Stahle A, Collinson F , Essapen S, Gourley C , Lortholary A, Selle F, Mirza,MR, Leminen A, Plante M, Stark D, Qian W, Parmar MK, and Oza AM**. A phase 3 trial of bevacizumab in ovarian cancer.** *N. Engl. J. Med.* 2011; 365(26), 2484-2496.
20. Kristensen G, Perren T, Qian W, Pfisterer J, Ledermann JA, Joly F, Carey MS, Beale PJ, Cervantes A, Oza AM. **Result of interim analysis of overall survival in the GCIG ICON7 phase III randomized trial of bevacizumab in women with newly diagnosed ovarian cancer**. *J Clin Oncol* 29: 2011 (abstr LBA5006).
21. Zhang L, Conejo-Garcia JR, Katsaros D, Gimotty PA, Massobrio M, Regnani G, Makrigiannakis A, Gray H, Schlienger K, Liebman MN, Rubin SC, Coukos G. **Intratumoral T cells, recurrence, and survival in epithelial ovarian cancer.** *N Engl J Med. 2003* Jan 16;348(3):203-13.
22. Lavoué V et al. **Immunity of human epithelial ovarian carcinoma: the paradigm of immune suppression in cancer.** *J Transl Med. 2013* Jun 13;11:147.

23. Apolo AB, Vogelzang NJ, Theodorescu D. **New and promising strategies in the management of bladder cancer**. Am Soc Clin Oncol Educ Book. 2015:105-12. doi:10.14694/EdBook_AM.2015.35.105. Review. PubMed PMID: 25993148.
24. Shah CA, Allison KH, Garcia RL, Gray HJ, Goff BA, Swisher EM. **Intratumoral T cells, tumor-associated macrophages, and regulatory T cells: association with p53 mutations, circulating tumor DNA and survival in women with ovarian cancer.** *Gynecol Oncol*. 2008 May;109(2):215-9
25. Zhang QW, Liu L, Gong CY, Shi HS, Zeng YH, Wang XZ, Zhao YW, Wei YQ. **Prognostic significance of tumor-associated macrophages in solid tumor: a meta-analysis of the literature.** *PLoS One.* 2012;7(12):e50946.
26. Labidi-Galy SI, Sisirak V, Meeus P, Gobert M, Treilleux I, Bajard A, Combes JD, Faget J, Mithieux F, Cassignol A, Tredan O, Durand I, Ménétrier-Caux C, Caux C, Blay JY, Ray-Coquard I, Bendriss-Vermare N. **Quantitative and functional alterations of plasmacytoid dendritic cells contribute to immune tolerance in ovarian cancer.** *Cancer Res.* 2011 Aug 15;71(16):5423-34. doi: 10.1158/0008-5472.CAN-11-0367. Epub 2011 Jun 22.
27. Hamid O, Robert C, Daud A, Hodi FS, Hwu WJ, Kefford R, et al. **Safety and tumor responses with lambrolizumab anti-PD-1- in melanoma.** N Engl J Med 2013;369:134-44.
28. Hodi FS, Lee S, McDermott DF, Rao UN, Butterfield LH, Tarhini AA, Leming P, Puzanov I, Shin D, Kirkwood JM **Ipilimumab plus sargramostim vs ipilimumab alone for treatment of metastatic melanoma: a randomized clinical trial.** *JAMA*. 2014 Nov 5;312(17):1744-53.
29. Robert C, Mateus C. **Drug therapy of melanoma: anti-CTLA-4 and anti-PD-1 antibodies.** *Bull Acad Natl Med.* 2014 Feb;198(2):297-308.
30. Topalian SL et al. **Safety, activity, and immune correlates of anti–PD-1 antibody in cancer.** *N Engl J Med.* 2012; 366:2443–54.
31. Wolchok JD et al. **Nivolumab plus ipilimumab in advanced melanoma.** *N Engl J Med.* 2013; 369:122–33.
32. Brahmer JR et al. **Safety and activity of anti-PD-L1 antibody in patients with advanced cancer.** *N Engl J Med.* 2012; 366:2455–65.
33. Disis ML. Immune Regulation of Cancer. J Clin Oncol 2010;28:4531-8.
34. Dudley ME, Wunderlich JR, Yang JC, Sherry RM, Topalian SL, Restifo NP, et al. **Adoptive cell transfer therapy following non-myeloablative but lymphodepleting chemotherapy for the treatment of patients with refractory metastatic melanoma.** *J Clin Oncol* 2005;23(10):2346–57.
35. Hunder NN, Wallen H, Cao J, Hendricks DW, Reilly JZ, Rodmyre R, et al. **Treatment of metastatic melanoma with autologous CD4+ T-cells against NY-ESO-1**. *N Engl J Med* 2008;358:2698-703.
36. Greenwald RJ, Freeman GJ, Sharpe AH. **The B7 family revisited.** *Annu Rev Immunol* 2005;23:515-48.
37. Okazaki T, Maeda A, Nishimura H, Kurosaki T, Honjo T. **PD-1 immunoreceptor inhibits B cell receptor-mediated signaling by recruiting src homology 2-domaincontaining tyrosine phosphatase 2 to phosphotyrosine.** *Proc Natl Acad Sci USA* 2001;98:13866-71.
38. Zhang X, Schwartz JC, Guo X, Bhatia S, Cao E, Lorenz M, et al. **Structural and functional analysis of the costimulatory receptor programmed death-1**. *Immunity* 2004;20:337-47.
39. Chemnitz JM, Parry RV, Nichols KE, June CH, Riley JL. **SHP-1 and SHP-2 associate with immunoreceptor tyrosine-based switch motif of programmed death 1 upon primary human T-cell stimulation, but only receptor ligation prevents T-cell activation.** *J Immunol* 2004;173:945-54.
40. Sheppard KA, Fitz LJ, Lee JM, Benander C, George JA, Wooters J et al. **PD-1 inhibits T-cell receptor induced phosphorylation of the ZAP70/CD3zeta signalosome and downstream signaling to PKCtheta.** *FEBS Lett* 2004;574:37-41.
41. Riley JL. **PD-1 signaling in primary T-cells.** *Immunol Rev* 2009;229:114-25.
42. Parry RV, Chemnitz JM, Frauwirth KA, Lanfranco AR, Braunstein I, Kobayashi SV, et al. **CTLA-4 and PD-1 receptors inhibit T-cell activation by distinct mechanisms.** *Mol Cell Biol* 2005;25:9543-53.
43. Francisco LM, Sage P, Sharpe AH. **The PD- -1 pathway in tolerance and autoimmunity**. *Immunol Rev* 2010;236:219-42.
44. Agata Y, Kawasaki A, Nishimura H, Ishida Y, Tsubata T, Yagita H, et al. **Expression of the PD-1 antigen on the surface of stimulated mouse T and B lymphocytes.** *Int Immunol* 1996;8(5):765-72.
45. Vibhakar R, Juan G, Traganos F, Darzynkiewicz Z, Finger LR. **Activation induced expression of human programmed death-1 gene in T-lymphocytes.** *Exp Cell Res* 1997;232:25-8.
46. Nishimura H, Honjo T, Minato N. **Facilitation of beta selection and modification of positive selection in the thymus of PD-1-deficient mice.** *J Exp Med* 2000;191:891-8.
47. Brown JA, Dorfman DM, Ma FR, Sullivan EL, Munoz O, Wood CR, et al. **Blockade of programmed death-1 ligands on dendritic cells enhances T-cell activation and cytokine production.** *J Immunol* 2003;170:1257-66.
48. Dong H, Strome SE, Salomao DR, Tamura H, Hirano F, Flies DB, et al. **Tumorassociated B7-H1 promotes T-cell apoptosis: a potential mechanism of immune evasion.** *Nat Med* 2002;8:793-800.
49. 2-17 Sharpe AH, Freeman GJ. The B7-CD28 superfamily. Nat Rev Immunol 2002;2:116-26.
50. Fourcade J, Kudela P, Sun Z, Shen H, Lenzner D, Guillaume P, et al. **PD-1 is a regulator of NY-ESO-1-specific CD8+ T-cell expansion in melanoma patients.** *J Immunol* 2009;182:5240-9.
51. Jinushi M and Dranoff G. **Triggering Tumor Immunity through Angiogenesis Targeting**. Clin Cancer Res 2007;13:3762-3764.

52. Shrimali RK et al. **Antiangiogenic agents can increase lymphocyte infiltration into tumor and enhance the effectiveness of adoptive immunotherapy of cancer**. Cancer Res. 2010 August 1; 70(15): 6171–6180.
53. Roland CL et al. **Cytokine Levels Correlate with Immune Cell Infiltration after Anti-VEGF Therapy in Preclinical Mouse Models of Breast Cancer**. PLoS One. 2009 Nov 3;4(11):e7669.
54. Roland CL et al. **Cytokine Levels Correlate with Immune Cell Infiltration after Anti-VEGF Therapy in Preclinical Mouse Models of Breast Cancer**. PLoS One. 2009 Nov 3;4(11):e7669.
55. **An engineered PD-L1 antibody, in patients with metastatic renal cell carcinoma (mRCC**). ESMO 2014 Madrid.
56. McDermott DF et al. **A Phase 2 Study of MPDL3280A (an Engineered Anti-PDL1 Antibody) as Monotherapy or in Combination With Avastin (Bevacizumab) Compared to Sunitinib in Patients With Untreated Advanced Renal Cell Carcinoma**. http://clinicaltrials.gov/ct2/show/NCT01984242
57. Lieu C et al. **A Phase 1b Study of MPDL3280A (an Engineered Anti-PDL1 Antibody) in Combination With Avastin (Bevacizumab) and/or With Chemotherapy in Patients With Locally Advanced or Metastatic Solid Tumors** http://www.clinicaltrials.gov/show/NCT01633970
58. Arkadiusz Z. Dudek, R. Alejandro Sica, Amer Sidani, Gautam Jha, Hui Xie, Ajjai Alva, Mark Stein, and Eric A. Singer. **Phase Ib Study of Pembrolizumab in Combination with Bevacizumab for the Treatment of Metastatic Renal Cell Carcinoma**: Big Ten Cancer Research Consortium BTCRC-GU14-003.
59. Kryczek I, Liu R, Wang G, Wu K, Shu X, Szeliga W, Vatan L, Finlayson E, Huang E, Simeone D, Redman B, Welling TH, Chang A, Zou W. **FOXP3 defines regulatory T cells in human tumor and autoimmune disease.** Cancer Res. 2009 May 1;69(9):3995-4000.
60. Hamanishi J1, Mandai M, Iwasaki M, Okazaki T, Tanaka Y, Yamaguchi K, Higuchi T, Yagi H, Takakura K, Minato N, Honjo T, Fujii S. **Programmed cell death 1 ligand 1 and tumor-infiltrating CD8+ T lymphocytes are prognostic factors of human ovarian cancer.** Proc Natl Acad Sci U S A. 2007 Feb 27;104(9):3360-5.
61. Weber et al. Results of a Phase III Randomised Study of Nivolumab in Patients with Advanced Melanoma After Prior Anti-CTLA4 Therapy. ESMO 2014
62. Antoni Ribas FSH, Richard Kefford, Omid Hamid, Adil Daud, Jedd D. Wolchok, Wen-Jen Hwu, Tara C. Gangadhar, Amita Patnaik, Anthony M. Joshua, Peter Hersey, Jeffrey S. Weber, Roxana Stefania Dronca, Hassane M. Zarour, Kevin Gergich, Xiaoyun (Nicole) Li, Robert Iannone, Soonmo Peter Kang, Scot Ebbinghaus, Caroline Robert; David Geffen. **Efficacy and safety of the anti-PD-1 monoclonal antibody MK-3475 in 411 patients (pts) with melanoma (MEL)**. J Clin Oncol 32:5s 2014; (suppl; abstr LBA9000^) and ASCO 2015
63. Steffen Böhm, Asma Faruqi, Ian Said, Michelle Lockley, Elly Brockbank, Arjun Jeyarajah, Amanda Fitzpatrick,Darren Ennis, Thomas Dowe, Jennifer L. Santos, Linda S. Cook, Anna V. Tinker, Nhu D. Le,C. Blake Gilks, and Naveena Sing. **Chemotherapy Response Score: Development and Validation of a System to Quantify Histopathologic Response to Neoadjuvant Chemotherapy in Tubo-Ovarian High-Grade Serous** Carcinoma. J Clin Oncol. 2015 Aug 1;33(22):2457-63. doi: 10.1200/JCO.2014.60.5212. Epub 2015 Jun 29.
64. Aaronson NK, Ahmedzai S, Bergman B, Bullinger M, Cull A, Duez NJ, Filiberti A, Flechtner H, Fleishman SB, de Haes JC, et al. **The European Organization for Research and Treatment of Cancer QLQ-C30: a quality-of-life instrument for use in international clinical trials in oncology.** J Natl Cancer Inst. 1993 Mar 3;85(5):365-76.
65. Fayers PM. **Interpreting quality of life data: population-based reference data for the EORTC QLQ-C30.** Eur J Cancer. 2001 Jul;37(11):1331-4
66. Greimel E, Bottomley A, Cull A, Waldenstrom AC, Arraras J, Chauvenet L, Holzner B, Kuljanic K, Lebrec J, D'haese S, EORTC Quality of Life Group and the Quality of Life Unit. **An international field study of the reliability and validity of a disease-specific questionnaire module (the QLQ-OV28) in assessing the quality of life of patients with ovarian cancer**. Eur J Cancer. 2003 Jul;39(10):1402-8.
67. Osoba D.**Health-related quality of life and cancer clinical trials**. Ther Adv Med Oncol. 2011 Mar;3(2):57-71. doi: 10.1177/1758834010395342.
68. Bonnetain F1, Dahan L, Maillard E, Ychou M, Mitry E, Hammel P, Legoux JL, Rougier P, Bedenne L, Seitz JF. **Time until definitive quality of life score deterioration as a means of longitudinal analysis for treatment trials in patients with metastatic pancreatic adenocarcinoma**. Eur J Cancer. 2010 Oct;46(15):2753-62. doi: 10.1016/j.ejca.2010.07.023. Epub 2010 Aug 17.
69. Luyckx M[1], Leblanc E, Filleron T, Morice P, Darai E, Classe JM, Ferron G, Stoeckle E, Pomel C, Vinet B, Chereau E, Bergzoll C, Querleu D. **Maximal cytoreduction in patients with FIGO stage IIIC to stage IV ovarian, fallopian, and peritoneal cancer in day-to-day practice: a Retrospective French Multicentric Study.**

27. APPENDIX

APPENDIX 1 - ECOG PERFORMANCE STATUS

Description Scale

0	Fully active, able to carry on all pre-disease performance without restriction
1	Restricted in physically strenuous activity but ambulatory and able to carry out work of a light or sedentary nature, e.g., light house work, office work
2	Ambulatory and capable of all selfcare but unable to carry out any work activities. Up and about more than 50% of waking hours
3	Capable of only limited selfcare, confined to bed or chair more than 50% of waking hours
4	Completely disabled. Cannot carry on any selfcare. Totally confined to bed or chair

Oken MM, Creech RH, Tormey DC, et al. Toxicity And Response Criteria Of The Eastern Cooperative Oncology Group. Am J Clin Oncol 1982; 5(6):649-655

Appendix 1 bis:

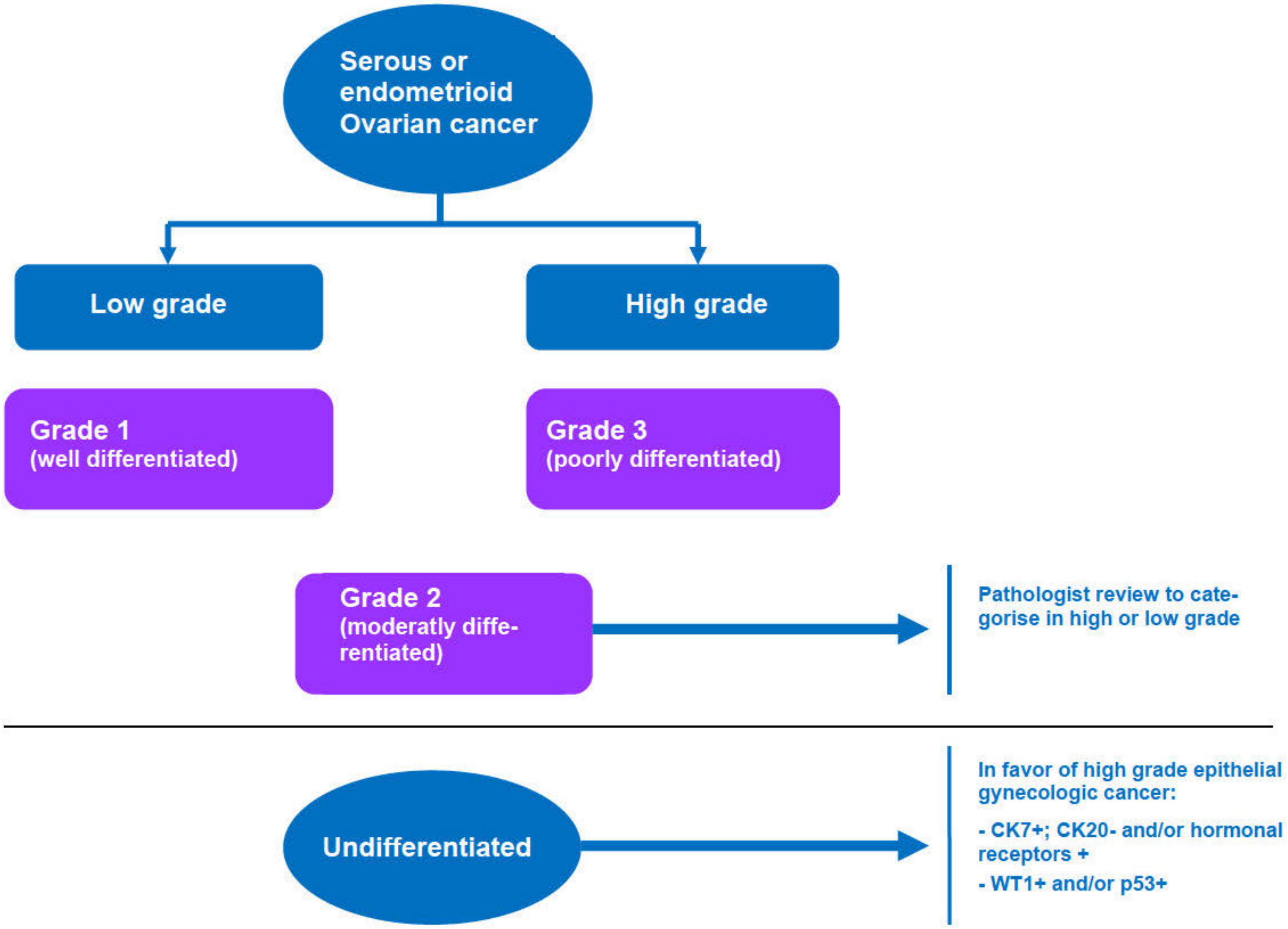

APPENDIX 2 – FIGO STAGING 2014

FIGO Guidelines: Staging classification for cancer of the ovary, fallopian tube, and peritoneum. Jaime Prat for the FIGO Committee on Gynecologic Oncology. International Journal of Gynecology & Obstetrics. Vol 124, (1), January 2014, 1–5

FIGO (2014) Ovary, Fallopian tube, Peritoneum Cancer	Stage		Stage
Descriptor			
I: Tumor confined to ovaries or fallopian tube(s)	I	III: Tumor involves 1 or both ovaries or fallopian tubes, or primary peritoneal cancer, with cytologically or histologically confirmed spread to the peritoneum outside the pelvis and/or metastasis to the retroperitoneal lymph nodes	III
IA: Tumor limited to 1 ovary (capsule intact) or fallopian tube; no tumor on ovarian or fallopian tube surface; no malignant cells in the ascites or peritoneal washings	IA	IIIA1: Positive retroperitoneal lymph nodes only (cytologically or histologically proven)	IIIA1
IB: Tumor limited to both ovaries (capsules intact) or fallopian tubes; no tumor on ovarian or fallopian tube surface; no malignant cells in the ascites or peritoneal washings	IB	IIIA1(i) Metastasis up to 10 mm in greatest dimension	IIIA1(i)
IC: Tumor limited to 1 or both ovaries or fallopian tubes, with any of the following	IC	IIIA1(ii) Metastasis more than 10 mm in greatest dimension	IIIA1(ii)
IC1: Surgical spill	IC1	IIIA2: Microscopic extrapelvic (above the pelvic brim) peritoneal involvement with or without positive retroperitoneal lymph nodes	IIIA2
IC2: Capsule ruptured before surgery or tumor on ovarian or fal- lopian tube surface	IC2	IIIB: Macroscopic peritoneal metastasis beyond the pelvis up to 2 cm in greatest dimension, with or without metastasis to the retroperitoneal lymph nodes	IIIB
IC3: Malignant cells in the ascites or peritoneal washings	IC3	IIIC: Macroscopic peritoneal metastasis beyond the pelvis more than 2 cm in greatest dimension, with or without metastasis to the retro- peritoneal lymph nodes (includes extension of tumour to capsule of liver and spleen without parenchymal involvement of either organ)	IIIC
II: Tumor involves 1 or both ovaries or fallopian tubes with pelvic extension (below pelvic brim) or primary peritoneal cancer	II	IV: Distant metastasis excluding peritoneal metastases	IV
IIA: Extension and/or implants on uterus and/or fallopian tubes and/ or ovaries	IIA	IVA: Pleural effusion with positive cytology	IVA
IIB: Extension to other pelvic intraperitoneal tissues	IIB	IVB: Parenchymal metastases and metastases to extra- abdominal or- gans (including inguinal lymph nodes and lymph nodes outside of the ab- dominal cavity)	IVB

APPENDIX 3 – PERITONEAL CANCER INDEX (PCI) OF RESIDUAL TUMOR AND COMPLETENESS OF CYTOREDUCTION INDEX (CCI)

Regions	Lesions size
0 Central	…….
1 Right upper	…….
2 Epigastrium	…….
3 Left Upper	…….
4 Left Flank	…….
5 Left Lower	…….
6 Pelvis	…….
7 Right Lower	…….
8 Right Flank	…….
9 Upper jejunum	…….
10 Lower jejunum	…….
11 Upper Ileum	…….
12 Lower Ileum	…….
**PCI**	

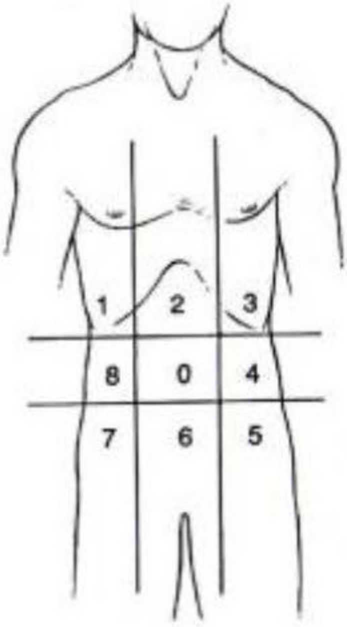

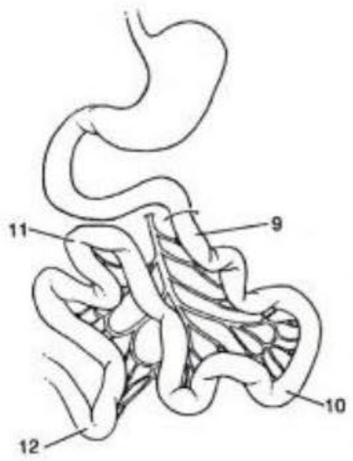

Lesion Size Score	
LS0	No tumor seen
LS1	Tumor up to 0.5cm
LS2	Tumor up to 5.0 cm
LS3	Tumor> 5.0 cm or confluence

Completeness of Cytoreduction Index (CCI)

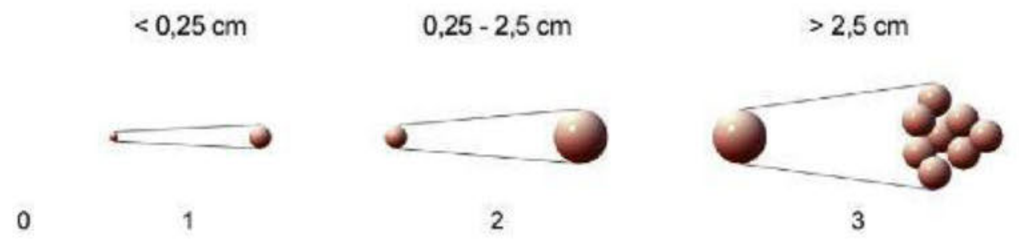

Resection can be classified as complete or incomplete, as follow:

- CC0: no macroscopic residual disease
- CC1: residual disease < 0,25 cm
- CC2: residual disease 0,25<R<2.5cm
- CC3: residual disease >2. 5 cm.

Score coelioscopique de Fagotti (2008)

- Atteinte massive du grand épiploon
- Carcinose péritonéale
- Carcinose diaphragmatique
- Rétraction mésentérique
- Infiltration de l'estomac
- Métastases hépatiques

Chaque item est coté de 0 à 2 , la chirurgie d'exérèse est incomplète chez 100% des patientes dont le score est ≥à 8

APPENDIX 4 – CLAVIEN-DINDO CLASSIFICATION

**Grade**	**Definition**
**Grade I**	Any deviation from the normal course without the need for pharmacological treatment or surgical, endoscopic and radiologic interventions<br>Allowed therapeutic regimens are: drugs as antiemetics, antipyretics, analgetics, diuretics, electrolytes and physiotherapy. This grade also includes wound infections opened at the bedside
**Grade II**	Requiring pharmacological treatment with drugs other than such allowed for grade I complications<br>Blood transfusions and total parenteral nutrition are also included
**Grade III**	Requiring surgical, endoscopic or radiological intervention
III a	Intervention not under general anesthesia
III b	Intervention under general anesthesia
**Grade IV**	Life-threatening complication (including CNS complications)* requiring Intensive Care/Intensive Care Unit management
IV a	Single organ dysfunction (including dialysis)
IV b	Multiorgan dysfunction
**Grade V**	Death of a patient
**Brain hemorrhage, ischemic stroke, subarrachnoidal bleeding, but excluding transient ischemic attacks.*<br>*CNS, central*	

APPENDIX 5 – EVALUATION OF DISEASE UPON RECIST 1.1

1. Definition of measurable lesions and non-measurable lesions

1.1. Criteria for measurable lesions:

- Not previously irradiated
- ≥ 10 mm in the longest diameter at baseline (except lymph nodes which must have short axis ≥ 15 mm)
- Measured with computed tomography (CT) or magnetic resonance imaging (MRI)
- Suitable for accurate repeated measurements

1.2. Criteria for non measurable lesions:

- All other lesions, including small lesions (longest diameter <10 mm or pathological lymph nodes with ≥10 to <15mm short axis at baseline)
- Truly non-measurable lesions include the following:
  - Bone lesions
  - Leptomeningeal disease
  - Ascites
  - Pleural / pericardial effusion
  - Inflammatory breast disease
  - Lymphangitic involvement of skin or lung
  - Abdominal masses/abdominal
  - Organomegaly identified by physical examination that is not measurable by CT or MRI
  - Previously irradiated lesions
  - Skin lesions assessed by clinical examination
  - Brain metastasis

1.3. Special cases:

- Lytic bone lesions or mixed lytic–blastic lesions, with identifiable soft tissue components, can be considered measurable if the soft tissue component meets the definition of measurability. Blastic lesions are considered non-measurable.

- Cystic metastases can be considered measurable lesions if they meet the criteria for measurability from radiological point of view, but if non-cystic lesions are presenting the same patient; these should be selected as target lesions.

2. Method of assessment

The same method of assessment and the same technique should be used to characterize each identified and recorded lesion at baseline and during follow-up visits.

A summary of the methods to be used for RECIST assessment is provided below.

Table 1: Summary of methods of assessment		
**Target lesions**	**Non target lesion**	**New lesions**
CT (pre-ferred)<br>MRI	CT (preferred)<br>MRI<br>Clinical Examination<br>X-ray, Chest x-ray	CT (preferred)<br>MRI<br>Clinical Examination<br>X-ray, Chest x-ray<br>Ultrasound<br>Bone scan<br>FDG-PET (if done at baseline)

3. Tumor response evaluation

3.1. Documentation of target lesions

A maximum of 5 measurable lesions, with a maximum of 2 lesions per organ (including lymph nodes), representative of all lesions involved should be identified as TL at baseline. Target lesions should be selected on the basis of their size (longest diameter for non-nodal lesions or short axis for nodal lesions), but in addition should be those that lend themselves to reproducible repeated measurements. It may be the case that, on occasion, the largest lesion does not lend itself to reproducible measurement in which circumstance the next largest lesion, which can be measured reproducibly, should be selected.

3.1.1. Special cases

- If a lesion has completely disappeared, the longest diameter should be recorded as 0 mm.
- If a TL splits into two or more parts, then record the sum of the diameters of those parts.
- If two or more TL merge then the sum of the diameters of the combined lesion should be recorded for one of the lesions and 0 mm recorded for the other lesion(s).
- If a TL is believed to be present and is faintly seen but too small to measure, a default value of 5mm should be assigned. If an accurate measure can be given, this should be recorded, even if it is below 5mm.
- If a TL cannot be measured accurately due to it being too large, provide an estimate of the size of the lesion.
- When a TL has had any intervention e.g. radiotherapy, embolization, surgery etc., during the study, the size of the TL should still be provided where possible.

3.1.2. Evaluation of target lesions

Table 2 : Summary of target lesions evaluation	
**Complete Response (CR)**	Disappearance of all target lesions since baseline. Any pathological lymph nodes selected as target lesions must have a reduction in short axis to < 10 mm.
**Partial Response (PR)**	At least a 30% decrease in the sum of the diameters of TL, taking as reference the baseline sum of diameters.
**Stable disease (SD)**	Neither sufficient shrinkage to qualify for PR nor sufficient increase to qualify for PD.

**Progressive Disease (PD)**	At least a 20% increase in the sum of diameters of target lesions, taking as reference the smallest sum on study (this includes the baseline sum if that is the smallest on study). In addition to the relative increase of 20%, the sum must also demonstrate an absolute increase of at least 5mm.
**Not Evaluable (NE)**	Only relevant if any of the target lesions were not assessed or not evaluable or had a lesion intervention at this visit. Note: If the sum of diameters meets the progressive disease criteria, progressive disease overrides not evaluable as a target lesion response.

3.2. Documentation of non target lesions

All other lesions (or sites of disease) not recorded as TL should be identified as NTL at baseline. Measurements are not required for these lesions, but their status should be followed at subsequent visits. At each visit an overall assessment of the NTL response should be recorded by the Investigator. This section provides the definitions of the criteria used to determine and record overall response for NTL at the investigational site at each visit.

3.2.1 Evaluation of non target lesions

**Table 3 : Summary of non target lesions evaluation**	
**Complete Response (CR)**	Disappearance of all non-target lesions since baseline. All lymph nodes must be non-pathological in size (< 10 mm short axis).
**Non CR/Non PD**	Persistence of one or more NTL
**Progressive Disease (PD)**	Unequivocal progression of existing non-target lesions.<br>Unequivocal progression may be due to an important progression in one lesion only or in several lesions. In all cases the progression MUST be clinically significant for the physician to consider changing (or stopping) therapy.
**Not Evaluable (NE)**	Only relevant when one or some of the non-target lesions were not assessed and, in the Investigator's opinion, they are not able to provide an evaluable overall non-target lesion assessment at this visit.

3.2. New lesions

Details of any new lesions will also be recorded with the date of assessment. The presence of one or more new lesions is assessed as progression.

A lesion identified at a follow up assessment in an anatomical location that was not scanned at baseline is considered a new lesion and will indicate disease progression.

The finding of a new lesion should be unequivocal: i.e. not attributable to differences in scanning technique, change in imaging modality or findings thought to represent something other than tumour.

If a new lesion is equivocal, for example because of its small size, the treatment and tumour assessments should be continued until the new lesion has been confirmed. If repeat scans confirm there is a new lesion, then the progression date should be declared using the date of the initial scan.

3.3. Symptomatic deterioration

Symptomatic deterioration is not a descriptor of an objective response: it is a reason for stopping study therapy. Patients with ‘symptomatic deterioration’ requiring discontinuation of treatment without objective evidence of disease progression at that time should continue to undergo tumour assessments where possible until objective disease progression is observed.

3.4. Evaluation of overall visit response

Table 7 Overall response

Target lesions	Non-Target Lesions	New Lesions	CA-125	Overall Response
CR	CR	No	Normal	CR
CR	NA	No	Normal	CR
NA	Cr	No	Normal	CR
CR	Non CR/Non PD	No	Any Any	PR
CR	NE	No	Any Any	PR
PR	Non PD or NE	No	Any Any	PR
SD	Non PD or NE	No	Any Any	SD
NA	Non CR/Non PD	No	Any Any	SD (Non CR/Non PD)
NE	Non PD or NE NE	No	Any	NE
NA	Any	No		NE
PD	PD	Yes or No		PD
Any	Any	Yes or No		PD
Any	NA	Yes		PD
NA		No		NED

CR = complete response, PR = partial response, SD = stable disease, PD = progressive disease, NE = not evaluable, NED = no evidence of disease, NA = not applicable (only relevant if there were no TL/NTL at baseline), Normal = less than upper limit of normal

APPENDIX 6 – COMMON TERMINOLOGY CRITERIA FOR ADVERSE EVENTS V 4.03 (CTCAE)

Refer to NCI CTC AE v.4.3 online at the following NCI website: http://evs.nci.nih.gov/ftp1/CTCAE/CTCAE_4.03_2010-06-14_QuickReference_5x7.pdf

Toxicity grade should reflect the most severe degree occurring during the evaluated period, not an average.

When 2 criteria are available for similar toxicities, the one resulting in the more severe grade should be used.

The evaluator must attempt to discriminate between disease/treatment and related signs/symptoms.

An accurate baseline prior to therapy is essential.

NATIONAL CANCER INSTITUTE
Cancer Therapy Evaluation Program

Common Terminology Criteria for Adverse Events v4.03
(Publish Date June 14, 2010)
