# Supplementary material for: Neoadjuvant and adjuvant pembrolizumab in advanced high-grade serous carcinoma: the randomized phase II NeoPembrOV clinical trial
Source: Nat Commun. 2024 Jul 16;15:5931. doi: 10.1038/s41467-024-46999-x (PMC11252284; doi:10.1038/s41467-024-46999-x)
Supplement: Supplementary file 2 — Reporting Summary [file 41467_2024_46999_MOESM2_ESM.pdf]

nature portfolio

Corresponding author(s): Isabelle Ray-Coquard

Last updated by author(s): 6 October 2023

Reporting Summary

Nature Portfolio wishes to improve the reproducibility of the work that we publish. This form provides structure for consistency and transparency in reporting. For further information on Nature Portfolio policies, see our Editorial Policies and the Editorial Policy Checklist.

Statistics

For all statistical analyses, confirm that the following items are present in the figure legend, table legend, main text, or Methods section.

n/a	Confirmed	
☐	☒	The exact sample size (n) for each experimental group/condition, given as a discrete number and unit of measurement
☒	☐	A statement on whether measurements were taken from distinct samples or whether the same sample was measured repeatedly
☒	☐	The statistical test(s) used AND whether they are one- or two-sided<br>*Only common tests should be described solely by name; describe more complex techniques in the Methods section.*
☒	☐	A description of all covariates tested
☒	☐	A description of any assumptions or corrections, such as tests of normality and adjustment for multiple comparisons
☐	☒	A full description of the statistical parameters including central tendency (e.g. means) or other basic estimates (e.g. regression coefficient) AND variation (e.g. standard deviation) or associated estimates of uncertainty (e.g. confidence intervals)
☒	☐	For null hypothesis testing, the test statistic (e.g. F, t, r) with confidence intervals, effect sizes, degrees of freedom and P value noted<br>*Give P values as exact values whenever suitable.*
☒	☐	For Bayesian analysis, information on the choice of priors and Markov chain Monte Carlo settings
☒	☐	For hierarchical and complex designs, identification of the appropriate level for tests and full reporting of outcomes
☒	☐	Estimates of effect sizes (e.g. Cohen's d, Pearson's r), indicating how they were calculated

Our web collection on statistics for biologists contains articles on many of the points above.

Software and code

Policy information about availability of computer code

Data collection	CS Online -> Ennov Clinical version v8.2.50
Data analysis	SAS software, SAS v9.4 (64 bytes)

For manuscripts utilizing custom algorithms or software that are central to the research but not yet described in published literature, software must be made available to editors and reviewers. We strongly encourage code deposition in a community repository (e.g. GitHub). See the Nature Portfolio guidelines for submitting code & software for further information.

Data

Policy information about availability of data

All manuscripts must include a data availability statement. This statement should provide the following information, where applicable:

- Accession codes, unique identifiers, or web links for publicly available datasets
- A description of any restrictions on data availability
- For clinical datasets or third party data, please ensure that the statement adheres to our policy

Data sharing in a public repository was not planned at the beginning of the study and currently no mechanism is in place to allow sharing of individual deidentified patient data. Requests sent to ARCAGY-GINECO (bvotan@arcagy.org) will be considered on a case-by-case basis.

Research involving human participants, their data, or biological material

Policy information about studies with human participants or human data. See also policy information about sex, gender (identity/presentation), and sexual orientation and race, ethnicity and racism.

Reporting on sex and gender	In this trial in ovarian cancer (including fallopian tube or peritoneal cancer), the protocol inclusion criteria specified women so this is now stated in the title.
Reporting on race, ethnicity, or other socially relevant groupings	Information on race and ethnicity was not collected in this relatively small single-country study.
Population characteristics	This information is provided in Supplementary Table 1.
Recruitment	Patients were recruited by the investigators according to the inclusion/exclusion criteria of the study protocol (no recruitment by internet, no recruitment by advertisement). There was no compensation for participation.
Ethics oversight	CPP Nord Ouest II

Note that full information on the approval of the study protocol must also be provided in the manuscript.

Field-specific reporting

Please select the one below that is the best fit for your research. If you are not sure, read the appropriate sections before making your selection.

[x] Life sciences [ ] Behavioural & social sciences [ ] Ecological, evolutionary & environmental sciences

For a reference copy of the document with all sections, see nature.com/documents/nr-reporting-summary-flat.pdf

Life sciences study design

All studies must disclose on these points even when the disclosure is negative.

Sample size	Assuming a 50% CRR at IDS with NACT alone, as reported in the literature,1,4 the planned sample size of 90 patients (60 in the experimental investigational arm, 30 in the standard-of-care control arm) was calculated based on the A'Hern single-stage design,36 with a ≥70% success rate (CC0 at IDS) in the pembrolizumab arm considered sufficient to justify further evaluation and a <50% rate considered insufficient.
Data exclusions	No data were excluded; analyses were performed according to intent-to-treat principles.
Replication	This was a randomized trial with stratification factors
Randomization	Patients were randomized in a 1:2 ratio using a web-based system (Euraxipharma, https://ecrf.euraxipharma.fr/CSOnline/) and a minimization procedure to receive chemotherapy alone (standard-of-care control arm) or combined with pembrolizumab (experimental investigational arm).
Blinding	This was an open-label randomized phase II trial. Blinding was not considered necessary as this was a non-comparative trial.

Reporting for specific materials, systems and methods

We require information from authors about some types of materials, experimental systems and methods used in many studies. Here, indicate whether each material, system or method listed is relevant to your study. If you are not sure if a list item applies to your research, read the appropriate section before selecting a response.

Materials & experimental systems

n/a	Involved in the study	
[x]	[ ]	Antibodies
[x]	[ ]	Eukaryotic cell lines
[x]	[ ]	Palaeontology and archaeology
[x]	[ ]	Animals and other organisms
[ ]	[x]	Clinical data
[x]	[ ]	Dual use research of concern
[x]	[ ]	Plants

Methods

n/a	Involved in the study	
[x]	[ ]	ChIP-seq
[x]	[ ]	Flow cytometry
[x]	[ ]	MRI-based neuroimaging

Clinical data

Policy information about clinical studies

All manuscripts should comply with the ICMJE guidelines for publication of clinical research and a completed CONSORT checklist must be included with all submissions.

Clinical trial registration	Clinicaltrials.gov number NCT03275506
Study protocol	The protocol will be provided in the supplementary materials
Data collection	Between February 26, 2018, and April 17, 2019, 91 patients were enrolled from 17 sites in France. Data were collected in academic centers in an electronic case report form (CS Online Ennov Clinical version v8.2.50 powered by Euraxi Pharma, a French contract research organization). The data were monitored through on-site monitoring visits by clinical research associates according to a prespecified monitoring plan. All data were centralized in a database that was handled and controlled according to a specific data management plan, and analyzed using SAS version 9.4
Outcomes	The primary endpoint was CRR, defined as the removal of all macroscopic residual tumor (CCI = 0; CC0) at IDS, as assessed by blinded independent centralized review by two surgical experts and the coordinating investigator, who reviewed the anonymized operative and pathologic reports of all patients at screening, at IDS, and at other debulking surgery.<br>Secondary efficacy endpoints included CCI score by local assessment, PCI score by local and central assessment (to be reported separately), ORR after four neoadjuvant cycles according to RECIST version 1.1, best overall response to the global strategy, PFS according to RECIST version 1.1 (defined as the interval between randomization and date of disease progression or death, whichever occurred first), and OS. Other secondary endpoints included safety during NACT and in the adjuvant setting, postoperative mortality, and postoperative morbidity according to modified Clavien-Dindo classification.

Plants

Seed stocks	*Report on the source of all seed stocks or other plant material used. If applicable, state the seed stock centre and catalogue number. If plant specimens were collected from the field, describe the collection location, date and sampling procedures.*
Novel plant genotypes	*Describe the methods by which all novel plant genotypes were produced. This includes those generated by transgenic approaches, gene editing, chemical/radiation-based mutagenesis and hybridization. For transgenic lines, describe the transformation method, the number of independent lines analyzed and the generation upon which experiments were performed. For gene-edited lines, describe the editor used, the endogenous sequence targeted for editing, the targeting guide RNA sequence (if applicable) and how the editor was applied.*
Authentication	*Describe any authentication procedures for each seed stock used or novel genotype generated. Describe any experiments used to assess the effect of a mutation and, where applicable, how potential secondary effects (e.g. second site T-DNA insertions, mosiacism, off-target gene editing) were examined.*
